# The flax genome reveals orbitide diversity

**DOI:** 10.1186/s12864-022-08735-x

**Published:** 2022-07-23

**Authors:** Ziliang Song, Connor Burbridge, David J. Schneider, Timothy F. Sharbel, Martin J. T. Reaney

**Affiliations:** 1 Department of Plant Sciences, College of Agriculture and Bioresources, University of Saskatchewan, 51 Campus Drive, Saskatoon, SK S7N 5A8 Canada; 2 Global Institute for Food Security, University of Saskatchewan, Saskatoon, SK S7N 4L8 Canada; 3 School of Environment and Sustainability, University of Saskatchewan, Saskatoon, SK S7N 5C8 Canada; 4 Department of Food and Bioproduct Sciences, University of Saskatchewan, Saskatoon, SK S7N 5A8 Canada; 5 Guangdong Saskatchewan Oilseed Joint Laboratory, Department of Food Science and Engineering, Jinan University, Guangzhou, Guangdong 510632 China

**Keywords:** Orbitide, *Linum usitatissimum*, Protein tandem repeats, Mining, Diversity

## Abstract

**Background:**

Ribosomally-synthesized cyclic peptides are widely found in plants and exhibit useful bioactivities for humans. The identification of cyclic peptide sequences and their precursor proteins is facilitated by the growing number of sequenced genomes. While previous research largely focused on the chemical diversity of these peptides across various species, there is little attention to a broader range of potential peptides that are not chemically identified.

**Results:**

A pioneering study was initiated to explore the genetic diversity of linusorbs, a group of cyclic peptides uniquely occurring in cultivated flax (*Linum usitatissimum*). Phylogenetic analysis clustered the 5 known linusorb precursor proteins into two clades and one singleton. Preliminary tBLASTn search of the published flax genome using the whole protein sequence as query could only retrieve its homologues within the same clade. This limitation was overcome using a profile-based mining strategy. After genome reannotation, a hidden Markov Model (HMM)-based approach identified 58 repeats homologous to the linusorb-embedded repeats in 8 novel proteins, implying that they share common ancestry with the linusorb-embedded repeats. Subsequently, we developed a customized profile composed of a random linusorb-like domain (LLD) flanked by 5 conserved sites and used it for string search of the proteome, which extracted 281 LLD-containing repeats (LLDRs) in 25 proteins. Comparative analysis of different repeat categories suggested that the 5 conserved flanking sites among the non-homologous repeats have undergone convergent evolution driven by functional selection.

**Conclusions:**

The profile-based mining approach is suitable for analyzing repetitive sequences. The 25 LLDR proteins identified herein represent the potential diversity of cyclic peptides within the flax genome and lay a foundation for further studies on the functions and evolution of these protein tandem repeats.

**Supplementary Information:**

The online version contains supplementary material available at 10.1186/s12864-022-08735-x.

## Background

Plant secondary metabolites play important roles in plant development, mediating communication with other organisms, responding to biotic stresses, and acclimating to the abiotic environment [1]. While primary metabolites are similar in most plant cells, plant secondary metabolites are enormously diverse, varying across and within species. Conventional classification of secondary metabolites is generally based on chemical structure, with alkaloids, phenolics, terpenoids, and non-ribosomal peptides (NRPs) being the major groups. These well-known compounds are synthesized from primary metabolites through specialized metabolic pathways. Advances in genome sequencing technologies over the past decade have revealed that many structurally unique peptides are synthesized from ribosomally produced precursor peptides that undergo post-translational modification (RiPPs), rather than by the action of non-ribosomal peptide synthetases (NRPS) [2]. Like other currently known secondary metabolites, RiPPs exhibit species specificity and are thus recognized as a new group of secondary metabolites.

**Fig. 1:**
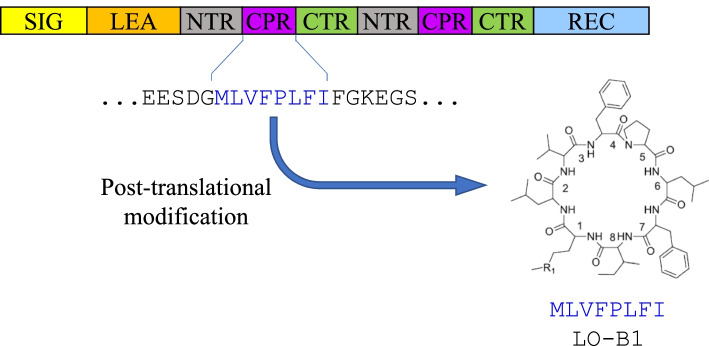
General biosynthetic pathway of orbitides, abstracted from [3]. The precursor protein mainly comprises a signal sequence (SIG, yellow), a leader peptide (LEA, orange), the core peptide region (CPR, purple) and the recognition sequence (REC, blue). Each CPR is flanked by the N-terminal region (NTR, grey) and C-terminal region (CTR, green). The precursor protein undergoes post-translational modification that cyclizes the CPR into the mature cyclized product. In this case, the CPR is a linusorb B1 (LO-B1) domain in which the N-terminal methionine (M) and C-terminal isoleucine (I) are linked to form the structural formula as displayed

One typical form of RiPPs are cyclic peptides, which are derived from linear precursor proteins which are cyclized by specialized proteases (Fig. 1). Well characterized classes of cyclic peptides thus far identified include head-to-tail (i.e. N-to-C) linked peptides and branched cyclic peptides. Based on the number of disulfide bonds, head-to-tail linked cyclic peptides are categorized into cyclotides (3 disulfide bonds), PawS-derived peptides (PDPs, 1 disulfide bond) and orbitides (no disulfide bond). Many cyclic peptides have been discovered from plant species traditionally used in health and medicinal applications, such as *Oldenlandia affinis* (Violaceae) and *Lycium barbarum* (Solanaceae). A wide range of biological activities have been reported in cyclic peptides, including enzyme (trypsin) inhibition, immunosuppression, anti-inflammation, antibacterial activity and antiviral effects [4–10]. It is postulated that plants produce cyclic peptides as a defense agent against herbivores, insects and pathogens [11–13].

Since the isolation of the first cyclic peptide, cyclolinopeptide A, from the sediment of flaxseed oil in 1959 [14], more than 400 cyclic peptides had been discovered in higher plants across 120 species and 26 families [15]. The discovery of novel cyclic peptides is guided by knowledge of the peptides in related plant species and families. Cyclotides were originally found in the violet family (Violaceae). In 2008, Gruber et al. developed a screening procedure based on the hydrophobicity, molecular weight and cysteine content of violet cyclotides to analyze > 200 Rubiaceae species (the coffee family) and > 140 species in related families [16]. This led to the discovery of cyclotides in 22 Rubiaceae species. In addition to tracing peptides with similar physicochemical properties, sequence similarity is also a useful trait for novel peptide exploration. For example, a gene-guided approach was applied to mining the genomes of multiple plant species for orthologues of the precursor protein of lyciumin, a branched cyclic peptide primarily isolated from the root of Chinese wolfberry (*Lycium barbarum*) [17]. Selecting protein candidates harboring the BURP domain found in the lyciumin precursor protein assisted in the detection and verification of predicted lyciumin peptides in 10 plant species from 4 families.

Comparative genomics analyses identified potential orthologues of individual precursor proteins, although it has been shown that precursor proteins embedded with cyclic peptides of the same structural class (cyclotides) can have different (i.e. non-homologous) biosynthetic origins [18]. These findings indicate untapped diversity in precursor proteins or structural motifs corresponding to specific biosynthetic and post-translational modification pathways. Furthermore, while previous research focused on the chemical diversity of ribosomally-derived cyclic peptides, there remains limited information regarding their genetic characteristics.

Another potential limitation lies in the chemical verification of predicted peptides. Hellinger et al. (2015) conducted a tBLASTn search in the transcriptome of *Viola tricolor* using a set of published cyclotide sequences as queries, which revealed 108 transcripts with homology to the cyclotide sequences [19]. Of these, 11 peptides were unequivocally verified by both mass spectroscopic signal deconvolution and MS/MS sequencing. Similar results were obtained in the transcriptome mining of 6 *Viola* species [20]. The question thus remains whether unverified peptide sequences contain genetic information relevant to the evolution of verified peptides. Identification of peptides relies heavily on empirical knowledge regarding the physicochemical properties of known peptides, and thus presents many technical challenges. While the past decade has seen a significant growth in the number of cyclic peptides discovered in a variety of species, current mining practices largely focus on and are limited to the identified peptides.

In this study, we developed proteome mining methods to explore the genetic diversity of orbitides in flax (*Linum usitatissimum* L., 2*n* = 2*x* = 30). Flax is one of the most ancient crops widely grown as a source of fiber, oil, and health products. More than 20 orbitides have been isolated from flaxseed and are collectively named linusorbs [21]. Previous homology searches of both an EST library [22] and reference genome [23, 24] using partial sequence information from mass spectra as queries led to the identification of linusorb precursor proteins [25–27]. The reported linusorbs are structural variants derived from domains embedded in 5 precursor proteins (Fig. 1), while the relationships among these linusorb domains and among their precursor proteins remain unclear. Here, we first analyzed these relationships, and designed mining strategies to identify sequences at different similarity levels. The wide cultivation of flax as a crop makes it an ideal subject for the study of cyclic peptide diversity, and thus the goal of this study is to elucidate genetic diversity independent of what can be chemically verified. Finally, we discuss how these diverse but related sequences evolve within a plant genome.

## Results

### The linusorb precursor proteins form 2 distinct clades and a singleton

**Fig. 2:**
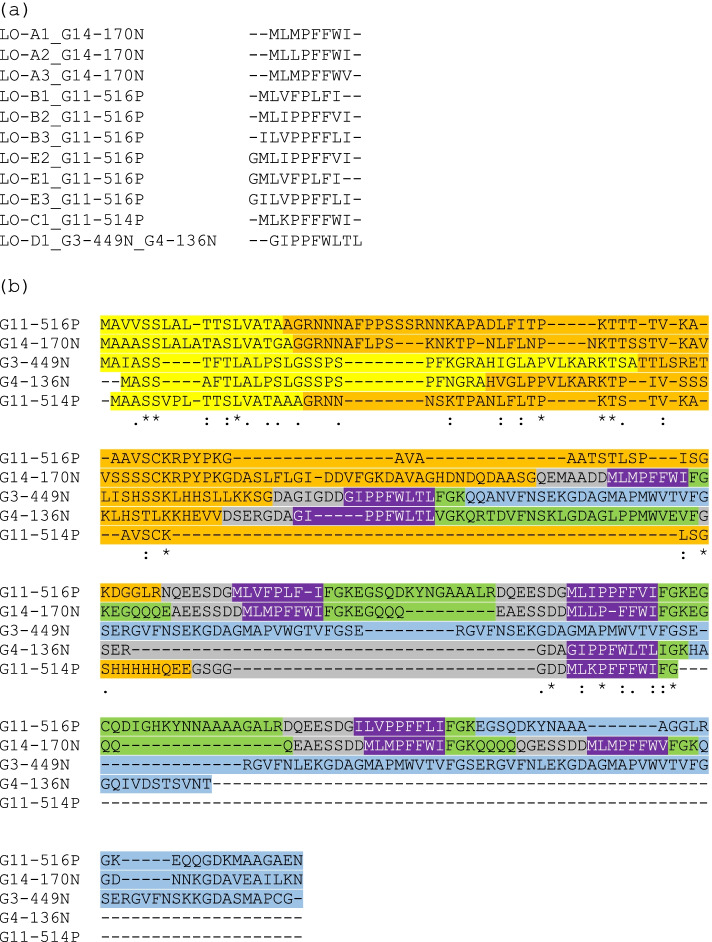
Multiple sequence alignments of (**a**) 11 linusorb domains and (**b**) 5 linusorb precursor proteins. Different regions are shaded in different colors in accordance with the coloring scheme of Fig. 1

**Fig. 3:**
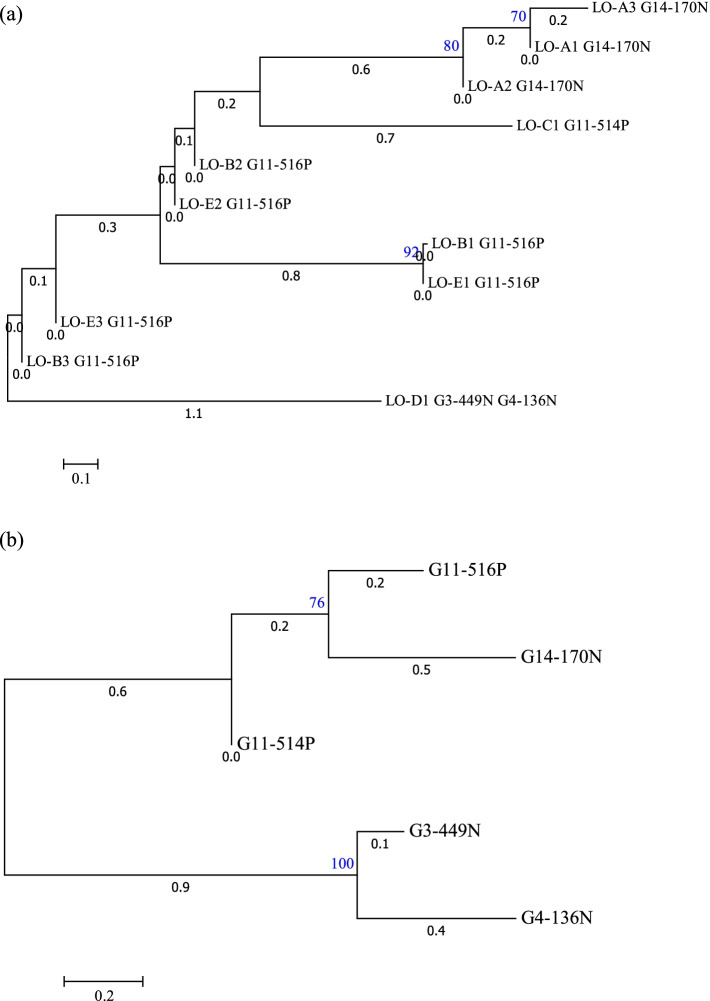
Phylogenetic trees of (**a**) 11 linusorb (LO) domains and (**b**) 5 linusorb precursor proteins. Neighbor-Joining method was used to cluster the sequences aligned by MUSCLE. Numbers in blue above the nodes represent the bootstrap values of 1000 replications. Only nodes with bootstrap values ≥60 are considered significant and have their bootstrap values displayed. Numbers in black below the nodes represent the branch lengths, i.e. genetic distance between two nodes

The relationships among the linusorb domains and among the linusorb precursor proteins were investigated based on similarities revealed by multiple sequence alignments (Fig. 2). There are 3 significant clades in the phylogenetic tree of the 11 linusorb domains computed by Neighbor-Joining (NJ) method (Fig. 3a). The first 2 clades cluster all 3 linusorb domains in G14-170 N, suggesting linusorb domains within the same protein are more closely related than across proteins. The third clade contains LO-B1 and its glycine-appended analogue LO-E1 in G11-516P. We previously reported that each linusorb domain in G11-516P have 2 analogues detected, one with and one without the glycine at the N-terminus [27]. Such high similarity accounts for the fact that the 2 linusorb analogues are clustered as one clade. Likewise, other linusorb analogues in G11-516P (LO-B2&E2, LO-B3&E3) are close to each other albeit not forming significant clades.

The 5 linusorb precursor proteins form 2 significant clades and a singleton (Fig. 3b). The upper clade contains G11-516P and G14-170 N, while the lower clade contains G3-449N and G4-136N. The singleton, G11-514P, is distinctly isolated from both clades. As proteins within a clade are potential homologues, note that the homologous pair G3-449N and G4-136N share the same linusorb domain, LO-D1. Considering the short lengths of linusorb domains and the variable lengths of linusorb precursor proteins (Table 1), phylogenetic trees were also built with the Maximum Likelihood (ML) method in addition to the NJ method to verify the relationship (Fig. S1). For the known linusorb domains, both NJ and ML trees share similar topology and significant clades (Fig. S1a). For the 5 linusorb precursor proteins, the ML tree has its topology consistent with the NJ tree but the clade containing G14-170 N and G11-516P is less significant (Fig. S1b).

**Table 1:** Lengths and numbers of linusorb domains in the 5 linusorb precursor proteins

Precursor protein	Total length (aa)	# of linusorb domains	Average linusorb domain length
G11-516P	204	3	8.7 ± 0.6
G11-514P	76	1	9 ± 0
G14-170 N	219	5	8 ± 0
G3-449N	223	1	9 ± 0
G4-136N	129	2	9 ± 0

### No other significant homologues were found besides the 5 precursor proteins

We first followed the conventional gene-guided mining approach using tBLASTn to search the genome for other homologous sequences of the 5 linusorb precursor proteins. The search outputs with alignments between the query and subject are provided as Data S1. No additional homologues were identified other than the original 5 proteins. The potential homology is consistent with that illustrated by the phylogenetic tree in Fig. 3a. The singleton G11-514P does not have any significant hit besides itself, but interestingly the second closest hit turns out to be G14-170 N, which is consistent with the ML tree where G11-516P, G14-170 N and G11-514P are not significantly isolated from one another (Fig. S1b). We compared the protein alignment constructing the tree (Fig. 2b) with the local alignment output from BLAST, and detected differences between both alignments. Among the 6 local alignment ranges between G11-514P and G14-170 N identified by BLAST, the first range matches the signal peptide region of both proteins and the other 5 ranges align the single linusorb-embedded region of G11-514P with the 5 linusorb-embedded regions in G14-170 N, respectively. Among the latter 5 alignment ranges, the one with the region containing LO-A1 (MLMPFFWI) scores the highest (34.0 bits). However, in the global alignment of linusorb precursor proteins (Fig. 2b), LO-C1 of G11-514P is aligned to LO-A2 (MLLPFFWI) rather than LO-A1, leaving a big gap at the position of LO-A1. Such alignment discrepancy is due to the different nature of alignment algorithms, where one is multiple sequence alignment of the whole proteins and the other is pairwise alignment of local sequence fragments within the protein. This further suggests that the accuracy of whole protein alignment is susceptible to large variation in protein length and the number of linusorb domains (Table 1).

### Linusorb domains are embedded in repeat structures and flanked by conserved residues

**Fig. 4:**
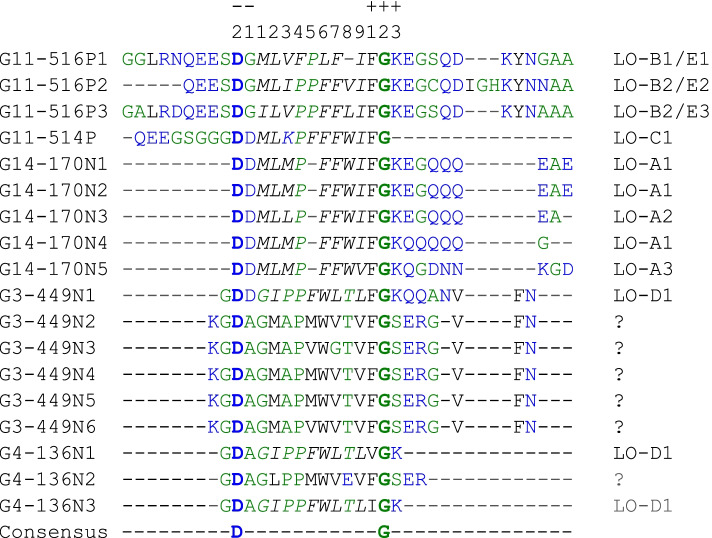
Multiple sequence alignment of repeats identified in the 4 precursor proteins and an extended region of G11-514P containing the single linusorb domain with some flanking residues. Repeats are numbered on the left of the alignment and the name of linusorb domain in each repeat is shown on the right, while repeats containing undetected linusorb-like domains (LLDs) are marked by “?”. Linusorb domain sequences are italicized in the alignment, except LOs E1 – E3, the 3 glycine-containing analogues of LOs B1 – B3, which are underlined. Consensus sites flanking the linusorb(−like) domains are highlighted in bold. The scale on the top marks the starting position (0) of the linusorb domain, and the flanking sites are numbered as minus towards the N-terminus and as plus towards the C-terminus. Colors of amino acids employed the hydropathicity color scheme in which hydrophobic amino acids (YVMCLFIW) are colored black, hydrophilic (RKDENQ) are blue and neutral (SGHTAP) are green

Given that 4 of the 5 precursor proteins contain multiple linusorb domains, we hypothesized that these linusorb domains are in repeat structures. Analysis of protein sequences by RADAR detected multiple repeats in 4 proteins (Data S2). Alignment of the identified repeats and G11-514P which contains a single linusorb domain reveals a conserved pattern in the sequences flanking the core linusorb domains (Fig. 4). Each linusorb domain is embedded in a repeat unit and flanked by conserved residues. The most conserved residues are D at the − 2 position and G at the + 2 position, as highlighted in bold. Some repeats of G3-449N and G4-136N have no orbitides identified chemically and thus are marked by “?” in Fig. 4. Nonetheless, with such repetitive pattern it is reasonable to specify the sequence aligned with the known linusorb domains in these repeats as linusorb-like domain (LLD), e.g., GMAPMWVTV in G3-449N. Importantly, the flanking sites between linusorb domains and LLDs differ at position + 3, with K and S in the former and latter respectively. The linusorb precursor protein G11-514P is the only exception where K is substituted by a stop codon at the + 3 position.

Apart from these 3 absolutely conserved sites in the repeats of known linusorb domains, sites immediately flanking the linusorb domains are conserved at lower levels: the − 1 position is equally shared by D, A and G; the + 1 position is dominated by F, with I and V as minor amino acids (Fig. 4). Note that the − 1 G is part of the linusorb domains in LOs E1 – E3 as the G-appended analogues of LOs B1 – B3. Here the cleavage site is between the − 2 D and − 1 G instead of C-terminal to the − 1 G as seen in most linusorb cases. This rare exception is likely the result of alternative cleavage by the protease or another type of protease with a different cleavage specificity. Nevertheless, both LOs B1 – B3 and their G-appended analogues LOs E1 – E3 share the same repeats, and thus this conserved pattern remains unchanged in the alignment regardless of the alternative cleavage scheme.

### Genome re-annotation retained repeat-containing proteins for database mining

One limitation of tBLASTn lies in the uncharacterized subject sequence retrieved from the directly-translated genome. The tBLASTn tool directly translates the genome in 6 frames and reports any locally-aligned sequences. It remains unknown as to whether the matching subject is in the coding or non-coding region. To focus mining onto only coding sequences, it is necessary to annotate the genome and use the predicted proteome as the database for mining. The previously annotated flax reference genome [23] contains 43,484 predicted genes, among which none of the 5 linusorb precursor proteins were identified, probably due to repeat masking before annotation. Our annotation scheme without repeat masking using BRAKER2 predicted a total of 57,156 genes which contained 4 of the 5 linusorb precursor proteins. Only G11-514P, the single-linusorb precursor protein with a length of 77 aa, was not identified.

Unique from the other 4 repeat-containing counterparts, G11-514P implies a larger number of protein candidates embedded with a single linusorb or linusorb-like domain encoded in the genome. They are unrecognized by BRAKER2 probably due to short length or unusual structure. To maximize the coverage of putative proteins, we mined the virtual ORF library generated by the EMBOSS tool *getorf* as it can capture every possible start codon without relying on a specified gene model. Expectedly, the virtual ORF library contains G11-514P, with the same ORF sequence terminated after LO-C1 and two flanking residues FG at the C-terminus. The other 4 repeat-containing linusorb precursor proteins have identical sequences except G14-170 N lacking the first exon translate, as *getorf* does not interpret splice-sites.

### Profile-based search for LLDs

The presence of repeats and their variable number in the linusorb precursor proteins might pose a challenge for whole protein alignment, and dissecting multiple repeat regions into individual repeat units might solve this problem. Aligning repeats across different proteins enables the identification of both conserved and variable regions within the repeats. Potential homology can occur not only at the whole protein level but also at the repeat level, and thus it would be useful to identify homologous sequences of the linusorb-embedded repeats in the proteome. Thus, our mining strategy was transformed from sequence-based to profile-based. The following sections report the results of two mining strategies: (1) an iterative search for homologous sequences of linusorb-embedded repeats using algorithm-derived profiles, and (2) a string search for sequences matching profiles composed of conserved flanking sites. Both strategies are based on different assumptions and provide unique perspectives for exploring linusorb-like sequence diversity.

#### Iterative searches with algorithm-derived profiles

Iterative searches can efficiently identify sequences with varying homology. Such searches begin with the generation of profiles from the set of linusorb-embedded repeats which are then used as queries to search the proteome for similar sequences. Significant matches are combined to update the profile which is then used in the next round of search. The search is iterated until no more new sequences are found within the significance threshold. Two models were used to describe profiles. The Position Specific Score Matrix (PSSM) is based on the frequencies of each residue in a specific position of a multiple sequence alignment and is used as query in the Position Specific Iterative BLAST (PSI-BLAST) search. The second is a probabilistic “profile hidden Markov model” (profile HMM) trained from a multiple alignment and used as query to search a sequence database using the HMMERsuite. The mining results of both iterative search methods are as follows:

##### PSI-BLAST search with PSSM

The PSSM used for PSI-BLAST was built from the multiple sequence alignment of 12 linusorb-embedded repeats (marked with the linusorb name at the end of the repeat in Fig. 4). Using the PSSM as a query, the first round of PSI-BLAST retrieved 4 linusorb precursor proteins as the top 4 matches (Data S3). The first two matches are G11-516P (proteome ID: g24919) and G14-170 N (g7437) with E-values of 3e^− 10^ and 1e^− 4^, respectively. The third and fourth matches are G3-449N (g53356) and G4-136N (g33422) with E-values of 0.006 and 0.007, respectively. The reason for this order of matches is that the first sequence in the multiple alignment (G11-516P1 in Fig. 4) used to build the PSSM was adopted as the “master” sequence by PSI-BLAST and then used as the representative query sequence for the alignment with all subject sequences in the output matches. It is worth reminding that the query for PSI-BLAST search is PSSM rather than the individual repeat G11-516P1. Nevertheless, the ranking order of matches along with the large gap of E-values between the second and third matches indicates that the repeats between proteins from the same clade (G11-516P and G14-170 N, G3-449N and G4-136N; Fig. 3b) are more closely related than between those from different clades (e.g. G14-170 N and G3-449N; Fig. 3b). The same pattern of matches was reproduced when repeats of proteins from the other clade, e.g. G3-449N1, were placed as the first sequence in the training alignment set for PSSM (data not shown). This suggests that the relationship among the repeats is consistent with their precursor proteins, in that repeats of proteins from different clades are more distantly related as indicated by their higher E-values (Fig. 3b).

The remaining matches after the top 4, starting with an E-value of 0.48, are unknown proteins (Data S3). Despite the lack of significance in most circumstances, all these alignments are anchored by several conserved sites with high PSSM values: the D at − 2 position in the starting alignment, F and G at + 1 and + 2 positions, respectively. The search converged after the second round which retrieved two significant matches, namely G11-516P and G14-170 N, as well as two insignificant matches that were not found in the previous round. The two insignificant matches also contain conserved flanking sites, especially consecutively at the C-terminus (FGK), highlighting the weight of these sites in the PSSM computed from the candidates of the first round.

##### HMMER search with profile HMM

HMMER is known for its sensitivity in detecting even more remote homologues than PSI-BLAST because its algorithms employ probability models that take into account the transitions of states (match, insertion, deletion) within one site and across adjacent sites in the alignment, while PSSM assumes all sites are independent of one another [28]. A profile HMM built by *hmmbuild* on the alignment of 12 linusorb-embedded repeats (Fig. 4) was used as a query for the search of proteome by *hmmsearch*. Protein matches above the default inclusion threshold were considered significant. An iterative search was performed manually by querying the proteome with an updated profile HMM built from the alignment of all matches from previous rounds until no more new matches were found. Three rounds of *hmmsearch* retrieved 8 new proteins in addition to the original linusorb precursor proteins (Data S4). Of the 8 proteins, only 2 were retrieved by PSI-BLAST, and these 2 matches in PSI-BLAST lack significance due to high E-values (> 0.01). This reflects that PSI-BLAST and HMMER employ different pattern recognition algorithms and schemes for scoring sequence alignments.

Within the 8 protein matches there are 58 motifs significantly matching the profile HMM, as the E-values are lower than both per-sequence and per-domain inclusion thresholds (Data S4). HMMER considers a match to be “true” by first looking for the independent E-value (per-sequence threshold) of 0.01 or less, and if the independent E-value is between 0.01 and 1, then checking for the conditional E-value (per-domain threshold) of 0.01 or less [29]. As determined by RADAR, all these 8 proteins contain multiple (> 2) repeats.

**Fig. 5:**
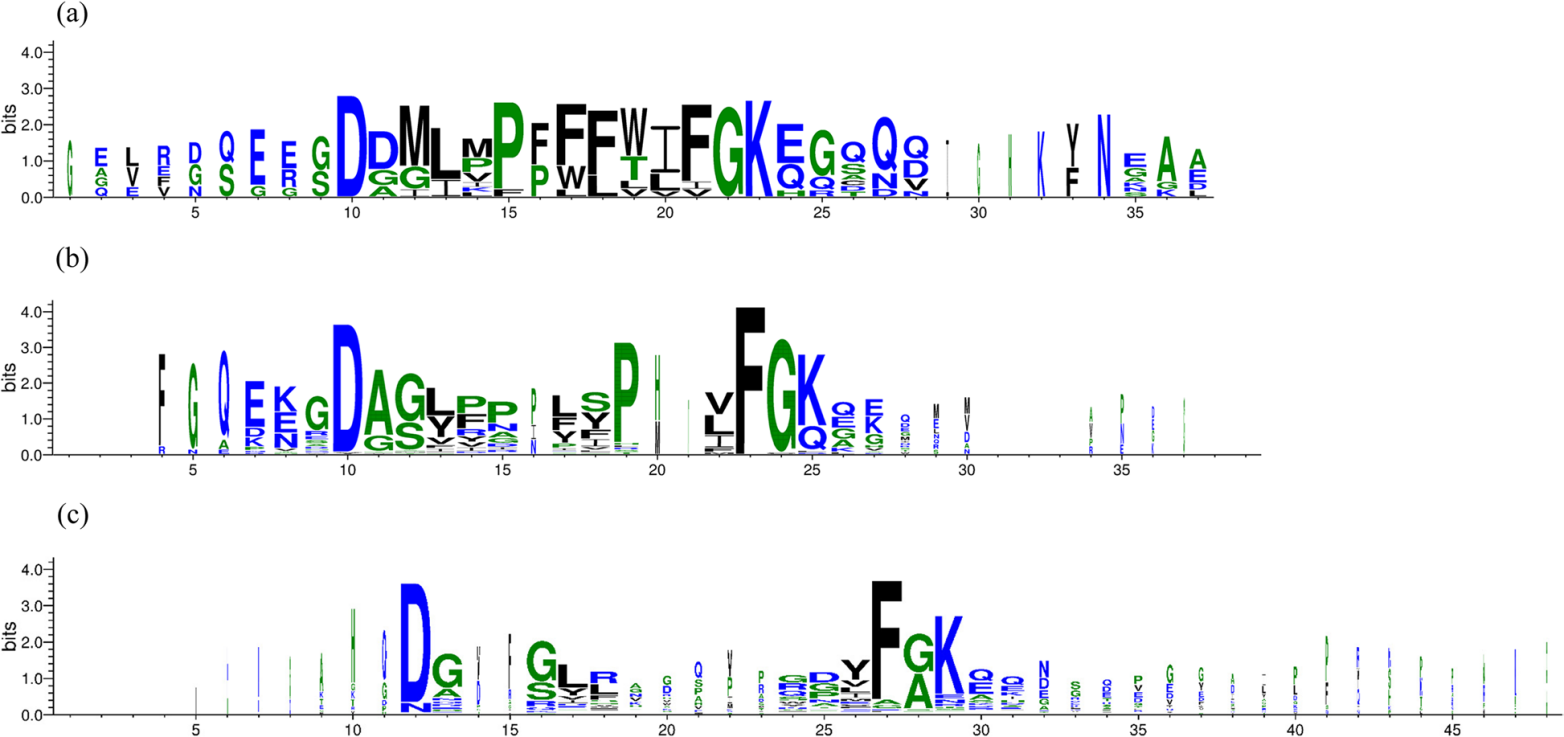
Sequence logos of (**a**) 12 linusorb-embedded repeats of the 5 known linusorb precursor proteins; (**b**) 58 potential homologues from 8 LLDR proteins retrieved by HMM search (Data S4); (c) 223 non-homologous repeats in 25 LLDR proteins extracted by pattern-matching string search

All 58 motifs retrieved by *hmmsearch* are members of the identified repeats, and thus are potential homologues of the 12 linusorb-embedded repeats in Fig. 4. Since the number of matches is large, sequence logos were created from the 12 linusorb-embedded repeats (Fig. 5a) and the 58 LLD-containing repeats (LLDRs) (Fig. 5b). The logos are anchored by a few larger icons representing the most conserved residues. Both groups of motifs share two absolutely conserved residues: D at − 2 site (the 10th residue on both scales) and G at + 2 site (the 22nd residue in Fig. 5a and the 23rd residue in Fig. 5b). The linusorb-embedded repeats have another absolutely conserved K at + 3 site (23rd residue in Fig. 5a), while the + 3 site in the LLDRs are shared by K and Q. The F at + 1 site is dominant in linusorb-embedded repeats but absolutely conserved in LLDRs. These interesting contrasts suggest that F at + 1 site is more prevalent in these linusorb-related motifs (both linusorb and linusorb-like) while I and V as minor occurrences may be the result of DNA mutations in the first position of the codon (UU[UC] = F, AU[UCA] = I, GU[UCAG] = V). Likewise, point mutations may account for the introduction of Q (GA[AG]) from the dominant K (AA[AG]), as these amino acids are coded by highly similar codons. Another difference lies in the − 1 site, where the LLDRs are equally shared by D, G and A, but the LLDRs are dominated by A. The LLDs in the repeats (residues 12–21 in Fig. 5b) are highly diverse and share few residues with the linusorb domains (residues 12–20 in Fig. 5a). This is consistent with the hypervariability generally found in the known linusorbs and other cyclic peptides within a species [30–32].

#### Proteome mining based on profile-matching string search for LLDs

As revealed in the above two algorithm-based searches, potential homologues of the linusorb-embedded repeats are characterized of (1) repetitiveness, (2) variable LLDs and (3) conserved flanking residues. The conservation of flanking sites has been reported to play a vital role in the recognition by modification enzymes during the biosynthesis of cyclic peptides in other species [30, 33–38]. The above two algorithms, both PSI-BLAST and HMM, are designed to find potential homologues of the entire repeat. This means that while a few highly conserved flanking sites have the most weight on the profile, other non-random sites in the repeat, such as the linusorb domain and some less conserved flanking sites at both termini, also account for the profile. Since the known linusorb domains are hypervariable, it would be useful to randomize the LLD in the profile.

**Fig. 6:**
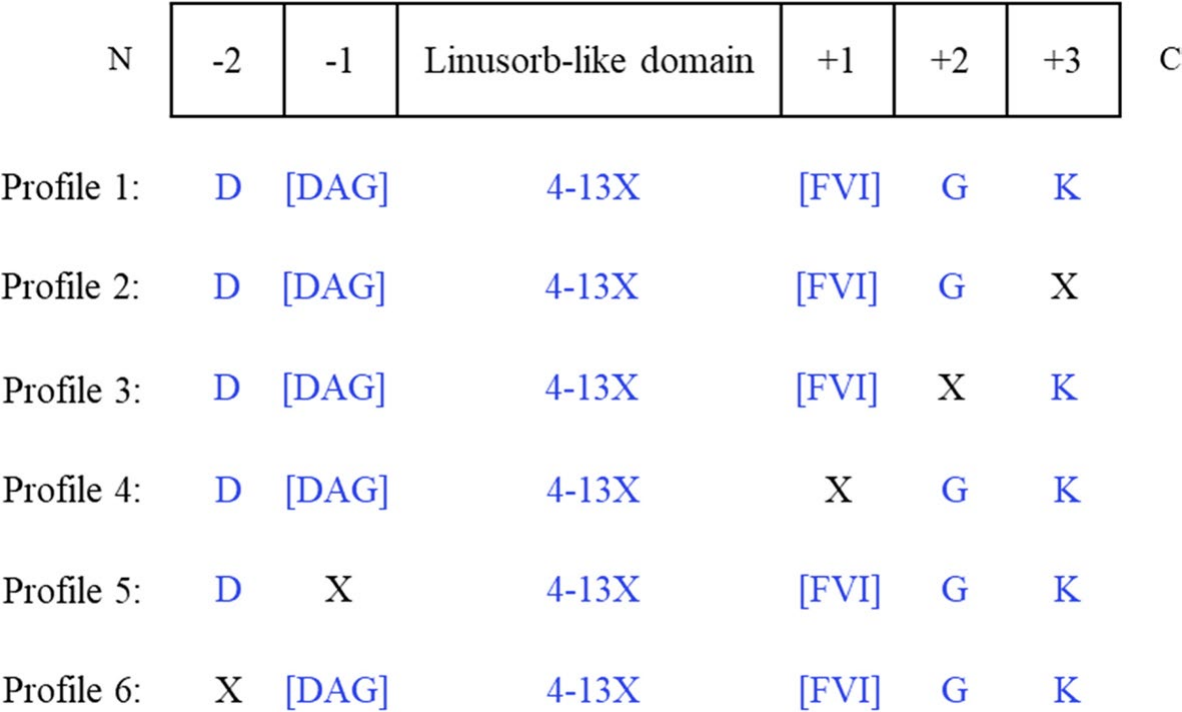
Profiles designed for the search of possible LLDs. Different conserved sites were specified: Profile 1 has all 5 sites specified; Profiles 2–6 alternate one random site for each

We thus developed a mining method focused on flanking site conservation by generating 26 profiles to characterize an LLDR (Table S1). These 26 profiles cover all combinations of residues at the 5 most conserved flanking sites discussed above. As illustrated in Fig. 6, conservation of the 5 flanking signature sites exhibited variability: 3 absolutely conserved residues, namely D (− 2), G (+ 2) and K (+ 3), and 2 less conserved sites contiguous to the linusorb domain: [DAG] (− 1) and [FVI] (+ 1). The region corresponding to the linusorb domain was left random to maximize its sequence variability. Nevertheless, considering the 8 identified linusorbs contain 8 or 9 residues (average length = 8.5), the length of possible linusorb domains was set at a range from 4 to 13 residues (average length ± 4) as a reasonable length of published orbitides [31, 39–41].

**Fig. 7:**
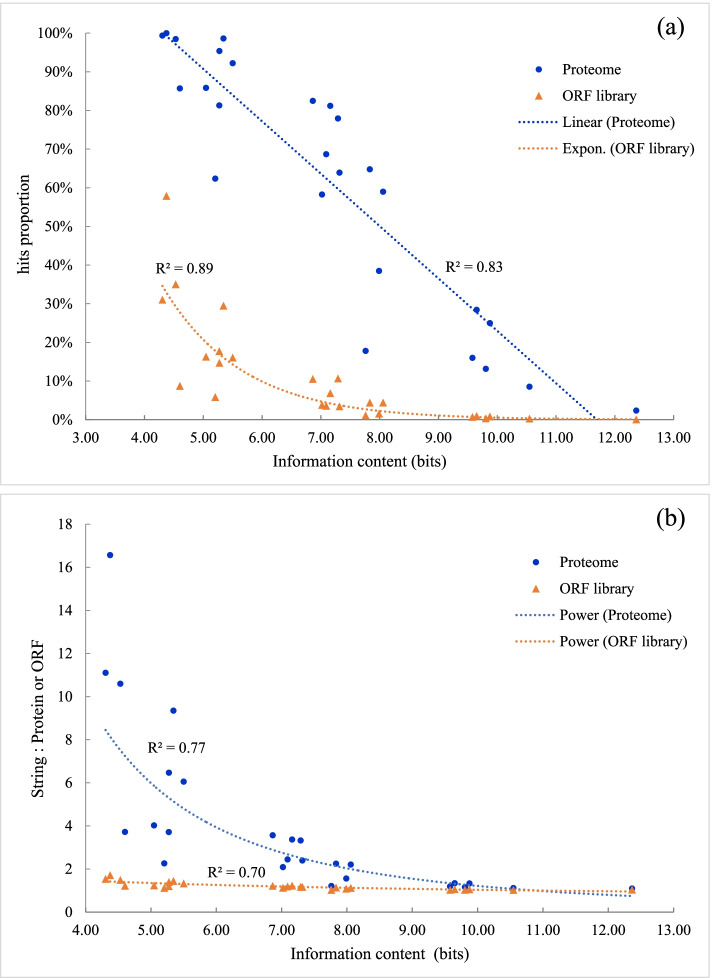
**a** Correlation between profile information content and the proportion of protein hits containing strings matching the profile in the predicted proteome and virtual ORF library. **b** Correlation between profile information content and the ratio of matching strings to protein or ORF hits. Regression model of each data series is shown in the legend and the R^2^ value is marked next to the regression line

A Python script was written and executed to search both the predicted proteome and the virtual ORF library for strings matching regular expressions corresponding to the 26 profiles. Search results are summarized in Table S1. The 26 profiles are characterized by their information contents that quantify the uncertainty of each site in the profile. Regression analysis was performed to model the scattered data series and correlate the profile to the database. As the information content of the profile increases, the proportion of hits (proteins or ORFs) containing string matches decreases linearly (R^2^ = 0.83) in the proteome and exponentially (R^2^ = 0.70) in the ORF library (Fig. 7a). Both databases exhibit different patterns of trends due to the drastic difference in database size and different numbers of hits in each profile. When the information content exceeds 9, the proportions of hits decrease significantly in both data series. There are 6 profiles with information contents higher than 9 and at least 4 of the 5 sites specified. We thus consider that these 6 profiles reasonably characterize putative LLDs and will further investigate the mining results from these 6 profiles. The total number of hits that contain strings matching these 6 profiles in the ORF library is around twice that in the proteome (Table S1). This suggests there should be a considerable number of proteins like G11-514P unidentified by the gene-calling algorithm. It is worth noting the virtual ORF library can only provide an estimate of such proteins potentially encoded in the genome; it is not reliable at the sequence level per se.

We also look at the ratio of matching strings to the hits in relation to the information content of profile (Fig. 7b). Both databases exhibit a downward trend best modelled by power functions towards the increase of profile information content, although the R^2^ values are lower than 70%, meaning that the data series are highly dispersed and thus modestly fitting the regression model. Interestingly, while the proteome has an average ratio of 4.06 ± 3.85, ratios in the ORF library remain constant across all profiles, with a mean of 1.21 and standard deviation of 0.18 (Table S1). In other words, every ORF hit contains about 1 string match. This may be explained by the fact that the virtual ORFs are simply fragments starting with a start codon and terminated when encountering a stop codon, without detecting the introns and joining the exons. Nonetheless, both the proteome and the ORF library have their ratios gradually merge in the 6 profiles with information contents being higher than 9. We interpret this as a sign of consensus sequences in the hits of these 6 profiles. These hits appear to contain few introns, i.e., the protein is encoded by a single exon. The lack of intron is commonly found in the 5 linusorb precursor proteins, with only 1 protein (G14-170 N) containing an intron.

Repeat structure is another feature in most of the linusorb precursor proteins and can be applied to filter the primary candidate pool. If a protein contains only one profile-matching string in its sequence, most likely it is a random match and unrelated to the linusorb precursor proteins. Therefore, a Python script was written to extract protein hits with multiple repeats (≥3) of the matching strings. The extracted proteins containing no fewer than 3 matches were parsed by repeat finder RADAR to determine if the matching strings exhibit repeat patterns. As a result, in addition to sequences that matched the 4 known repeat-containing linusorb precursor proteins, 25 new proteins were detected to contain a total of 281 profile-matching repeats and thus were identified as LLDR proteins. For easier tracking the locations of LLDR encoding genes in the genome, the 25 LLDR proteins were denoted the format “Lu (chromosome number)-(accession number of predicted gene)”, e.g. Lu1–17,106. Names of the 4 linusorb precursor proteins remain unchanged for consistency with published literature. Among them only 10 were identified by the previous genome annotation (Table 2). These differences are not surprising given the complexity of confirming repetitive genomic structures using DNA sequencing, assembly and annotation methods, and warrant further study using both biochemical and Sanger sequencing methods.

**Table 2:** LLDR proteins found or not found in annotation v1.0

LLDR protein	Matched accession in annotation v1.0^a^
Lu1–17,016	Lus10029186
Lu1–18,055	Lus10005187
Lu1–18,070	no
Lu2–51,734	Lus10014515
Lu3–56,299	no
Lu5–45,630	no
Lu5–46,938	no
Lu5–47,766	Lus10028304
Lu6–41,637	Lus10015218
Lu8–2811	no
Lu8–3343	Lus10002240
Lu8–3470	Lus10007009
Lu9–15,288	no
Lu10–34,966	Lus10031895
Lu10–38,024	no
Lu10–38,063	no
Lu11–24,918	no
Lu11–26,217	no
Lu11–28,070	no
Lu12–9873	no
Lu12–11,698	Lus10031461
Lu12–11,761	no
Lu13–23,576	Lus10032171
Lu14–4819	no
Lu14–5765	no

^a^The v1.0 annotation is accessed via *Linum usitatissimum* genome v1.0 on Phytozome (ID: 200) at https://phytozome-next.jgi.doe.gov/info/Lusitatissimum_v1_0

**Fig. 8:**
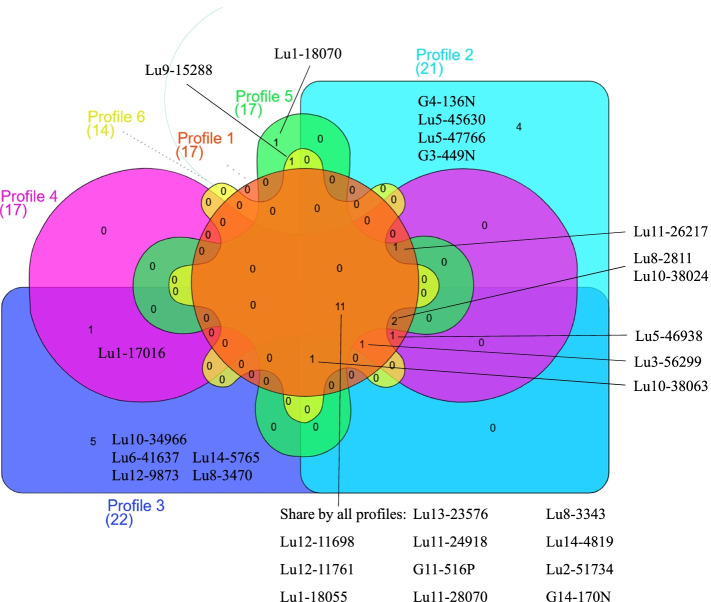
Venn diagram displaying 25 LLDR proteins and 4 linusorb precursor proteins identified to contain repetitive motifs matching 6 different profiles. The number of proteins in each field is indicated. Overlapping fields represent proteins shared by more than one profile. The diagram was created by the online tool InteractiVenn

Figure 8 is a Venn diagram depicting the 25 LLDR proteins together with 4 linusorb precursor proteins categorized by their profile types (information content > 9, Fig. 7). Note that these proteins are identified based on profile matching and motif repetition. There are 17 proteins matching Profile 1 in which all 5 flanking sites are conserved with linusorb-embedded repeats. Except Profile 1, the remaining 5 profiles differ from one another by one site. Nevertheless, it is beyond our expectation that 11 proteins are commonly shared by all 6 profiles, accounting for 50–79% (63% on average) of the collection in each profile. Profiles 2–6 overlap 76–94% (87% on average) of the 11 proteins matching Profile 1. Such major overlaps suggest that the 5 conserved flanking sites are either shared by common ancestry or under functional selection, for example, essential for recognition by modification enzymes involved in the processing of the precursor protein which could lead to convergence with respect to selection for particular polymorphisms.

### The 5 flanking signature sites play a dominant role in potential homology

The 8 proteins retrieved by HMMER (Data S4) are among the 25 LLDR proteins (Fig. 8), which indicates that repeats in these 8 proteins are not only potentially homologous to linusorb-embedded repeats but are also highly conserved in most of the 5 flanking signatures. Among the 8 proteins, 6 match Profile 1 with all 5 flanking signatures conserved. The other 2 proteins, Lu10–34,966 which matches Profile 3 only and Lu9–15,288 which matches Profiles 5 and 6, have 4 conserved flanking signatures. Nevertheless, these 2 proteins contain 26 and 16 repeats, respectively, most of which have the 5 flanking signatures conserved and thus are potential homologues of the linusorb-embedded repeats. The remaining few are the ones matching 4 flanking signatures and thus do not meet the criterion for significant sequence homology.

As exemplified above, not all repeats in the 8 proteins retrieved by HMMER are potential homologues of the linusorb-embedded repeats, and some do not meet the E-value threshold. We thus classified the 281 profile-matching repeats into two categories: 58 potential homologues and 223 non-homologues of the linusorb-embedded repeats. Comparing these two categories reveals which sites contribute to the significance of potential homology by the algorithm in HMMER. The sequence logos of 58 potential homologues in Fig. 5b and 223 non-homologues in Fig. 5c are similar, with some common features including two dominant residues D (10th and 12th positions in Fig. 5b and c, respectively) and F (23rd and 27th positions in Fig. 5b and c, respectively). However, the absolutely conserved G (24th position in Fig. 5b) in the potential homologues are equally dominant with A in the non-homologues (28th position in Fig. 5c).

**Fig. 9:**
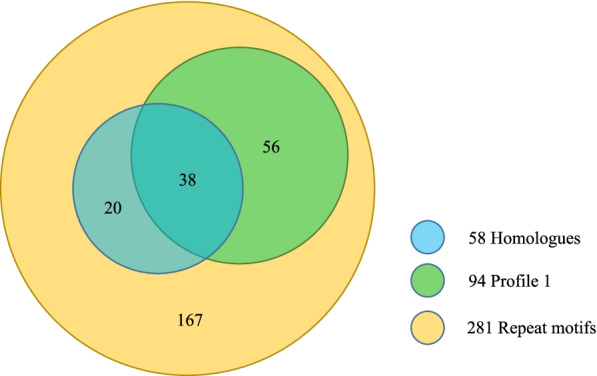
Venn diagram displaying different categories of repeat motifs. Total numbers of repeat motifs under different categories are shown in the legends, and numbers in the diagram represent the numbers of repeat motifs inside each isolated area

Although in non-homologues the 5 signature flanking sites are dominated by the same residues as homologues and linusorb-embedded motifs (Fig. 5a), note that combinations of residues matter. In particular, the homology may not be significant if one site is conserved but other sites vary. This is supported by the percentages of repeats matching Profile 1 in the total number of repeats in the protein matches from different profiles, because only in Profile 1 are all 5 flanking signatures conserved while the other 5 profiles vary in one site. Filtering the 281 repeats with Profile 1 criteria reveals 94 repeats matching Profile 1, among which there are 38 significant homologues (Fig. 9). On one hand, the fact that the profile-matching set overlaps 65.5% of the 58 potential homologues suggests that even though all the 5 flanking signature sites are conserved, other sites contribute significantly to the detected homology. On the other hand, the remaining 20 potential homologues do not match Profile 1 but all match other 5 profiles, indicating that significant homology requires conservation in at least 4 of the 5 flanking signature sites.

The above comparison supports the dominant role of the 5 signature flanking sites in the significance of homology. Meanwhile, as indicated above, other sites in the repeat also contribute. Proline consistently dominates both the linusorb domains (15th position in Fig. 5a) and the LLDs (19th position in Fig. 5b), but such dominant P is missing in the non-homologues (Fig. 5c). It is therefore reasonable to associate this P with the homology of linusorb-embedded repeats. There should be more other sites with considerable weights in the profile HMM but less visibly evident in the sequence logo.

### Potential correlation between the leader peptide region and the core peptide region

The leader peptide located N-terminal to the core peptide region is an important part for recognition by the post-translational modification enzymes and export of the modified precursor protein [2]. It contains a signal sequence at the N-terminus that directs the precursor protein to a specific cellular compartment for post-translational modifications. Therefore, the leader peptides of the 5 known linusorb precursor proteins may share some conserved patterns in their sequences that are useful for proteome mining to identify proteins with similar leader sequences. A profile HMM was built from the alignment of leader peptides of the 5 linusorb precursor proteins and used as a query to search the predicted proteome (Data S5). The first round retrieved 1 significant match beyond the original leader peptides. This hit, Lu2–51,734 (proteome accession: g51734), was among the 25 LLDR proteins identified above. The N-terminal region of Lu2–51,734 matches the input profile HMM, with many residues aligned consecutively as marked by the asterisks and scores higher than 5 in the match alignment. This supports that the N-terminal region of Lu2–51,734 is homologous to the leader peptides of the linusorb precursor proteins and may also act as a leader peptide. The second round of HMM search using the updated profile HMM built from the alignment of the N-terminal region of Lu2–51,734 and the original leader peptides retrieved no additional matches and thus terminated the HMM search.

There are 4 hits with larger E-values, among which Lu1–18,055 (g18055) and Lu3–56,299 (g56299) are members of the 25 LLDR proteins. The other 2 hits, g39692 and g11605, were analysed by RADAR for the presence of any repeats. Only g39692 contains repeats, and importantly, the 2 repeats match our customized profile: DGAVILVNIFGK and DATMWGGVDSFGK (conserved flanking signatures underlined). This protein was neglected in the profile-based proteome mining because it contains fewer than 3 repeats. Nonetheless, the fact that proteome mining based on leader peptide homology found potential homologues of repeats in the core peptide region suggests strong correlation between the leader peptide and the core peptide region in these LLDR proteins. Conservation of both the leader peptide and flanking signatures further suggests that these LLDR proteins may undergo similar post-translational modification to the linusorb precursor proteins.

## Discussion

### Goal of mining: genetic diversity or chemical diversity?

Since the advent of next-generation sequencing, omics database mining has been driving new approaches to the discovery of natural products, especially RiPPs because of their direct link between the gene sequences and the peptide products. However, the diversity of RiPPs poses a challenge for mining to predict RiPPs-encoding sequences. Previous mining practices have largely focused on identifying potential homologues of cyclic peptide precursor proteins in closely related species. Numerous peptides have been identified by MS analysis, but a larger majority of mining candidates have not been validated through chemical identification of products. Little attention has been paid to candidates identified by omics with no corresponding product or the relationship between these sequences and the detected ones. Most mining candidates have been retrieved by employing search tools like BLAST based on their high similarity with a known protein sequence, and thus contain genetic information related to the biosynthetic mechanisms and evolution of cyclic peptides. Here we employed bioinformatics tools to explore the genetic diversity of linusorbs, the flaxseed orbitides. Each search tool identified patterns from different regions of the linusorb precursor proteins, which resulted in the detection of novel linusorb-like sequences. The collection of linusorb-like sequences provides a diverse pool for subsequent studies on the evolutionary relationship to the known linusorb sequences and expands the candidate pool of potential linusorbs that can be synthesized in engineered organisms for practical applications.

Theoretically, genetic diversity and chemical diversity of RiPPs are related to each other in that every RiPP a plant produces is encoded by a gene sequence; however, the link between the gene and the peptide has proven to be weak. Several factors, both biological and technical, limit the diversity of peptides that can be identified chemically. From the biological perspective, peptide biosynthesis involves expression of a protein as a precursor for post-translational modification by proteolytic enzymes. The production of peptides depends on regulation of the precursor protein expression (i.e., how much, when and where), the activity of modification enzymes and many other factors associated with downstream metabolic pathways. Consequently, only those minimally modified peptides resemble the primary sequences the most and are readily detectable, representing a subset of all possible peptide sequences, as the discovery of peptides and their variants largely relies on prior knowledge of their physicochemical properties.

To achieve unambiguous identification, analytical processes which involve extraction of crude peptides, separation by liquid chromatography (LC) and determination by mass spectrometry (MS) need to be customized for a specific class of peptides based on their molecular weight, polarity, linkage moiety and other structural information, most of which is obtained from empirical studies of known peptides. This is a bottleneck for identifying novel peptides with unknown modifications which might require special treatment for successful analysis. Undetected peptides might share the same precursor proteins or their homologues with the detected peptides, underlying useful clues about cyclic peptide evolution. Elucidating biological mechanisms solely from those chemically validated peptides may result in ascertainment bias. Therefore, while chemical diversity based on product validation provides solid evidence, the conserved patterns from the known peptide sequences are more important for exploring a greater diversity of genetically relevant sequences to reveal a bigger picture at the omics level.

### Profile-based mining is suitable for exploring the genetic diversity of orbitide-related sequences

The currently identified > 20 linusorb structural variants are derived from 11 linusorb domains in 5 precursor proteins [3]. The distribution of linusorb domains is highly variable, in that some domains have multiple copies in the precursor protein and some domains are found in two precursor proteins. Such promiscuity and diversity necessitate investigations of relationships among the known linusorb domains as well as their precursor proteins. Linusorb domains within the same precursor proteins are more closely related than to those from other precursor proteins. The 5 linusorb precursor proteins are clustered into two clades and a singleton (Fig. 3b), which indicates that they might have originated from 3 different ancestors. This accounts for the BLAST outputs that using the whole precursor protein as query could only identify potential homologues within the same clade but not members from the other clade. The relationship among the 5 linusorb precursor proteins accentuates the fact that using the whole precursor protein as query restricts the mining to peptide variants from the potential protein homologues rather than from more distantly related proteins. Many reported mining practices largely employed this approach for the discovery of novel peptides. For instance, Fisher et al. (2020) used PamAD, the precursor protein of known annomuricatins A and D, as a query for tBLASTn searches, which led to the discovery of five potential annomuricatin precursor proteins with high similarity [32]. All 9 annomuricatin domains in the 5 protein candidates were chemically confirmed to exist as orbitides with variable sequences. Alignment of the 5 annomuricatin precursor proteins showed multiple conserved sites flanking the annomuricatin domains, demonstrating their potential homology. While searching for potential homologues of known annomuricatin precursor proteins secures high validity of the predicted annomuricatins, the novel precursor proteins are highly similar in sequence, length and even the number of annomuricatin domains. This may exclude the possibility of finding more divergent annomuricatin or annomuricatin-like precursor proteins.

The limitation of sequence similarity searches for local sequence alignment with the whole precursor protein query calls for a more specific mining strategy. Repeat detection and analysis of the core peptide region with respect to multiple orbitide-embedded repeats provides an informative approach for resolving the relationships of the 5 linusorb precursor proteins. Alignment of repeats revealed the hypervariability of linusorb domains flanked by several highly conserved sites. Such a uniform pattern of linusorb-embedded repeat units across different precursor proteins has fostered a profile-based mining strategy. We compared 3 search methods that employ different algorithms of profile recognition to perform proteome mining for profile-matching sequences. While different methods retrieved different matches, all linusorb precursor proteins present in the predicted proteome were recovered by all 3 methods, highlighting the power of a profile-based mining strategy to identify distantly related proteins. Among the 2 algorithm-based search methods, HMMER extracted 8 new proteins in which 58 repeats significantly matched the profile HMM built from linusorb-embedded repeats, while PSI-BLAST found only 2 of the 8 proteins as matches with large E-values. The recovered sequences contrasted due to differences in profile construction and match scoring between both tools. Although non-significant matches do not meet the significance threshold, many of them contain sites matching the conserved sites in the input alignment. Additionally, using aligned repeats as query achieved higher E-values compared to using the whole protein as query due to the much shorter length than protein. A previous study on the repeat-containing kinetochore protein scaffold KNL1 revealed that classical homology searches using BLAST failed to detect sufficient homology for KNL1 genes, and demonstrated the power of iterative homology search using HMMER that found 110 diverged homologues in a variety of eukaryotic species [42]. The KNL1 protein homologues contain variable numbers of repeats, each of which is composed of a conserved MELT-like motif flanked by variable sequences. This repeat structure is not consistent with our linusorb-embedded repeats where the linusorb domain is variable and flanked by conserved residues. In essence, they share the same characteristic that a mixture of conserved and variable sites is unevenly distributed within a varying number of repeats among homologues.

Both PSI-BLAST and HMMER build profiles from the alignment of linusorb-embedded repeats, meaning that any conserved sites in the linusorb domain will contribute to the profile. To maximize the variability of the linusorb domain, we developed customized profiles using the 5 most conserved sites flanking the linusorb domain represented by 4–13 random residues and wrote Python scripts to search the proteome for strings matching the profile. Screening the primary matches for those containing multiple repeats identified 25 LLDR proteins with 281 profile-matching repeats. The 8 proteins with 58 potential homologues of the linusorb-embedded repeats are a subset of the total collection. This profile-based string search method is one step further from HMMER by extracting a wider range of repeats that are distantly related to the linusorb-embedded repeats. The randomization of the linusorb domain region expands the collection of orbitides flax can potentially produce, from which we can gain more insights into the genetic relationship among these sequences.

Some of the published mining practices also appear to be profile-based, especially for cyclotides, for which the structures have been well studied. Park *et. al.* (2017) employed two search tools, BLAST and PROSITE, to search the transcriptomes of various *Viola* species using known cyclotide sequences as queries [20]. Matches were aligned and manually screened for sequences with 6 conserved cysteines aligned to the known cyclotides. Compared to our string search method using a pre-defined profile, this mining approach utilized the profile in the opposite way. The searches were based on the similarity to known cyclotide sequences, rather than to a profile built from the alignment of multiple sequences. Although the “profile”, i.e., 6 conserved cysteines aligned to the known positions, was applied to screen the initial matches, the screening was done from the potential homologues of known cyclotides extracted by both search tools. As their goal was to explore cyclotide evolution in the *Viola* genus, this mining process could extract sequences like the known cyclotides in other *Viola* species, enabling the reconstruction of the evolutionary relationships of orthologues among different species at the cyclotide level. However, this approach cannot identify cyclotide-like domains with low similarity to known cyclotides but anchored by the 6 conserved cysteines. This contradicts their assumption that the sequence is a cyclotide precursor if the sequence contains the 6 conserved cysteines aligned with previously known cyclotides. Therefore, it is imperative to identify a specific goal and design a cohesive mining scheme towards the goal.

### Repetitive elements should be treated with caution in the study of repeat-containing proteins

One of the structural features shared by many cyclic peptide precursor proteins is the repeats that constitute the core peptide region. These repeats have high sequence similarity and thus are identified as low-complexity sequences like those non-coding repeats. As it is a routine treatment to mask the repetitive elements in many genome assembly practices [43], a considerable number of repeat-containing genes are filtered out, as is the case for all the linusorb precursor proteins. The flax genome sequencing project identified 73.8 Mb repetitive sequences using both homology-based methods and de novo identification methods, accounting for 24.4% of a total of 302 Mb WGS contigs [23]. These repeats were masked by RepeatMasker before the genome was annotated. As a result, the proteome generated from the repeat-masked genome assembly was devoid of linusorb precursor proteins we previously identified. This poses a barrier not only for novel orbitide discovery but also for exploring orbitide-related proteins diversity. Therefore, to achieve a more accurate identification of the LLDR proteins, we retained the repetitive sequences in the genome assembly and predicted genes from the genome by integrating RNA-Seq reads aligned to the genome as experimental evidence of flax-specific gene models. The predicted proteome contains all the 4 known linusorb precursor proteins with repetitive structure in the core peptide region, and thus serves as a high-quality database for mining.

Another treatment specific for repeats is to dissect them into individual repeat units and align them across different precursor proteins, due to their varying copy number and length from protein to protein. However, few studies have performed this treatment before their mining experiments. The potential relationships among the repeats could not be resolved until they were identified by the repeat finder RADAR and properly aligned. In terms of mining, using the entire protein as query failed to differentiate the hypervariable linusorb domain from the conserved flanking sites. The short length and discrete conserved sites interspersed by variable lengths of hypervariable domains hinder BLAST from accurately finding homologues with different numbers of repeat units [42, 44]. Therefore, it is necessary to separate each repeat and align them at the conserved flanking sites for higher accuracy. Our profile-based mining methods extracted hypervariable repeats including those potentially homologous and those distantly related to the known linusorb-embedded repeats, demonstrating the genetic diversity of linusorb-related sequences at different levels.

### Conserved flanking sites: common ancestry or convergent evolution?

Our profile-based mining approach identified 25 LLDR proteins that contain 281 LLD repeats. These repeats share a structural profile with the linusorb-embedded repeats where a variable domain is flanked by 5 conserved sites. From an evolutionary perspective, it would be interesting to know whether these conserved sites originate from shared ancestry or are resulted from convergent evolution (i.e., representing a functional constraint). The HMM search result has shed light on this question. Among the 281 LLD repeats, 58 exhibit significant sequence homology to the linusorb-embedded repeats, implying that they share common ancestry. However, the 8 LLDR proteins containing these 58 LLD repeats are unrelated to the 5 linusorb precursor proteins, as tBLASTn found no additional matches in the genome with similarity comparable to the linusorb precursor proteins to themselves (Data S1). While the 58 LLD repeats were determined to be significant homologues in the HMM search, it is unclear how they became linked to the 8 LLDR proteins, and the lack of homology between the 5 linusorb precursor proteins and the 8 LLDR proteins potentially points to selection for particular polymorphisms under functional constraint (e.g., splicing recognition).

The remaining 223 LLD repeats do not meet the significance threshold and thus are categorized as non-homologues. Only the 5 signature sites are conserved in these non-homologues, flanking variable domains that are unrelated to the linusorb-embedded repeats. This additionally suggests that conservation of the 5 flanking signature sites in these 223 LLD repeats has resulted from convergent evolution for functional polymorphisms, e.g., playing a role in the recognition by proteolytic enzymes to cleave the linusorb precursor protein. In 25 LLDR proteins with no linusorbs identified, these sites may be also recognized by the same family of protease, but the particular protease homologue may function differently from catalyzing linusorb synthesis. Proteases have been shown to exhibit various functions in almost all aspects of plant life, including development, homeostasis, stress response and defense [45]. For example, the subtilisin-like proteases (subtilases) are serine proteases that act on multiple substrates to regulate plant-pathogen interaction and immune response [46]. It is therefore reasonable to infer that the conserved flanking sites in the 223 LLD repeats are functionally selected along with the protease activity. More functional evidence of these proteins is required to clearly unravel the adaptive convergence process.

## Conclusions

For natural products, genetic diversity underlies chemical diversity. Besides genetic factors, biosynthesis of natural products in plants is regulated by environmental conditions. Chemistry-directed research on natural products only reflects the surface of this profound network. Therefore, the present work explored the genetic diversity of linusorbs in flax by bridging the gap between the sequenced genome and our previously isolated linusorbs. We showed that conventional homology search by BLAST using the whole protein sequence as query is limited to finding potential protein homologues. Switching the mining strategy from protein-based to profile-based has helped overcome the challenge of repeat number variation. Querying the proteome with profiles built from the alignment of linusorb-embedded repeats could retrieve all linusorb precursor proteins by both PSI-BLAST and HMMER, and HMMER worked better at searching for more distant homologues of the linusorb-embedded repeats. The profile was further simplified to 5 conserved sites flanking the randomized linusorb-like sequences. Repeats matching this customized profile represent the most diverse collection of linusorb-like sequences genetically related to the known linusorbs. How these sequences are related to one another will be the next question to answer, and determining their relationships will be the key to unraveling the evolutionary mechanism underlying their diversity.

## Methods

### Sequence alignment and phylogenetic analysis

Sequence analyses were conducted using 11 linusorb domains and 5 known linusorb precursor proteins to investigate their relationships [3]. Multiple sequence alignments were generated by MUSCLE 3.8.31 [47, 48] using the following command:

- $ muscle -in 5-precursors.fasta -fastaout 5-precursors_B62g10e1-clusternj-dist1kmer20_3.aln -cluster neighborjoining -distance1 kmer20_3 -matrix BLOSUM62 -gapopen -10 -gapextend -1 -verbose -log 5-precursors_B62g10e1-clusternj-dist1kmer20_3.log

From the alignment a neighbor-joining (NJ) tree was constructed with 1000 bootstrap replications using MEGA7 [49]. Midpoint rooting was applied to the NJ tree.

### BLAST search for potential protein homologues

The 5 known linusorb precursor proteins were used as queries to search the genome assembly of *L. usitatissimum* cv. CDC Bethune (GenBank accession: GCA_000224295.2) with tBLASTn. The substitution matrix was BLOSUM62. The word size was set to 2 and the expect value (E-value) threshold was 10 by default. Low-complexity filtration was disabled to ensure the whole protein sequence was queried.

### Repeat-retaining genome re-annotation

The genome annotation conducted by Wang et al. [23] removed repetitive elements from the genome assembly using RepeatMasker. Thus, the resulting proteome lacks the 5 known linusorb precursor proteins as positive controls, which suggests that this proteome is not a suitable reference for predicting linusorb precursor protein analogues. We thus re-annotated the genome assembly of Wang et al. [23] without masking repeats as follows.

The eukaryotic genome annotation pipeline, BRAKER2 [50], was utilized to annotate the unmasked genome assembly. First, RNA-Seq reads produced by [51] were downloaded from NCBI and aligned to the CDC Bethune genome by HISAT2 [52]. Using hints from the alignments, the self-training algorithm GeneMark-ET [53] identified gene models by directing a semi-unsupervised training. The gene prediction program AUGUSTUS [54] also generated gene models from the alignments and corroborated its gene models through training by the gene models from GeneMark-ET. Finally, with the support of combined gene models, AUGUSTUS predicted genes and alternative transcripts from the genome. BRAKER2 output the predicted gene calls and corresponding proteome.

### Homology search using PSI-BLAST

An iterative search was performed with the tool PSI-BLAST to mine the proteome generated from genome reannotation for potential homologues of the linusorb-embedded repeats. First, a position specific scoring matrix (PSSM) was generated from the multiple alignment of linusorb-embedded repeats in the original 5 precursor proteins. Then a PSI-BLAST search was conducted to identify sequences matching the PSSM input. Matches with E-values below 0.01 were accepted and combined with the original alignment for the next round of search until no new matches were identified.

### Homology search based on hidden Markov model (HMM)

Proteins containing repeat sequences that are potential homologues of the linusorb-embedded repeats were identified based on the profile HMM match. First, a profile HMM was built from the alignment of original linusorb-embedded repeats using the *hmmbuild* tool with default parameters in the HMMER suite [29]. Next, *hmmsearch* was conducted to search the BRAKER2-predicted proteome with the input profile HMM. Statistically significant protein matches were taken above the default inclusion threshold (10.0, HMMER User’s Guide, Version 3.2.1). Hits marked with “!” were determined as significant by HMMER, and the subject segments of these hits were considered as “true” homologues. These homologous subject segments were then extracted from the output file and aligned to the original alignment using *hmmalign* based on the input profile. The combined alignment was used to build a new profile HMM for the next round of search. Such searching process was iterated until no additional proteins were identified that met the inclusion threshold.

### Proteome mining for strings matching customized profiles

Based on conserved sites in the alignment of linusorb-embedded repeats, sequence profiles were created in the form of regular expressions that can be interpreted by Python, whereby a script was developed to automate proteome mining. Accessions containing sequence strings that matched the specified regular expression were extracted to a primary candidate pool. The primary candidate protein pool was refined using a Python script to enumerate candidate protein matches and screen for those with multiple (≥ 3) matches. The refined candidate pool was then filtered by detecting matching strings that exhibit repeat pattern using RADAR [55]. Finally, proteins harboring multiple repeats were classified as proteins with Linusorb-Like-Domains-containing-Repeats (LLDR proteins). Prior to mining, the Python scripts were validated for the ability to recover positive controls, i.e., the known precursor proteins from which the profiles were developed.

### Visualization of repeats

WebLogo3 was employed to create sequence logos from the multiple sequence alignments of the following 3 datasets [56]: (a) the linusorb-embedded repeats identified by RADAR; (b) significant homologues of the linusorb-embedded repeats retrieved from PSI-BLAST and HMM search; and (c) repeats matching the customized profile retrieved from the string search excluding the “true” homologues in (b). Sequences of each dataset were aligned by MUSCLE and each alignment was submitted to WebLogo3 for logo construction.

## Supplementary Information

**Additional file 1: Fig. S1.** Phylogenetic trees of (a) 11 linusorb (LO) domains and (b) 5 linusorb precursor proteins constructed with the Maximum Likelihood (ML) method.

**Additional file 2: Data S1.** BLAST search for potential homologues of 5 precursor proteins.

**Additional file 3: Data S2.** RADAR-detected repetitive motifs in linusorb precursor proteins.

**Additional file 4: Data S3.** Repeat PSI-BLAST output.

**Additional file 5: Data S4.** Repeat HMM search output.

**Additional file 6: Data S5.** Leader peptide HMM search output

**Additional file 7: Table S1.** Statistics of 26 profiles

## Data Availability

The datasets generated and analyzed during the current study are available in the DDBJ repository, accessions BR001772 – BR001801.
