# Supplementary material for: The flax genome reveals orbitide diversity
Source: BMC Genomics. 2022 Jul 23;23:534. doi: 10.1186/s12864-022-08735-x (PMC9308333; doi:10.1186/s12864-022-08735-x)
Supplement: Supplementary file 1 — Additional file 1: Fig. S1. Phylogenetic trees of (a) 11 linusorb (LO) domains and (b) 5 linusorb precursor proteins constructed with the Maximum Likelihood (ML) method. [file 12864_2022_8735_MOESM1_ESM.docx]

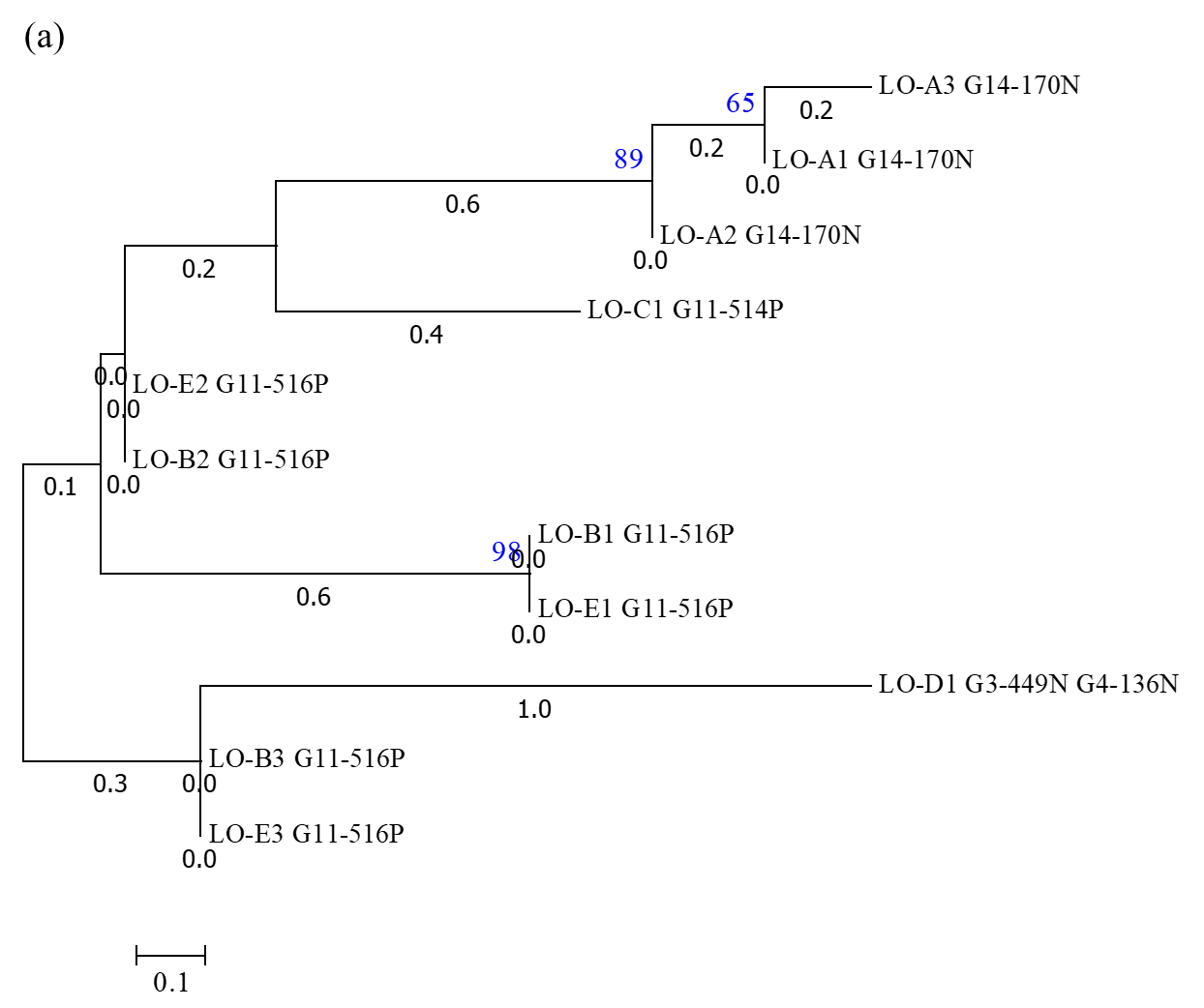


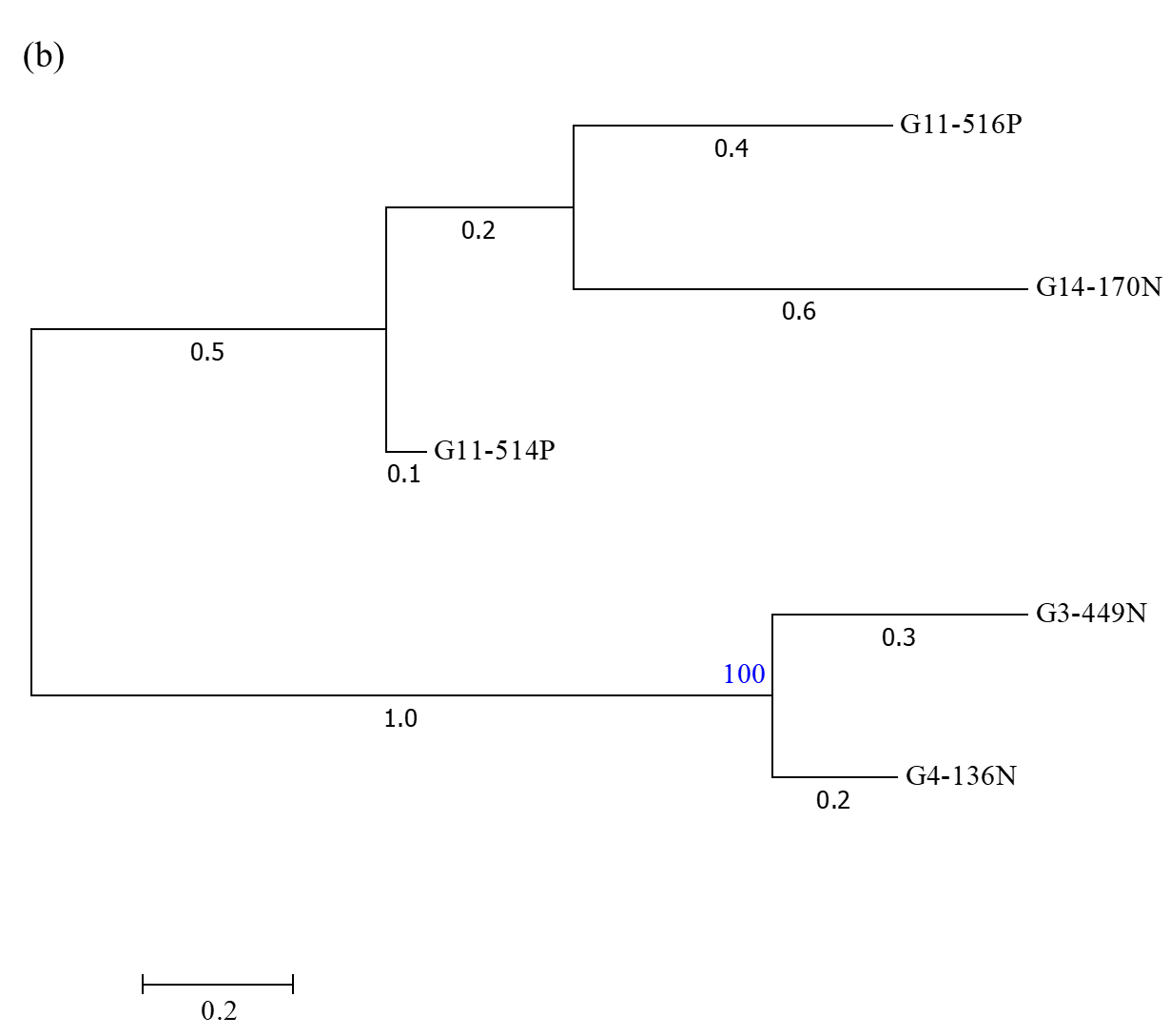


Figure S1. Phylogenetic trees of (a) 11 linusorb (LO) domains and (b) 5 linusorb precursor proteins constructed with the Maximum Likelihood (ML) method. Sequences were aligned by MUSCLE. Numbers in blue above the nodes represent the bootstrap values of 1000 replications. Only nodes with bootstrap values ≥ 60 are considered significant and have their bootstrap values displayed. Numbers in black below the nodes represent the branch lengths, i.e. genetic distance between two nodes.
