# Supplementary material for: The flax genome reveals orbitide diversity
Source: BMC Genomics. 2022 Jul 23;23:534. doi: 10.1186/s12864-022-08735-x (PMC9308333; doi:10.1186/s12864-022-08735-x)
Supplement: Supplementary file 2 — Additional file 2: Data S1. BLAST search for potential homologues of 5 precursor proteins. [file 12864_2022_8735_MOESM2_ESM.docx]

To search for potential homologues of the 5 known linusorb precursor proteins, tBLASTn was performed to use the whole precursor protein sequence as query to interrogate the *L. usitatissimum* cv. CDC Bethune genome assembly (GenBank accession: GCA_000224295.2). Given the lengths of 4 precursor proteins longer than 85 residues and G11-514P being 76 residues, BLOSUM62 and BLOSUM80 was used for the other 4 proteins and G11-514P, respectively, as recommended by the BLAST manual. The word size was set to 2 and the E-value threshold was 10 by default. Low complexity regions were not filtered for a full coverage of the query sequence. Potential homologues were determined by both the absolute E-values and the relative differences between two E-values. An E-value of 1e^-10^ is a typical threshold for most protein sequences with lengths not shorter than 100 aa. However, for those shorter than 100 aa which generate E-values higher than 1e^-10^, or multiple hits with E-values lower than 1e^-10^, relative differences between two adjacent E-values should be taken into account. For hits sorted by ascending E-values, a drastic increase in the E-value difference between two adjacent hits relative to those in the upper hits indicates the loss of significance. The upper hits above such E-value gap are thus considered significant.

# >G14-170N Linusorb A1-A3 precursor protein

MAAASSLALATASLVATGAGGRNNAFLPSKNKTPNLFLNPNKTTSSTVKAVVSSSSCKRPYPKGDASLFLGIDDVFGKDAVAGHDNDQDAASGQEMAADDMLMPFFWIFGKEGQQQEAEESSDDMLMPFFWIFGKEGQQQEAESSDDMLLPFFWIFGKEGQQQEAESSDDMLMPFFWIFGKQQQQQGESSDDMLMPFFWVFGKQGDNNKGDAVEAILKN


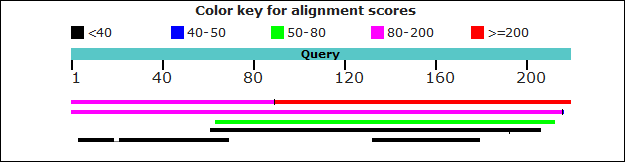


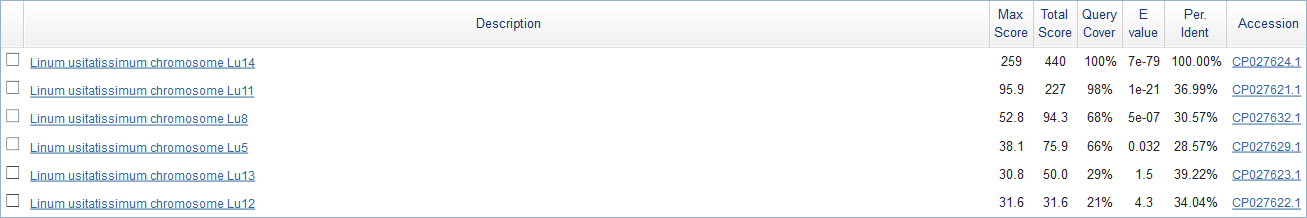


The 2nd hit is taken as a potential homologue.

First hit: the query itself, two ranges of alignment are separated by a gap of ~971bp which is an intron.

Linum usitatissimum chromosome Lu14

Sequence ID: [CP027624.1](https://www.ncbi.nlm.nih.gov/nucleotide/CP027624.1?report=genbank&log$=nuclalign&blast_rank=1&RID=B0DYBR30016) Length: 19392306 Number of Matches: 2

Range 1: 17099156 to 17099545

| Alignment statistics for match #1 | | | | | | |
| --- | --- | --- | --- | --- | --- | --- |
| **Score** | **Expect** | **Method** | **Identities** | **Positives** | **Gaps** | **Frame** |
| 259 bits(663) | 7e-79 | Compositional matrix adjust. | 130/130(100%) | 130/130(100%) | 0/130(0%) | -3 |

Query 90 AASGQEMAADDMLMPFFWIFGKEGQQQEAEESSDDMLMPFFWIFGKEGQQQEAESSDDML 149

AASGQEMAADDMLMPFFWIFGKEGQQQEAEESSDDMLMPFFWIFGKEGQQQEAESSDDML

Sbjct 17099545 AASGQEMAADDMLMPFFWIFGKEGQQQEAEESSDDMLMPFFWIFGKEGQQQEAESSDDML 17099366

Query 150 LPFFWIFGKEGQQQEAESSDDMLMPFFWIFGKQQQQQGESSDDMLMPFFWVFGKQGDNNK 209

LPFFWIFGKEGQQQEAESSDDMLMPFFWIFGKQQQQQGESSDDMLMPFFWVFGKQGDNNK

Sbjct 17099365 LPFFWIFGKEGQQQEAESSDDMLMPFFWIFGKQQQQQGESSDDMLMPFFWVFGKQGDNNK 17099186

Query 210 GDAVEAILKN 219

GDAVEAILKN

Sbjct 17099185 GDAVEAILKN 17099156

Range 2: 17100516 to 17100782

| Alignment statistics for match #2 | | | | | | |
| --- | --- | --- | --- | --- | --- | --- |
| **Score** | **Expect** | **Method** | **Identities** | **Positives** | **Gaps** | **Frame** |
| 180 bits(457) | 3e-51 | Compositional matrix adjust. | 89/89(100%) | 89/89(100%) | 0/89(0%) | -2 |

Query 1 MAAASSLALATASLVATGAGGRNNAFLPSKNKTPNLFLNPNKTTSSTVKAVVSSSSCKRP 60

MAAASSLALATASLVATGAGGRNNAFLPSKNKTPNLFLNPNKTTSSTVKAVVSSSSCKRP

Sbjct 17100782 MAAASSLALATASLVATGAGGRNNAFLPSKNKTPNLFLNPNKTTSSTVKAVVSSSSCKRP 17100603

Query 61 YPKGDASLFLGIDDVFGKDAVAGHDNDQD 89

YPKGDASLFLGIDDVFGKDAVAGHDNDQD

Sbjct 17100602 YPKGDASLFLGIDDVFGKDAVAGHDNDQD 17100516

# Potential homologue:

Linum usitatissimum chromosome Lu11

Sequence ID: [CP027621.1](https://www.ncbi.nlm.nih.gov/nucleotide/CP027621.1?report=genbank&log$=nuclalign&blast_rank=2&RID=B0DYBR30016) Length: 19887771 Number of Matches: 4

Range 1: 2516493 to 2517047

| Alignment statistics for match #1 | | | | | | |
| --- | --- | --- | --- | --- | --- | --- |
| **Score** | **Expect** | **Method** | **Identities** | **Positives** | **Gaps** | **Frame** |
| 95.9 bits(237) | 1e-21 | Compositional matrix adjust. | 91/246(37%) | 109/246(44%) | 92/246(37%) | +3 |

Query 1 MAAASSLALATASLVATGAGGRNNAFLPS---KNKTP-NLFLNPNKTTSSTVKAVVSSSS 56

MA SSLAL T SLVAT AG NNAF PS NK P +LF+ P TT TVKA ++ S

Sbjct 2516493 MAVVSSLALTT-SLVATAAGRNNNAFPPSSSRNNKAPADLFITPKTTT--TVKA--AAVS 2516657

Query 57 CKRPYPKGDASLFLGIDDVFGKDAVAGHDNDQDAASGQEMAADDMLMPFFWIFGKEGQQQ 116

CKRPYPKG AVA AA L P

Sbjct 2516658 CKRPYPKG---------------AVA--------------AATSTLSP------------ 2516714

Query 117 EAEESSDDMLMPFFWIFGKEGQQQEAESSDDMLLPFFWIFGKEGQQ---------QEAES 167

I GK+G + E SD ML+ +IFGKEG Q ++ E

Sbjct 2516715 ---------------ISGKDGGLRNQEESDGMLVFPLFIFGKEGSQDKYNGAAALRDQEE 2516849

Query 168 SDDMLM-PFFWIFGKQQQQ-----------------QGESSDDMLMPFFWVFGKQGDNNK 209

SD ML+ PFF IFGK+ Q Q ES ++ PFF +FGK+G +K

Sbjct 2516850 SDGMLIPPFFVIFGKEGCQDIGHKYNNAAAAGALRDQEESDGILVPPFFLIFGKEGSQDK 2517029

Query 210 GDAVEA 215

+A A

Sbjct 2517030 YNAAAA 2517047

This hit corresponds to a known linusorb precursor protein G11-516P containing linusorbs B1-B3.

Alignment of G11-516P and G14-170N by MUSCLE:

G14-170N MAAASSLALATASLVATGAGGRNNAFLPS---KNKTP-NLFLNPNKTTSSTVKAVVSSSS

G11-516P MAVVSSLAL-TTSLVATAAGRNNNAFPPSSSRNNKAPADLFITPKTTTTVKAAAV----S

**..***** *:*****.** .**** ** :**:* :**:.*:.**: .. ** *

G14-170N CKRPYPKGD-ASLFLGIDDVFGKDAVAGHDNDQDAASGQEMAADDMLMPFFWIFGKEGQQ

G11-516P CKRPYPKGAVAAATSTLSPISGKD--GGLRNQEE--------SDGMLVFPLFIFGKEGSQ

******** *: :. : *** .* *::: :*.**: ::******.*

G14-170N QE--------AEESSDDMLM-PFFWIFGKEGQQQEAESSDDMLLPFFWIFGKEGQQQEAE

G11-516P DKYNGAAALRDQEESDGMLIPPFFVIFGKEGCQDIGHKYNNA--------AAAGALRDQE

:: :*.**.**: *** ****** *: . . :: . * .: *

G14-170N SSDDMLM-PFFWIFGKQQQQQGESSDDMLMPFFWVFGKQGDNNKGDAVEAILKN

G11-516P ESDGILVPPFFLIFGKEGSQDKYNAAA-------AGGLRGKEQQGDKMAAGAEN

.**.:*: *** ****: .*: .: . * .*.:::** : * :*

The alignment shows both proteins can align to each other at the whole protein level, but there are some gaps caused by different numbers of linusorb-containing repeats between the two proteins.

# >G11-516P Linusorb B1-B3 precursor protein

MAVVSSLALTTSLVATAAGRNNNAFPPSSSRNNKAPADLFITPKTTTTVKAAAVSCKRPYPKGAVAAATSTLSPISGKDGGLRNQEESDGMLVFPLFIFGKEGSQDKYNGAAALRDQEESDGMLIPPFFVIFGKEGCQDIGHKYNNAAAAGALRDQEESDGILVPPFFLIFGKEGSQDKYNAAAAGGLRGKEQQGDKMAAGAEN


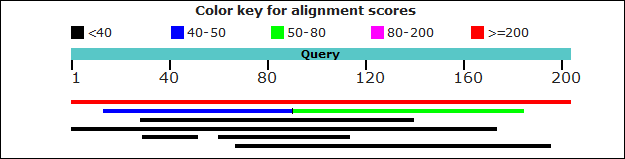


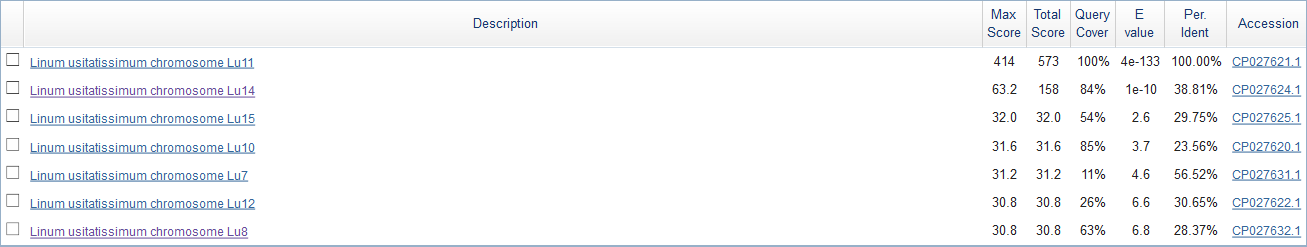


The 2nd hit is taken as potential homologue.

# Potential homologue:

Linum usitatissimum chromosome Lu14

Sequence ID: [CP027624.1](https://www.ncbi.nlm.nih.gov/nucleotide/CP027624.1?report=genbank&log$=nuclalign&blast_rank=2&RID=BB7A4H83016) Length: 19392306 Number of Matches: 3

Range 1: 17099168 to 17099536 [GenBank](https://www.ncbi.nlm.nih.gov/nucleotide/CP027624.1?report=genbank&log$=nuclalign&blast_rank=2&RID=BB7A4H83016&from=17099168&to=17099536) [Graphics](https://www.ncbi.nlm.nih.gov/nuccore/CP027624.1?report=graph&rid=BB7A4H83016%5bCP027624.1%5d&tracks=%5bkey:sequence_track,name:Sequence,display_name:Sequence,id:STD1,category:Sequence,annots:Sequence,ShowLabel:true%5d%5bkey:gene_model_track,CDSProductFeats:false%5d%5bkey:alignment_track,name:other%20alignments,annots:NG%20Alignments|Refseq%20Alignments|Gnomon%20Alignments|Unnamed,shown:false%5d&v=17099150:17099554&appname=ncbiblast&link_loc=fromHSP)

| Alignment statistics for match #1 | | | | | | |
| --- | --- | --- | --- | --- | --- | --- |
| **Score** | **Expect** | **Method** | **Identities** | **Positives** | **Gaps** | **Frame** |
| 63.2 bits(152) | 1e-10 | Compositional matrix adjust. | 52/134(39%) | 68/134(50%) | 22/134(16%) | -3 |

Query 63 GAVAAATSTLSP---ISGKDGGLRNQEESDGMLVFPLF-IFGKEGSQDKYNGAAALRDQE 118

G AA L P I GK+G + EES ++ P F IFGKEG Q ++ E

Sbjct 17099536 GQEMAADDMLMPFFWIFGKEGQQQEAEESSDDMLMPFFWIFGKEGQQ---------QEAE 17099384

Query 119 ESDGMLIPPFFVIFGKEGCQDIGHKYNNA-----AAAGALRDQ--EESDGILVPPFFLIF 171

SD ML+ PFF IFGKEG Q ++ G + Q E SD +L+ PFF +F

Sbjct 17099383 SSDDMLL-PFFWIFGKEGQQQEAESSDDMLMPFFWIFGKQQQQQGESSDDMLM-PFFWVF 17099210

Query 172 GKEGSQDKYNAAAA 185

GK+G +K +A A

Sbjct 17099209 GKQGDNNKGDAVEA 17099168

Range 2: 17100513 to 17100740 [GenBank](https://www.ncbi.nlm.nih.gov/nucleotide/CP027624.1?report=genbank&log$=nuclalign&blast_rank=2&RID=BB7A4H83016&from=17100513&to=17100740) [Graphics](https://www.ncbi.nlm.nih.gov/nuccore/CP027624.1?report=graph&rid=BB7A4H83016%5bCP027624.1%5d&tracks=%5bkey:sequence_track,name:Sequence,display_name:Sequence,id:STD1,category:Sequence,annots:Sequence,ShowLabel:true%5d%5bkey:gene_model_track,CDSProductFeats:false%5d%5bkey:alignment_track,name:other%20alignments,annots:NG%20Alignments|Refseq%20Alignments|Gnomon%20Alignments|Unnamed,shown:false%5d&v=17100502:17100751&appname=ncbiblast&link_loc=fromHSP)

| Alignment statistics for match #2 | | | | | | |
| --- | --- | --- | --- | --- | --- | --- |
| **Score** | **Expect** | **Method** | **Identities** | **Positives** | **Gaps** | **Frame** |
| 49.7 bits(117) | 4e-06 | Compositional matrix adjust. | 38/81(47%) | 47/81(58%) | 9/81(11%) | -2 |

Query 14 VATAAGRNNNAFPPSSSRNNKAPADLFITPKTTT--TVKA--AAVSCKRPYPKGAVAAAT 69

VAT AG NNAF PS NK P +LF+ P TT TVKA ++ SCKRPYPKG A+

Sbjct 17100740 VATGAGGRNNAFLPSK---NKTP-NLFLNPNKTTSSTVKAVVSSSSCKRPYPKGD-ASLF 17100576

Query 70 STLSPISGKDGGLRNQEESDG 90

+ + GKD + + DG

Sbjct 17100575 LGIDDVFGKDAVAGHDNDQDG 17100513

Range 3: 17099396 to 17099557 [GenBank](https://www.ncbi.nlm.nih.gov/nucleotide/CP027624.1?report=genbank&log$=nuclalign&blast_rank=2&RID=BB7A4H83016&from=17099396&to=17099557) [Graphics](https://www.ncbi.nlm.nih.gov/nuccore/CP027624.1?report=graph&rid=BB7A4H83016%5bCP027624.1%5d&tracks=%5bkey:sequence_track,name:Sequence,display_name:Sequence,id:STD1,category:Sequence,annots:Sequence,ShowLabel:true%5d%5bkey:gene_model_track,CDSProductFeats:false%5d%5bkey:alignment_track,name:other%20alignments,annots:NG%20Alignments|Refseq%20Alignments|Gnomon%20Alignments|Unnamed,shown:false%5d&v=17099388:17099565&appname=ncbiblast&link_loc=fromHSP)

| Alignment statistics for match #3 | | | | | | |
| --- | --- | --- | --- | --- | --- | --- |
| **Score** | **Expect** | **Method** | **Identities** | **Positives** | **Gaps** | **Frame** |
| 45.4 bits(106) | 1e-04 | Compositional matrix adjust. | 30/70(43%) | 38/70(54%) | 16/70(22%) | -3 |

Query 108 YNGAAALRDQEESDGMLIPPFFVIFGKEGCQDIGHKYNNAAAAGALRDQEESDGILVPPF 167

+N AAA + +D ML+P FF IFGKEG Q ++ EES ++ PF

Sbjct 17099557 HNDAAASGQEMAADDMLMP-FFWIFGKEGQQ---------------QEAEESSDDMLMPF 17099426

Query 168 FLIFGKEGSQ 177

F IFGKEG Q

Sbjct 17099425 FWIFGKEGQQ 17099396

This hit corresponds to a known linusorb precursor protein G14-170N containing linusorbs A1-A3.

The above alignments correspond to matches between the query G11-516P and both exons of G14-170N. The translated ORFs of both exons are as follows:

The first exon (Range 2):

>CP027624.1_145052 [17100782 - 17100429] (REVERSE SENSE) Linum usitatissimum chromosome Lu14

MAAASSLALATASLVATGAGGRNNAFLPSKNKTPNLFLNPNKTTSSTVKAVVSSSSCKRP

YPKGDASLFLGIDDVFGKDAVAGHDNDQDGLLFPLLLFILGMARTRCRKLFRYRLDAY

The second exon (Ranges 1 and 3):

>CP027624.1_145056 [17099527 - 17099156] (REVERSE SENSE) Linum usitatissimum chromosome Lu14

MAADDMLMPFFWIFGKEGQQQEAEESSDDMLMPFFWIFGKEGQQQEAESSDDMLLPFFWI

FGKEGQQQEAESSDDMLMPFFWIFGKQQQQQGESSDDMLMPFFWVFGKQGDNNKGDAVEA

ILKN

# >G11-514P Linusorb C1 precursor protein

MAASSVPLTTSLVATAAAGRNNNSKTPANLFLTPKTSTVKAAVSCKLSGSHHHHHQEEGSGGGDDMLKPFFFWIFG


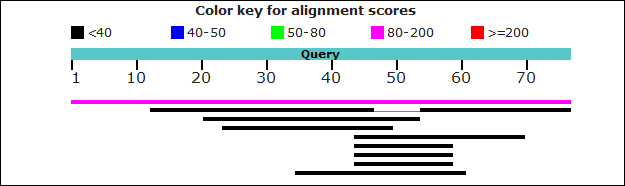


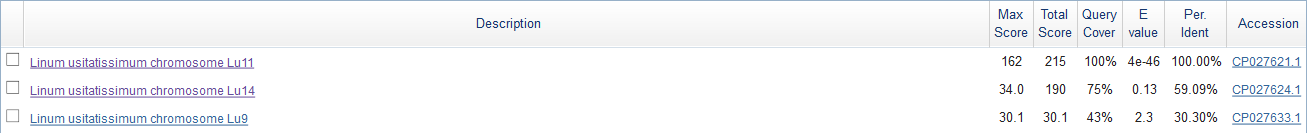


No potential homologue is identified for G11-514P, as the second hit on Chr 14 has an E value of 0.13. Nonetheless, the alignment reveals some interesting findings:

Linum usitatissimum chromosome Lu14

Sequence ID: [CP027624.1](https://www.ncbi.nlm.nih.gov/nucleotide/CP027624.1?report=genbank&log$=nuclalign&blast_rank=2&RID=BXXFNKE5014) Length: 19392306 Number of Matches: 6

Range 1: 17100609 to 17100740 [GenBank](https://www.ncbi.nlm.nih.gov/nucleotide/CP027624.1?report=genbank&log$=nuclalign&blast_rank=2&RID=BXXFNKE5014&from=17100609&to=17100740) [Graphics](https://www.ncbi.nlm.nih.gov/nuccore/CP027624.1?report=graph&rid=BXXFNKE5014%5bCP027624.1%5d&tracks=%5bkey:sequence_track,name:Sequence,display_name:Sequence,id:STD1,category:Sequence,annots:Sequence,ShowLabel:true%5d%5bkey:gene_model_track,CDSProductFeats:false%5d%5bkey:alignment_track,name:other%20alignments,annots:NG%20Alignments|Refseq%20Alignments|Gnomon%20Alignments|Unnamed,shown:false%5d&v=17100603:17100746&appname=ncbiblast&link_loc=fromHSP)

| Alignment statistics for match #2 | | | | | | |
| --- | --- | --- | --- | --- | --- | --- |
| **Score** | **Expect** | **Method** | **Identities** | **Positives** | **Gaps** | **Frame** |
| 32.7 bits(67) | 0.30 | Compositional matrix adjust. | 25/45(56%) | 26/45(57%) | 12/45(26%) | -2 |

Query 13 VATAAAGRNN-----NSKTPANLFLTPK---TSTVKAAV---SCK 46

VAT A GRNN KTP NLFL P +STVKA V SCK

Sbjct 17100740 VATGAGGRNNAFLPSKNKTP-NLFLNPNKTTSSTVKAVVSSSSCK 17100609

The subject turns out to be the linusorb precursor protein G14-170N, and this alignment shows this range is in the signal peptide region.

Range 2: 17099411 to 17099473 [GenBank](https://www.ncbi.nlm.nih.gov/nucleotide/CP027624.1?report=genbank&log$=nuclalign&blast_rank=2&RID=BXXFNKE5014&from=17099411&to=17099473) [Graphics](https://www.ncbi.nlm.nih.gov/nuccore/CP027624.1?report=graph&rid=BXXFNKE5014%5bCP027624.1%5d&tracks=%5bkey:sequence_track,name:Sequence,display_name:Sequence,id:STD1,category:Sequence,annots:Sequence,ShowLabel:true%5d%5bkey:gene_model_track,CDSProductFeats:false%5d%5bkey:alignment_track,name:other%20alignments,annots:NG%20Alignments|Refseq%20Alignments|Gnomon%20Alignments|Unnamed,shown:false%5d&v=17099408:17099476&appname=ncbiblast&link_loc=fromHSP)

| Alignment statistics for match #1 | | | | | | |
| --- | --- | --- | --- | --- | --- | --- |
| **Score** | **Expect** | **Method** | **Identities** | **Positives** | **Gaps** | **Frame** |
| 34.0 bits(70) | 0.13 | Composition-based stats. | 13/22(59%) | 15/22(68%) | 1/22(4%) | -3 |

Query 55 HQEEGSGGGDDMLKPFFFWIFG 76

+Q+E DDML P FFWIFG

Sbjct 17099473 QQQEAEESSDDMLMP-FFWIFG 17099411

Range 3: 17099273 to 17099332 [GenBank](https://www.ncbi.nlm.nih.gov/nucleotide/CP027624.1?report=genbank&log$=nuclalign&blast_rank=2&RID=BXXFNKE5014&from=17099273&to=17099332) [Graphics](https://www.ncbi.nlm.nih.gov/nuccore/CP027624.1?report=graph&rid=BXXFNKE5014%5bCP027624.1%5d&tracks=%5bkey:sequence_track,name:Sequence,display_name:Sequence,id:STD1,category:Sequence,annots:Sequence,ShowLabel:true%5d%5bkey:gene_model_track,CDSProductFeats:false%5d%5bkey:alignment_track,name:other%20alignments,annots:NG%20Alignments|Refseq%20Alignments|Gnomon%20Alignments|Unnamed,shown:false%5d&v=17099271:17099334&appname=ncbiblast&link_loc=fromHSP)

| Alignment statistics for match #3 | | | | | | |
| --- | --- | --- | --- | --- | --- | --- |
| **Score** | **Expect** | **Method** | **Identities** | **Positives** | **Gaps** | **Frame** |
| 31.4 bits(64) | 0.80 | Composition-based stats. | 12/21(57%) | 15/21(71%) | 1/21(4%) | -3 |

Query 56 QEEGSGGGDDMLKPFFFWIFG 76

Q++ + DDML P FFWIFG

Sbjct 17099332 QQQEAESSDDMLMP-FFWIFG 17099273

Range 4: 17099342 to 17099401 [GenBank](https://www.ncbi.nlm.nih.gov/nucleotide/CP027624.1?report=genbank&log$=nuclalign&blast_rank=2&RID=BXXFNKE5014&from=17099342&to=17099401) [Graphics](https://www.ncbi.nlm.nih.gov/nuccore/CP027624.1?report=graph&rid=BXXFNKE5014%5bCP027624.1%5d&tracks=%5bkey:sequence_track,name:Sequence,display_name:Sequence,id:STD1,category:Sequence,annots:Sequence,ShowLabel:true%5d%5bkey:gene_model_track,CDSProductFeats:false%5d%5bkey:alignment_track,name:other%20alignments,annots:NG%20Alignments|Refseq%20Alignments|Gnomon%20Alignments|Unnamed,shown:false%5d&v=17099340:17099403&appname=ncbiblast&link_loc=fromHSP)

| Alignment statistics for match #4 | | | | | | |
| --- | --- | --- | --- | --- | --- | --- |
| **Score** | **Expect** | **Method** | **Identities** | **Positives** | **Gaps** | **Frame** |
| 31.0 bits(63) | 1.2 | Composition-based stats. | 12/21(57%) | 15/21(71%) | 1/21(4%) | -3 |

Query 56 QEEGSGGGDDMLKPFFFWIFG 76

Q++ + DDML P FFWIFG

Sbjct 17099401 QQQEAESSDDMLLP-FFWIFG 17099342

Range 5: 17099207 to 17099269 [GenBank](https://www.ncbi.nlm.nih.gov/nucleotide/CP027624.1?report=genbank&log$=nuclalign&blast_rank=2&RID=BXXFNKE5014&from=17099207&to=17099269) [Graphics](https://www.ncbi.nlm.nih.gov/nuccore/CP027624.1?report=graph&rid=BXXFNKE5014%5bCP027624.1%5d&tracks=%5bkey:sequence_track,name:Sequence,display_name:Sequence,id:STD1,category:Sequence,annots:Sequence,ShowLabel:true%5d%5bkey:gene_model_track,CDSProductFeats:false%5d%5bkey:alignment_track,name:other%20alignments,annots:NG%20Alignments|Refseq%20Alignments|Gnomon%20Alignments|Unnamed,shown:false%5d&v=17099204:17099272&appname=ncbiblast&link_loc=fromHSP)

| Alignment statistics for match #5 | | | | | | |
| --- | --- | --- | --- | --- | --- | --- |
| **Score** | **Expect** | **Method** | **Identities** | **Positives** | **Gaps** | **Frame** |
| 31.0 bits(63) | 1.4 | Composition-based stats. | 12/23(52%) | 17/23(73%) | 2/23(8%) | -3 |

Query 54 HHQEEGSGGGDDMLKPFFFWIFG 76

++Q++G DDML P FFW+FG

Sbjct 17099269 QQQQQGESS-DDMLMP-FFWVFG 17099207

Range 6: 17099483 to 17099557 [GenBank](https://www.ncbi.nlm.nih.gov/nucleotide/CP027624.1?report=genbank&log$=nuclalign&blast_rank=2&RID=BXXFNKE5014&from=17099483&to=17099557) [Graphics](https://www.ncbi.nlm.nih.gov/nuccore/CP027624.1?report=graph&rid=BXXFNKE5014%5bCP027624.1%5d&tracks=%5bkey:sequence_track,name:Sequence,display_name:Sequence,id:STD1,category:Sequence,annots:Sequence,ShowLabel:true%5d%5bkey:gene_model_track,CDSProductFeats:false%5d%5bkey:alignment_track,name:other%20alignments,annots:NG%20Alignments|Refseq%20Alignments|Gnomon%20Alignments|Unnamed,shown:false%5d&v=17099480:17099560&appname=ncbiblast&link_loc=fromHSP)

| Alignment statistics for match #6 | | | | | | |
| --- | --- | --- | --- | --- | --- | --- |
| **Score** | **Expect** | **Method** | **Identities** | **Positives** | **Gaps** | **Frame** |
| 30.6 bits(62) | 1.7 | Composition-based stats. | 14/26(54%) | 14/26(53%) | 4/26(15%) | -3 |

Query 54 HHQEEGSG---GGDDMLKPFFFWIFG 76

H SG DDML P FFWIFG

Sbjct 17099557 HNDAAASGQEMAADDMLMP-FFWIFG 17099483

The above 5 alignments show the linusorb-embedded region of G11-514P matches multiple linusorb-embedded regions of G14-170N.

# >G3-449N_Linusorb D1 precursor protein

MAIASSTFTLALPSLGSSPSPFKGRAHIGLAPVLKARKTSATTLSRETLISHSSKLHHSLLKKSGDAGIGDDGIPPFWLTLFGKQQANVFNSEKGDAGMAPMWVTVFGSERGVFNSEKGDAGMAPVWGTVFGSERGVFNSEKGDAGMAPMWVTVFGSERGVFNLEKGDAGMAPMWVTVFGSERGVFNLEKGDAGMAPVWVTVFGSERGVFNSKKGDASMAPCG


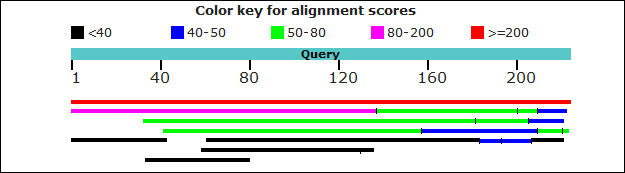


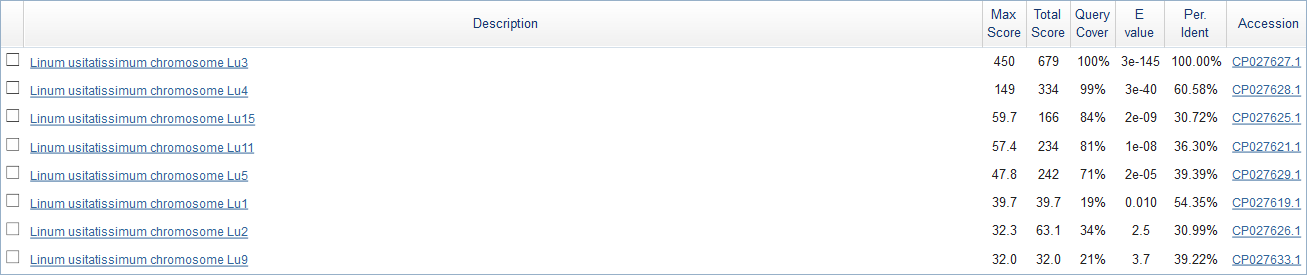


The second hit is taken as the potential homologue. It is the linusorb precursor protein G4-136N.

# Potential homologue:

Linum usitatissimum chromosome Lu4

Sequence ID: [CP027628.1](https://www.ncbi.nlm.nih.gov/nucleotide/CP027628.1?report=genbank&log$=nuclalign&blast_rank=2&RID=B0VP89UW016) Length: 19927942 Number of Matches: 4

Range 1: 13632862 to 13633224 [GenBank](https://www.ncbi.nlm.nih.gov/nucleotide/CP027628.1?report=genbank&log$=nuclalign&blast_rank=2&RID=B0VP89UW016&from=13632862&to=13633224)

| Alignment statistics for match #1 | | | | | | |
| --- | --- | --- | --- | --- | --- | --- |
| **Score** | **Expect** | **Method** | **Identities** | **Positives** | **Gaps** | **Frame** |
| 149 bits(376) | 3e-40 | Compositional matrix adjust. | 83/137(61%) | 94/137(68%) | 17/137(12%) | -2 |

Query 1 MAIASSTFTLALPSLGSSPSPFKGRAHIGLAPVLKARKTSATTLSRETLISHSSKLHHSL 60

+A+ASS FTLALPSLGSSPSPF GRAH+GL PVLKARKT I SSKLH +L

Sbjct 13633224 IAMASSAFTLALPSLGSSPSPFNGRAHVGLPPVLKARKTP---------IVSSSKLHSTL 13633072

Query 61 LKKSG-DAGIGDDGIPPFWLTLFGKQQANVFNSEKGDAGMAPMWVTVFGSERGVFNSEKG 119

K D+ GD GIPPFWLTL GKQ+ +VFNS+ GDAG+ PMWV VFGSER G

Sbjct 13633071 KKHEVVDSERGDAGIPPFWLTLVGKQRTDVFNSKLGDAGLPPMWVEVFGSER-------G 13632913

Query 120 DAGMAPVWGTVFGSERG 136

DAG+ P W T+ G G

Sbjct 13632912 DAGIPPFWLTLIGKHAG 13632862

Range 2: 13632862 to 13633059 [GenBank](https://www.ncbi.nlm.nih.gov/nucleotide/CP027628.1?report=genbank&log$=nuclalign&blast_rank=2&RID=B0VP89UW016&from=13632862&to=13633059)

| Alignment statistics for match #2 | | | | | | |
| --- | --- | --- | --- | --- | --- | --- |
| **Score** | **Expect** | **Method** | **Identities** | **Positives** | **Gaps** | **Frame** |
| 78.2 bits(191) | 1e-15 | Compositional matrix adjust. | 38/73(52%) | 49/73(67%) | 8/73(10%) | -2 |

Query 137 VFNSEKGDAGMAPMWVTVFGSER-GVFNLEKGDAGMAPMWVTVFGSERGVFNLEKGDAGM 195

V +SE+GDAG+ P W+T+ G +R VFN + GDAG+ PMWV VFGSERG DAG+

Sbjct 13633059 VVDSERGDAGIPPFWLTLVGKQRTDVFNSKLGDAGLPPMWVEVFGSERG-------DAGI 13632901

Query 196 APVWVTVFGSERG 208

P W+T+ G G

Sbjct 13632900 PPFWLTLIGKHAG 13632862

Range 3: 4995303 to 4995695 [GenBank](https://www.ncbi.nlm.nih.gov/nucleotide/CP027628.1?report=genbank&log$=nuclalign&blast_rank=2&RID=B0VP89UW016&from=4995303&to=4995695)

| Alignment statistics for match #3 | | | | | | |
| --- | --- | --- | --- | --- | --- | --- |
| **Score** | **Expect** | **Method** | **Identities** | **Positives** | **Gaps** | **Frame** |
| 63.5 bits(153) | 1e-10 | Compositional matrix adjust. | 45/131(34%) | 64/131(48%) | 16/131(12%) | +3 |

Query 85 QQANVFNSEKG----DAGMAPMWVTVFGSERGVFNSEKG----DAGMAPVWGTVFGSERG 136

Q+ VF+ E G + G+ M VF E GVF+ E G + G+ + VF E G

Sbjct 4995303 QEHGVFHMEHGVFHMEHGVFHMEHGVFHMEHGVFHMEHGVFHMEHGVFHMEHGVFHMEHG 4995482

Query 137 VFNSEKG----DAGMAPMWVTVFGSERGVFNLEKG----DAGMAPMWVTVFGSERGVFNL 188

VF+ E G + G+ M VF E GVF++E G + G+ M VF E GVF++

Sbjct 4995483 VFHMEHGVFHMEHGVFHMEHGVFHMEHGVFHMEHGVFHMEHGVFHMEHGVFHMEHGVFHM 4995662

Query 189 EKGDAGMAPVW 199

E G P++

Sbjct 4995663 EHGVFHPRPIF 4995695

Range 4: 13632946 to 13633059 [GenBank](https://www.ncbi.nlm.nih.gov/nucleotide/CP027628.1?report=genbank&log$=nuclalign&blast_rank=2&RID=B0VP89UW016&from=13632946&to=13633059)

| Alignment statistics for match #4 | | | | | | |
| --- | --- | --- | --- | --- | --- | --- |
| **Score** | **Expect** | **Method** | **Identities** | **Positives** | **Gaps** | **Frame** |
| 43.5 bits(101) | 7e-04 | Compositional matrix adjust. | 20/38(53%) | 27/38(71%) | 1/38(2%) | -2 |

Query 185 VFNLEKGDAGMAPVWVTVFGSER-GVFNSKKGDASMAP 221

V + E+GDAG+ P W+T+ G +R VFNSK GDA + P

Sbjct 13633059 VVDSERGDAGIPPFWLTLVGKQRTDVFNSKLGDAGLPP 13632946

Ranges 1, 2 and 4 are local alignments of the leader peptides between both precursor proteins, and Range 3 is the alignment of linusorb-embedded regions. Although this evidence is robust enough to determine their potential homology, the C-terminal end of the linusorb-embedded region contains a long gap caused by different numbers and sequence variations of repeats between two proteins.

G3-449N MAIASSTFTLALPSLGSSPSPFKGRAHIGLAPVLKARKTSATTLSRETLISHSSKLHHSL

G4-136N --MASSAFTLALPSLGSSPSPFNGRAHVGLPPVLKARKTP---------IVSSSKLHSTL

:***:***************:****:**.********. * ***** :*

G3-449N LKKS-GDAGIGDDGIPPFWLTLFGKQQANVFNSEKGDAGMAPMWVTVFGSERGVFNSEKG

G4-136N KKHEVVDSERGDAGIPPFWLTLVGKQRTDVFNSKLGDAGLPPMWVEVFGSER--------

*:. *: ** *********.***.::****: ****:.**** ******

G3-449N DAGMAPVWGTVFGSERGVFNSEKGDAGMAPMWVTVFGSERGVFNLEKGDAGMAPMWVTVF

G4-136N -----------------------------------------------GDAGIPPFWLTLI

****:.*:*:*::

G3-449N GSERGVFNLEKGDAGMAPVWVTVFGSERGVFNSKKGDASMAPCG

G4-136N GKHAG-----------------------QIVDSTSVNT------

*. * :.:*.. ::

# >G4-136N_Linusorb D1 precursor protein

MASSAFTLALPSLGSSPSPFNGRAHVGLPPVLKARKTPIVSSSKLHSTLKKHEVVDSERGDAGIPPFWLTLVGKQRTDVFNSKLGDAGLPPMWVEVFGSERGDAGIPPFWLTLIGKHAGQIVDSTSVNT


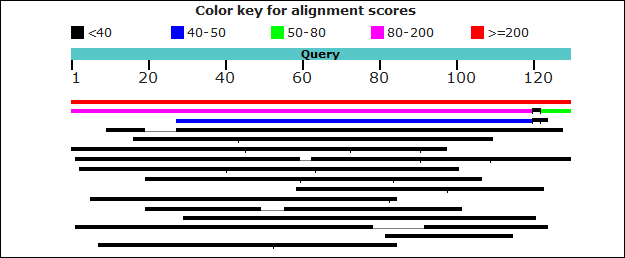


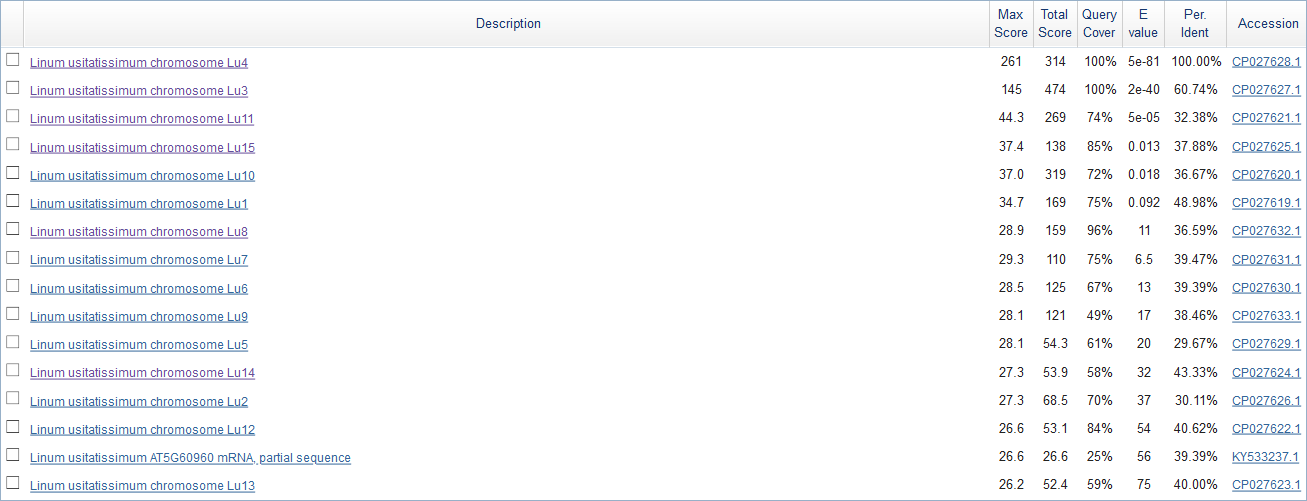


The second top hit is taken as potential homologue. It is the linusorb precursor protein G3-449N.

# Potential homologue:

Linum usitatissimum chromosome Lu3

Sequence ID: [CP027627.1](https://www.ncbi.nlm.nih.gov/nucleotide/CP027627.1?report=genbank&log$=nuclalign&blast_rank=2&RID=BR89SDBT016) Length: 26636119 Number of Matches: 8

Range 1: 4494631 to 4495032 [GenBank](https://www.ncbi.nlm.nih.gov/nucleotide/CP027627.1?report=genbank&log$=nuclalign&blast_rank=2&RID=BR89SDBT016&from=4494631&to=4495032) [Graphics](https://www.ncbi.nlm.nih.gov/nuccore/CP027627.1?report=graph&rid=BR89SDBT016%5bCP027627.1%5d&tracks=%5bkey:sequence_track,name:Sequence,display_name:Sequence,id:STD1,category:Sequence,annots:Sequence,ShowLabel:true%5d%5bkey:gene_model_track,CDSProductFeats:false%5d%5bkey:alignment_track,name:other%20alignments,annots:NG%20Alignments|Refseq%20Alignments|Gnomon%20Alignments|Unnamed,shown:false%5d&v=4494611:4495052&appname=ncbiblast&link_loc=fromHSP)

| Alignment statistics for match #1 | | | | | | |
| --- | --- | --- | --- | --- | --- | --- |
| **Score** | **Expect** | **Method** | **Identities** | **Positives** | **Gaps** | **Frame** |
| 145 bits(366) | 2e-40 | Compositional matrix adjust. | 82/135(61%) | 93/135(68%) | 17/135(12%) | -2 |

Query 1 MASSAFTLALPSLGSSPSPFNGRAHVGLPPVLKARKTP---------IVSSSKLHSTLKK 51

+ASS FTLALPSLGSSPSPF GRAH+GL PVLKARKT I SSKLH +L K

Sbjct 4495032 IASSTFTLALPSLGSSPSPFKGRAHIGLAPVLKARKTSATTLSRETLISHSSKLHHSLLK 4494853

Query 52 HEVVDSERGDAGIPPFWLTLVGKQRTDVFNSKLGDAGLPPMWVEVFGSER-------GDA 104

+ D+ GD GIPPFWLTL GKQ+ +VFNS+ GDAG+ PMWV VFGSER GDA

Sbjct 4494852 -KSGDAGIGDDGIPPFWLTLFGKQQANVFNSEKGDAGMAPMWVTVFGSERGVFNSEKGDA 4494676

Query 105 GIPPFWLTLIGKHAG 119

G+ P W T+ G G

Sbjct 4494675 GMAPVWGTVFGSERG 4494631

Range 2: 4494263 to 4494376 [GenBank](https://www.ncbi.nlm.nih.gov/nucleotide/CP027627.1?report=genbank&log$=nuclalign&blast_rank=2&RID=BR89SDBT016&from=4494263&to=4494376) [Graphics](https://www.ncbi.nlm.nih.gov/nuccore/CP027627.1?report=graph&rid=BR89SDBT016%5bCP027627.1%5d&tracks=%5bkey:sequence_track,name:Sequence,display_name:Sequence,id:STD1,category:Sequence,annots:Sequence,ShowLabel:true%5d%5bkey:gene_model_track,CDSProductFeats:false%5d%5bkey:alignment_track,name:other%20alignments,annots:NG%20Alignments|Refseq%20Alignments|Gnomon%20Alignments|Unnamed,shown:false%5d&v=4494258:4494381&appname=ncbiblast&link_loc=fromHSP)

| Alignment statistics for match #2 | | | | | | |
| --- | --- | --- | --- | --- | --- | --- |
| **Score** | **Expect** | **Method** | **Identities** | **Positives** | **Gaps** | **Frame** |
| 71.2 bits(173) | 1e-19 | Compositional matrix adjust. | 31/38(82%) | 35/38(92%) | 0/38(0%) | -1 |

Query 92 MWVEVFGSERGDAGIPPFWLTLIGKHAGQIVDSTSVNT 129

+WV VFGSE+GDAGIPPFWLTL+GK AGQ+VDS SVNT

Sbjct 4494376 VWVTVFGSEKGDAGIPPFWLTLVGKQAGQVVDSASVNT 4494263

Range 3: 4494376 to 4494486 [GenBank](https://www.ncbi.nlm.nih.gov/nucleotide/CP027627.1?report=genbank&log$=nuclalign&blast_rank=2&RID=BR89SDBT016&from=4494376&to=4494486) [Graphics](https://www.ncbi.nlm.nih.gov/nuccore/CP027627.1?report=graph&rid=BR89SDBT016%5bCP027627.1%5d&tracks=%5bkey:sequence_track,name:Sequence,display_name:Sequence,id:STD1,category:Sequence,annots:Sequence,ShowLabel:true%5d%5bkey:gene_model_track,CDSProductFeats:false%5d%5bkey:alignment_track,name:other%20alignments,annots:NG%20Alignments|Refseq%20Alignments|Gnomon%20Alignments|Unnamed,shown:false%5d&v=4494371:4494491&appname=ncbiblast&link_loc=fromHSP)

| Alignment statistics for match #3 | | | | | | |
| --- | --- | --- | --- | --- | --- | --- |
| **Score** | **Expect** | **Method** | **Identities** | **Positives** | **Gaps** | **Frame** |
| 43.1 bits(100) | 1e-19 | Compositional matrix adjust. | 20/38(53%) | 27/38(71%) | 1/38(2%) | -2 |

Query 54 VVDSERGDAGIPPFWLTLVGKQRTDVFNSKLGDAGLPP 91

V + E+GDAG+ P W+T+ G +R VFNSK GDA + P

Sbjct 4494486 VFNLEKGDAGMAPVWVTVFGSER-GVFNSKKGDASMAP 4494376

Range 4: 4494415 to 4494630 [GenBank](https://www.ncbi.nlm.nih.gov/nucleotide/CP027627.1?report=genbank&log$=nuclalign&blast_rank=2&RID=BR89SDBT016&from=4494415&to=4494630) [Graphics](https://www.ncbi.nlm.nih.gov/nuccore/CP027627.1?report=graph&rid=BR89SDBT016%5bCP027627.1%5d&tracks=%5bkey:sequence_track,name:Sequence,display_name:Sequence,id:STD1,category:Sequence,annots:Sequence,ShowLabel:true%5d%5bkey:gene_model_track,CDSProductFeats:false%5d%5bkey:alignment_track,name:other%20alignments,annots:NG%20Alignments|Refseq%20Alignments|Gnomon%20Alignments|Unnamed,shown:false%5d&v=4494405:4494640&appname=ncbiblast&link_loc=fromHSP)

| Alignment statistics for match #4 | | | | | | |
| --- | --- | --- | --- | --- | --- | --- |
| **Score** | **Expect** | **Method** | **Identities** | **Positives** | **Gaps** | **Frame** |
| 77.4 bits(189) | 1e-16 | Compositional matrix adjust. | 38/73(52%) | 49/73(67%) | 8/73(10%) | -2 |

Query 54 VVDSERGDAGIPPFWLTLVGKQRTDVFNSKLGDAGLPPMWVEVFGSERG-------DAGI 106

V +SE+GDAG+ P W+T+ G +R VFN + GDAG+ PMWV VFGSERG DAG+

Sbjct 4494630 VFNSEKGDAGMAPMWVTVFGSER-GVFNLEKGDAGMAPMWVTVFGSERGVFNLEKGDAGM 4494454

Query 107 PPFWLTLIGKHAG 119

P W+T+ G G

Sbjct 4494453 APVWVTVFGSERG 4494415

Range 5: 4494278 to 4494364 [GenBank](https://www.ncbi.nlm.nih.gov/nucleotide/CP027627.1?report=genbank&log$=nuclalign&blast_rank=2&RID=BR89SDBT016&from=4494278&to=4494364) [Graphics](https://www.ncbi.nlm.nih.gov/nuccore/CP027627.1?report=graph&rid=BR89SDBT016%5bCP027627.1%5d&tracks=%5bkey:sequence_track,name:Sequence,display_name:Sequence,id:STD1,category:Sequence,annots:Sequence,ShowLabel:true%5d%5bkey:gene_model_track,CDSProductFeats:false%5d%5bkey:alignment_track,name:other%20alignments,annots:NG%20Alignments|Refseq%20Alignments|Gnomon%20Alignments|Unnamed,shown:false%5d&v=4494274:4494368&appname=ncbiblast&link_loc=fromHSP)

| Alignment statistics for match #5 | | | | | | |
| --- | --- | --- | --- | --- | --- | --- |
| **Score** | **Expect** | **Method** | **Identities** | **Positives** | **Gaps** | **Frame** |
| 45.8 bits(107) | 2e-05 | Compositional matrix adjust. | 21/29(72%) | 23/29(79%) | 0/29(0%) | -1 |

Query 54 VVDSERGDAGIPPFWLTLVGKQRTDVFNS 82

V SE+GDAGIPPFWLTLVGKQ V +S

Sbjct 4494364 VFGSEKGDAGIPPFWLTLVGKQAGQVVDS 4494278

The 5 alignments are equivalent to those in the G3-449N section discussed above.
