# Supplementary material for: The flax genome reveals orbitide diversity
Source: BMC Genomics. 2022 Jul 23;23:534. doi: 10.1186/s12864-022-08735-x (PMC9308333; doi:10.1186/s12864-022-08735-x)
Supplement: Supplementary file 3 — Additional file 3: Data S2. RADAR-detected repetitive motifs in linusorb precursor proteins. [file 12864_2022_8735_MOESM3_ESM.docx]

Supplementary File S3. Alignment of repeats in the linusorb precursor proteins generated by RADAR.

Notations: Length, length of alignment; Diagonal, length of the selected repeat diagonal; BW, best window; BW-From, starting position of the best window; BW-To, ending position of the best window; Level, depth of recursion; the alignment row reports the range of repeats from the first to the last residue; the parenthesis encloses the alignment score and Z-score of the repeat (Heger and Holm, 2000). The coloring scheme for residues is default setting by RADAR.

>G14-170N_Linusorb A1-A3 precursor protein

MAAASSLALATASLVATGAGGRNNAFLPSKNKTPNLFLNPNKTTSSTVKAVVSSSSCKRP

YPKGDASLFLGIDDVFGKDAVAGHDNDQDAASGQEMAADDMLMPFFWIFGKEGQQQEAEE

SSDDMLMPFFWIFGKEGQQQEAESSDDMLLPFFWIFGKEGQQQEAESSDDMLMPFFWIFG

KQQQQQGESSDDMLMPFFWVFGKQGDNNKGDAVEAILKN

---------------------------------------------------------------------------

No. of Repeats|Total Score|Length |Diagonal| BW-From| BW-To| Level

5| 244.83| 21| 21| 99| 119| 1

---------------------------------------------------------------------------

99- 119 (52.73/27.86) DDMLMPFFWIFGKEGQQQEAE

123- 143 (52.73/27.86) DDMLMPFFWIFGKEGQQQEAE

146- 165 (44.73/22.74) DDMLLPFFWIFGKEGQQQEA.

169- 187 (47.39/24.44) DDMLMPFFWIFGK..QQQQQG

191- 205 (47.25/24.35) DDMLMPFFWVFGK......QG

---------------------------------------------------------------------------

>G11-516P_Linusorb B1-B3 precursor protein

MAVVSSLALTTSLVATAAGRNNNAFPPSSSRNNKAPADLFITPKTTTTVKAAAVSCKRPY

PKGAVAAATSTLSPISGKDGGLRNQEESDGMLVFPLFIFGKEGSQDKYNGAAALRDQEES

DGMLIPPFFVIFGKEGCQDIGHKYNNAAAAGALRDQEESDGILVPPFFLIFGKEGSQDKY

NAAAAGGLRGKEQQGDKMAAGAEN

---------------------------------------------------------------------------

No. of Repeats|Total Score|Length |Diagonal| BW-From| BW-To| Level

4| 219.53| 33| 69| 80| 112| 1

---------------------------------------------------------------------------

80- 112 (71.89/43.80) GGLRNQEESDGMLVFPLF.IFGKEGSQD...KYNGAA

117- 148 (48.27/29.16) .....QEESDGMLIPPFFvIFGKEGCQDighKYNNAA

151- 184 (67.30/40.38) GALRDQEESDGILVPPFFlIFGKEGSQD...KYNAAA

186- 202 (32.06/18.69) GGLR.................GKEQQGD...KMAAGA

---------------------------------------------------------------------------

>G4-136N_Linusorb D1 precursor protein

MASSAFTLALPSLGSSPSPFNGRAHVGLPPVLKARKTPIVSSSKLHSTLKKHEVVDSERG

DAGIPPFWLTLVGKQRTDVFNSKLGDAGLPPMWVEVFGSERGDAGIPPFWLTLIGKHAGQ

IVDSTSVNT

---------------------------------------------------------------------------

No. of Repeats|Total Score|Length |Diagonal| BW-From| BW-To| Level

3| 138.36| 32| 39| 60| 74| 1

---------------------------------------------------------------------------

60- 74 (32.20/15.22) GDAGIPPFWLTLVGK

85- 101 (63.87/49.81) GDAGLPPMWVEVFGSER

102- 116 (42.28/19.65) GDAGIPPFWLTLIGK

---------------------------------------------------------------------------

>G3-449N_Linusorb D1 precursor protein

MAIASSTFTLALPSLGSSPSPFKGRAHIGLAPVLKARKTSATTLSRETLISHSSKLHHSL

LKKSGDAGIGDDGIPPFWLTLFGKQQANVFNSEKGDAGMAPMWVTVFGSERGVFNSEKGD

AGMAPVWGTVFGSERGVFNSEKGDAGMAPMWVTVFGSERGVFNLEKGDAGMAPMWVTVFG

SERGVFNLEKGDAGMAPVWVTVFGSERGVFNSKKGDASMAPCG

---------------------------------------------------------------------------

No. of Repeats|Total Score|Length |Diagonal| BW-From| BW-To| Level

6| 313.84| 22| 22| 142| 163| 1

---------------------------------------------------------------------------

70- 91 (32.11/14.10) .GDDGIPPFWLTLFGKQQAnVFN

94- 115 (57.00/29.59) KGDAGMAPMWVTVFGSERG.VFN

118- 139 (55.07/28.39) KGDAGMAPVWGTVFGSERG.VFN

142- 163 (57.00/29.59) KGDAGMAPMWVTVFGSERG.VFN

166- 187 (57.00/29.59) KGDAGMAPMWVTVFGSERG.VFN

190- 211 (55.67/28.76) KGDAGMAPVWVTVFGSERG.VFN

---------------------------------------------------------------------------

>G11-514N_Linusorb C1 precursor protein

MAASSVPLTTSLVATAAAGRNNNSKTPANLFLTPKTSTVKAAVSCKLSGSHHHHHQEEGS

GGGDDMLKPFFFWIFG

Only one linusorb domain MLKPFFFWI is present, so no repeat is detected.
