# Supplementary material for: The flax genome reveals orbitide diversity
Source: BMC Genomics. 2022 Jul 23;23:534. doi: 10.1186/s12864-022-08735-x (PMC9308333; doi:10.1186/s12864-022-08735-x)
Supplement: Supplementary file 4 — Additional file 4: Data S3. Repeat PSI-BLAST output. [file 12864_2022_8735_MOESM4_ESM.docx]

PSIBLAST 2.10.1+

Reference: Stephen F. Altschul, Thomas L. Madden, Alejandro A.

Schaffer, Jinghui Zhang, Zheng Zhang, Webb Miller, and David J.

Lipman (1997), "Gapped BLAST and PSI-BLAST: a new generation of

protein database search programs", Nucleic Acids Res. 25:3389-3402.

Reference for compositional score matrix adjustment: Stephen F.

Altschul, John C. Wootton, E. Michael Gertz, Richa Agarwala,

Aleksandr Morgulis, Alejandro A. Schaffer, and Yi-Kuo Yu (2005)

"Protein database searches using compositionally adjusted

substitution matrices", FEBS J. 272:5101-5109.

Reference for composition-based statistics starting in round 2:

Alejandro A. Schaffer, L. Aravind, Thomas L. Madden, Sergei

Shavirin, John L. Spouge, Yuri I. Wolf, Eugene V. Koonin, and

Stephen F. Altschul (2001), "Improving the accuracy of PSI-BLAST

protein database searches with composition-based statistics and

other refinements", Nucleic Acids Res. 29:2994-3005.

Database: LuBethune_augustus-hints.aa

60,181 sequences; 21,786,600 total letters

Results from round 1

Query=

Length=33

Score

Sequences producing significant alignments: (Bits) E Value

g24919.t1 51.7 3e-10

g7437.t1 36.7 1e-04

g53356.t1 31.7 0.006

g33422.t1 31.3 0.007

g28070.t1 26.7 0.48

g23910.t1 25.5 1.2

g51918.t1 25.5 1.2

g34966.t1 24.0 3.9

>g24919.t1

Length=204

Score = 51.7 bits (122), Expect = 3e-10, Method: Composition-based stats.

Identities = 33/33 (100%), Positives = 33/33 (100%), Gaps = 0/33 (0%)

Query 1 GGLRNQEESDGMLVFPLFIFGKEGSQDKYNGAA 33

GGLRNQEESDGMLVFPLFIFGKEGSQDKYNGAA

Sbjct 80 GGLRNQEESDGMLVFPLFIFGKEGSQDKYNGAA 112

Score = 48.2 bits (113), Expect = 6e-09, Method: Composition-based stats.

Identities = 27/34 (79%), Positives = 29/34 (85%), Gaps = 1/34 (3%)

Query 1 GGLRNQEESDGMLVFPLF-IFGKEGSQDKYNGAA 33

G LR+QEESDG+LV P F IFGKEGSQDKYN AA

Sbjct 151 GALRDQEESDGILVPPFFLIFGKEGSQDKYNAAA 184

Score = 42.8 bits (99), Expect = 6e-07, Method: Composition-based stats.

Identities = 25/37 (68%), Positives = 27/37 (73%), Gaps = 4/37 (11%)

Query 1 GGLRNQEESDGMLVFPLF-IFGKEGSQD---KYNGAA 33

LR+QEESDGML+ P F IFGKEG QD KYN AA

Sbjct 112 AALRDQEESDGMLIPPFFVIFGKEGCQDIGHKYNNAA 148

>g7437.t1

Length=219

Score = 36.7 bits (83), Expect = 1e-04, Method: Composition-based stats.

Identities = 13/33 (39%), Positives = 18/33 (55%), Gaps = 0/33 (0%)

Query 1 GGLRNQEESDGMLVFPLFIFGKEGSQDKYNGAA 33

G + E SD ML+ +IFGKEG Q + +

Sbjct 137 GQQQEAESSDDMLLPFFWIFGKEGQQQEAESSD 169

Score = 36.3 bits (82), Expect = 2e-04, Method: Composition-based stats.

Identities = 12/32 (38%), Positives = 17/32 (53%), Gaps = 0/32 (0%)

Query 2 GLRNQEESDGMLVFPLFIFGKEGSQDKYNGAA 33

+E SD ML+ +IFGKEG Q + +

Sbjct 115 QQEAEESSDDMLMPFFWIFGKEGQQQEAESSD 146

Score = 35.5 bits (80), Expect = 3e-04, Method: Composition-based stats.

Identities = 11/33 (33%), Positives = 16/33 (48%), Gaps = 0/33 (0%)

Query 1 GGLRNQEESDGMLVFPLFIFGKEGSQDKYNGAA 33

G + E SD ML+ +IFGK+ Q +

Sbjct 160 GQQQEAESSDDMLMPFFWIFGKQQQQQGESSDD 192

Score = 33.6 bits (75), Expect = 0.001, Method: Composition-based stats.

Identities = 10/32 (31%), Positives = 17/32 (53%), Gaps = 0/32 (0%)

Query 2 GLRNQEESDGMLVFPLFIFGKEGSQDKYNGAA 33

+ E SD ML+ ++FGK+G +K +

Sbjct 183 QQQQGESSDDMLMPFFWVFGKQGDNNKGDAVE 214

Score = 31.7 bits (70), Expect = 0.007, Method: Composition-based stats.

Identities = 10/25 (40%), Positives = 16/25 (64%), Gaps = 0/25 (0%)

Query 9 SDGMLVFPLFIFGKEGSQDKYNGAA 33

+D ML+ +IFGKEG Q + ++

Sbjct 98 ADDMLMPFFWIFGKEGQQQEAEESS 122

>g53356.t1

Length=223

Score = 31.7 bits (70), Expect = 0.006, Method: Composition-based stats.

Identities = 4/28 (14%), Positives = 10/28 (36%), Gaps = 1/28 (4%)

Query 5 NQEESDGMLVFPLF-IFGKEGSQDKYNG 31

+ D + +FGK+ + +

Sbjct 66 DAGIGDDGIPPFWLTLFGKQQANVFNSE 93

>g33422.t1

Length=129

Score = 31.3 bits (69), Expect = 0.007, Method: Composition-based stats.

Identities = 4/27 (15%), Positives = 8/27 (30%), Gaps = 0/27 (0%)

Query 5 NQEESDGMLVFPLFIFGKEGSQDKYNG 31

+ E D + + D +N

Sbjct 56 DSERGDAGIPPFWLTLVGKQRTDVFNS 82

Score = 31.3 bits (69), Expect = 0.008, Method: Composition-based stats.

Identities = 5/23 (22%), Positives = 7/23 (30%), Gaps = 1/23 (4%)

Query 5 NQEESDGMLVFPLF-IFGKEGSQ 26

E D + + GK Q

Sbjct 98 GSERGDAGIPPFWLTLIGKHAGQ 120

>g28070.t1

Length=196

Score = 26.7 bits (57), Expect = 0.48, Method: Composition-based stats.

Identities = 10/27 (37%), Positives = 12/27 (44%), Gaps = 0/27 (0%)

Query 5 NQEESDGMLVFPLFIFGKEGSQDKYNG 31

QE+ D +FGKE YNG

Sbjct 48 GQEKGDAGYPPLSPLFGKEKGDAGYNG 74

>g23910.t1

Length=1152

Score = 25.5 bits (54), Expect = 1.2, Method: Composition-based stats.

Identities = 6/16 (38%), Positives = 7/16 (44%), Gaps = 0/16 (0%)

Query 11 GMLVFPLFIFGKEGSQ 26

ML FIF + G

Sbjct 430 DMLPAIWFIFSRRGCD 445

>g51918.t1

Length=1125

Score = 25.5 bits (54), Expect = 1.2, Method: Composition-based stats.

Identities = 6/16 (38%), Positives = 7/16 (44%), Gaps = 0/16 (0%)

Query 11 GMLVFPLFIFGKEGSQ 26

ML FIF + G

Sbjct 447 DMLPAIWFIFSRRGCD 462

>g34966.t1

Length=801

Score = 24.0 bits (50), Expect = 3.9, Method: Composition-based stats.

Identities = 6/25 (24%), Positives = 10/25 (40%), Gaps = 0/25 (0%)

Query 5 NQEESDGMLVFPLFIFGKEGSQDKY 29

Q++ D +FG+E Y

Sbjct 112 GQKKGDTGYPPLSPLFGQEKDNTGY 136

Score = 23.6 bits (49), Expect = 5.1, Method: Composition-based stats.

Identities = 7/25 (28%), Positives = 10/25 (40%), Gaps = 0/25 (0%)

Query 5 NQEESDGMLVFPLFIFGKEGSQDKY 29

Q+ D +FG+E S Y

Sbjct 160 GQDNGDAGYPPLSPLFGQEKSDAGY 184

Score = 23.6 bits (49), Expect = 5.7, Method: Composition-based stats.

Identities = 8/25 (32%), Positives = 11/25 (44%), Gaps = 0/25 (0%)

Query 5 NQEESDGMLVFPLFIFGKEGSQDKY 29

QE+SD +FG+E Y

Sbjct 176 GQEKSDAGYPPLSPLFGQEERDAGY 200

Score = 23.6 bits (49), Expect = 5.9, Method: Composition-based stats.

Identities = 8/25 (32%), Positives = 11/25 (44%), Gaps = 0/25 (0%)

Query 5 NQEESDGMLVFPLFIFGKEGSQDKY 29

QE+SD +FG+E Y

Sbjct 224 GQEKSDAGYPPLSPLFGQEKRDAGY 248

Score = 23.2 bits (48), Expect = 8.3, Method: Composition-based stats.

Identities = 8/25 (32%), Positives = 11/25 (44%), Gaps = 0/25 (0%)

Query 5 NQEESDGMLVFPLFIFGKEGSQDKY 29

Q+E D +FG+E S Y

Sbjct 208 GQDERDAGYPPLSPLFGQEKSDAGY 232

Lambda K H a alpha

0.319 0.124 0.304 0.792 4.96

Gapped

Lambda K H a alpha sigma

0.267 0.0380 0.140 1.90 42.6 43.6

Effective search space used: 532628800

Results from round 2

Query=

Length=33

Score

Sequences producing significant alignments: (Bits) E Value

Sequences used in model and found again:

g24919.t1 60.1 2e-13

g7437.t1 53.1 1e-10

Sequences not found previously or not previously below threshold:

g26217.t1 26.2 0.58

g3343.t1 23.5 5.4

>g24919.t1

Length=204

Score = 60.1 bits (144), Expect = 2e-13, Method: Composition-based stats.

Identities = 33/33 (100%), Positives = 33/33 (100%), Gaps = 0/33 (0%)

Query 1 GGLRNQEESDGMLVFPLFIFGKEGSQDKYNGAA 33

GGLRNQEESDGMLVFPLFIFGKEGSQDKYNGAA

Sbjct 80 GGLRNQEESDGMLVFPLFIFGKEGSQDKYNGAA 112

Score = 50.8 bits (120), Expect = 7e-10, Method: Composition-based stats.

Identities = 27/34 (79%), Positives = 29/34 (85%), Gaps = 1/34 (3%)

Query 1 GGLRNQEESDGMLVFPLF-IFGKEGSQDKYNGAA 33

G LR+QEESDG+LV P F IFGKEGSQDKYN AA

Sbjct 151 GALRDQEESDGILVPPFFLIFGKEGSQDKYNAAA 184

Score = 43.1 bits (100), Expect = 6e-07, Method: Composition-based stats.

Identities = 25/35 (71%), Positives = 27/35 (77%), Gaps = 4/35 (11%)

Query 3 LRNQEESDGMLVFPLF-IFGKEGSQD---KYNGAA 33

LR+QEESDGML+ P F IFGKEG QD KYN AA

Sbjct 114 LRDQEESDGMLIPPFFVIFGKEGCQDIGHKYNNAA 148

>g7437.t1

Length=219

Score = 53.1 bits (126), Expect = 1e-10, Method: Composition-based stats.

Identities = 13/33 (39%), Positives = 18/33 (55%), Gaps = 0/33 (0%)

Query 1 GGLRNQEESDGMLVFPLFIFGKEGSQDKYNGAA 33

G + E SD ML+ +IFGKEG Q + +

Sbjct 137 GQQQEAESSDDMLLPFFWIFGKEGQQQEAESSD 169

Score = 48.5 bits (114), Expect = 7e-09, Method: Composition-based stats.

Identities = 14/34 (41%), Positives = 19/34 (56%), Gaps = 1/34 (3%)

Query 1 GGLRNQEES-DGMLVFPLFIFGKEGSQDKYNGAA 33

G + EES D ML+ +IFGKEG Q + +

Sbjct 113 GQQQEAEESSDDMLMPFFWIFGKEGQQQEAESSD 146

Score = 43.5 bits (101), Expect = 4e-07, Method: Composition-based stats.

Identities = 11/33 (33%), Positives = 16/33 (48%), Gaps = 0/33 (0%)

Query 1 GGLRNQEESDGMLVFPLFIFGKEGSQDKYNGAA 33

G + E SD ML+ +IFGK+ Q +

Sbjct 160 GQQQEAESSDDMLMPFFWIFGKQQQQQGESSDD 192

Score = 38.9 bits (89), Expect = 2e-05, Method: Composition-based stats.

Identities = 10/26 (38%), Positives = 16/26 (62%), Gaps = 0/26 (0%)

Query 8 ESDGMLVFPLFIFGKEGSQDKYNGAA 33

+D ML+ +IFGKEG Q + ++

Sbjct 97 AADDMLMPFFWIFGKEGQQQEAEESS 122

Score = 38.1 bits (87), Expect = 4e-05, Method: Composition-based stats.

Identities = 10/30 (33%), Positives = 17/30 (57%), Gaps = 0/30 (0%)

Query 2 GLRNQEESDGMLVFPLFIFGKEGSQDKYNG 31

+ E SD ML+ ++FGK+G +K +

Sbjct 183 QQQQGESSDDMLMPFFWVFGKQGDNNKGDA 212

>g26217.t1

Length=603

Score = 26.2 bits (56), Expect = 0.58, Method: Composition-based stats.

Identities = 12/31 (39%), Positives = 17/31 (55%), Gaps = 0/31 (0%)

Query 1 GGLRNQEESDGMLVFPLFIFGKEGSQDKYNG 31

GG+ +E G+ P+ +FGK G QD G

Sbjct 111 GGMNVEEGDAGLFPIPMTVFGKGGDQDVEKG 141

>g3343.t1

Length=260

Score = 23.5 bits (49), Expect = 5.4, Method: Composition-based stats.

Identities = 7/23 (30%), Positives = 13/23 (57%), Gaps = 0/23 (0%)

Query 4 RNQEESDGMLVFPLFIFGKEGSQ 26

+ EE + +F ++FGK G +

Sbjct 78 KGHEEEEDASLFNYYVFGKAGQE 100

Score = 23.1 bits (48), Expect = 7.9, Method: Composition-based stats.

Identities = 7/24 (29%), Positives = 13/24 (54%), Gaps = 0/24 (0%)

Query 4 RNQEESDGMLVFPLFIFGKEGSQD 27

+ EE +F ++FGK G ++

Sbjct 132 KGHEEEGDASLFNYYVFGKAGQEE 155

Score = 23.1 bits (48), Expect = 9.0, Method: Composition-based stats.

Identities = 7/24 (29%), Positives = 13/24 (54%), Gaps = 0/24 (0%)

Query 4 RNQEESDGMLVFPLFIFGKEGSQD 27

+ EE +F ++FGK G ++

Sbjct 222 KGHEEEADASLFNYYVFGKAGQEE 245

Lambda K H a alpha

0.316 0.130 0.330 0.792 4.96

Gapped

Lambda K H a alpha sigma

0.267 0.0399 0.140 1.90 42.6 43.6

Effective search space used: 532628800

Search has CONVERGED!

Database: LuBethune_augustus-hints.aa

Posted date: Dec 2, 2020 8:14 PM

Number of letters in database: 21,786,600

Number of sequences in database: 60,181

Matrix: BLOSUM62

Gap Penalties: Existence: 11, Extension: 1

Neighboring words threshold: 11

Window for multiple hits: 40
