# Supplementary material for: The flax genome reveals orbitide diversity
Source: BMC Genomics. 2022 Jul 23;23:534. doi: 10.1186/s12864-022-08735-x (PMC9308333; doi:10.1186/s12864-022-08735-x)
Supplement: Supplementary file 5 — Additional file 5: Data S4. Repeat HMM search output. [file 12864_2022_8735_MOESM5_ESM.docx]

Summary of proteins containing sequences matching the profile HMM from 3 rounds of HMM search

| Protein ID | Significant hits (!) | | | Updated name |
| --- | --- | --- | --- | --- |
|  | Round 1 | Round 2 | Round 3 |  |
| g7437.t1 | 5 | 5 | 5 | G14-170N |
| g24919.t1 | 3 | 3 | 3 | G11-516P |
| g33422.t1 | 3 | 3 | 3 | G4-136N |
| g53356.t1 | 6 | 6 | 6 | G3-449N |
| g3343.t1 | 3 | 8 | 9 | Lu8-3343 |
| g26217.t1 | 3 | 3 | 3 | Lu11-26217 |
| g28070.t1 | 2 | 3 | 3 | Lu11-28070 |
| g34966.t1 | 3 | 15 | 14 | Lu10-34966 |
| g46938.t1 | 2 | 4 | 6 | Lu5-46938 |
| g2811.t1 |  | 2 | 4 | Lu8-2811 |
| g15288.t1 |  | 0 | 0 | N/A |
| g18055.t1 |  | 2 | 10 | Lu1-18055 |
| g24918.t1 |  | 2 | 9 | Lu11-24918 |

Note: the first 4 are known linusorb precursor proteins.

Round 1 HMM search output:

Query: Alignment-of-linusorb-repeats [M=32]

Scores for complete sequences (score includes all domains):

--- full sequence --- --- best 1 domain --- -#dom-

E-value score bias E-value score bias exp N Sequence Description

------- ------ ----- ------- ------ ----- ---- -- -------- -----------

3.5e-54 182.0 43.1 1.7e-11 45.4 1.8 5.5 6 g7437.t1

2.2e-53 179.4 10.0 5.3e-18 66.3 0.1 3.8 4 g24919.t1

5.9e-34 117.3 13.9 2.5e-10 41.7 0.9 6.6 7 g53356.t1

5.1e-29 101.5 2.5 2.9e-14 54.3 0.0 3.2 3 g33422.t1

3e-07 31.8 7.9 0.0098 17.4 0.8 3.4 3 g26217.t1

1.2e-05 26.7 7.3 0.0022 19.5 0.3 4.9 4 g28070.t1

8.7e-05 24.0 15.5 8.7 8.0 0.1 7.5 8 g3343.t1

0.0018 19.8 0.3 0.25 12.9 0.0 6.1 6 g46938.t1

0.0022 19.5 20.7 6.5 8.4 0.0 9.4 10 g34966.t1

------ inclusion threshold ------

0.058 14.9 1.3 0.12 13.9 0.5 2.0 1 g39692.t1

0.21 13.1 20.1 3e+02 3.1 0.7 8.6 9 g56299.t1

The top 9 proteins are above the inclusion threshold. The followings are hits between the subject sequences and the profile HMM. Only those marked with “!” were determined as significant hits. Subject sequences of the significant hits were extracted and aligned to the original alignment using *hmmalign* for the next round of HMM search.

Domain annotation for each sequence (and alignments):

>> g7437.t1

# score bias c-Evalue i-Evalue hmmfrom hmm to alifrom ali to envfrom env to acc

--- ------ ----- --------- --------- ------- ------- ------- ------- ------- ------- ----

1 ? -3.4 0.0 5.6 3.1e+04 19 22 .. 75 78 .. 75 79 .. 0.80

2 ! 40.2 2.6 1.3e-13 7.3e-10 8 31 .. 98 118 .. 91 119 .. 0.87

3 ! 45.4 1.8 3.1e-15 1.7e-11 6 31 .. 120 142 .. 117 143 .. 0.91

4 ! 42.8 2.0 2.1e-14 1.1e-10 5 31 .. 142 165 .. 140 166 .. 0.91

5 ! 41.9 3.4 3.8e-14 2.1e-10 5 27 .. 165 186 .. 163 188 .. 0.93

6 ! 39.5 1.6 2.3e-13 1.2e-09 6 27 .. 188 208 .. 186 211 .. 0.92

Alignments for each domain:

== domain 1 score: -3.4 bits; conditional E-value: 5.6

Alignment-of-linusorb-repeats 19 iFGK 22

+FGK

g7437.t1 75 VFGK 78

6**9 PP

== domain 2 score: 40.2 bits; conditional E-value: 1.3e-13

Alignment-of-linusorb-repeats 8 gDdmLvPfFFwiFGKegsqdYNea 31

DdmL+P FFwiFGKeg+q+ ea

g7437.t1 98 ADDMLMP-FFWIFGKEGQQQ--EA 118

6******.***********9..66 PP

== domain 3 score: 45.4 bits; conditional E-value: 3.1e-15

Alignment-of-linusorb-repeats 6 EegDdmLvPfFFwiFGKegsqdYNea 31

E +DdmL+P FFwiFGKeg+q+ ea

g7437.t1 120 ESSDDMLMP-FFWIFGKEGQQQ--EA 142

89*******.***********9..66 PP

== domain 4 score: 42.8 bits; conditional E-value: 2.1e-14

Alignment-of-linusorb-repeats 5 qEegDdmLvPfFFwiFGKegsqdYNea 31

E +DdmL P FFwiFGKeg+q+ ea

g7437.t1 142 AESSDDMLLP-FFWIFGKEGQQQ--EA 165

599*******.***********9..66 PP

== domain 5 score: 41.9 bits; conditional E-value: 3.8e-14

Alignment-of-linusorb-repeats 5 qEegDdmLvPfFFwiFGKegsqd 27

E +DdmL+P FFwiFGK+++q+

g7437.t1 165 AESSDDMLMP-FFWIFGKQQQQQ 186

599*******.***********9 PP

== domain 6 score: 39.5 bits; conditional E-value: 2.3e-13

Alignment-of-linusorb-repeats 6 EegDdmLvPfFFwiFGKegsqd 27

E +DdmL+P FFw+FGK+g+++

g7437.t1 188 ESSDDMLMP-FFWVFGKQGDNN 208

88*******.**********98 PP

>> g24919.t1

# score bias c-Evalue i-Evalue hmmfrom hmm to alifrom ali to envfrom env to acc

--- ------ ----- --------- --------- ------- ------- ------- ------- ------- ------- ----

1 ! 55.1 0.4 2.9e-18 1.6e-14 2 32 .] 82 112 .. 81 112 .. 0.96

2 ! 66.3 0.1 9.6e-22 5.3e-18 2 32 .] 114 148 .. 113 148 .. 0.97

3 ! 62.9 0.0 1.1e-20 6e-17 2 32 .] 153 184 .. 152 184 .. 0.96

4 ? -1.3 0.2 1.2 6.8e+03 21 27 .. 190 196 .. 188 198 .. 0.81

Alignments for each domain:

== domain 1 score: 55.1 bits; conditional E-value: 2.9e-18

Alignment-of-linusorb-repeats 2 vrdqEegDdmLvPfFFwiFGKegsqd.YNeaa 32

+r+qEe+D+mLv+++F iFGKegsqd YN+aa

g24919.t1 82 LRNQEESDGMLVFPLF-IFGKEGSQDkYNGAA 112

89**************.*************97 PP

== domain 2 score: 66.3 bits; conditional E-value: 9.6e-22

Alignment-of-linusorb-repeats 2 vrdqEegDdmLvPfFFwiFGKegsqd....YNeaa 32

+rdqEe+D+mL+P+FF+iFGKeg+qd YN+aa

g24919.t1 114 LRDQEESDGMLIPPFFVIFGKEGCQDighkYNNAA 148

89*******************************97 PP

== domain 3 score: 62.9 bits; conditional E-value: 1.1e-20

Alignment-of-linusorb-repeats 2 vrdqEegDdmLvPfFFwiFGKegsqd.YNeaa 32

+rdqEe+D++LvP+FF+iFGKegsqd YN+aa

g24919.t1 153 LRDQEESDGILVPPFFLIFGKEGSQDkYNAAA 184

89***************************997 PP

== domain 4 score: -1.3 bits; conditional E-value: 1.2

Alignment-of-linusorb-repeats 21 GKegsqd 27

GKe++ d

g24919.t1 190 GKEQQGD 196

9998865 PP

>> g53356.t1

# score bias c-Evalue i-Evalue hmmfrom hmm to alifrom ali to envfrom env to acc

--- ------ ----- --------- --------- ------- ------- ------- ------- ------- ------- ----

1 ! 41.7 0.9 4.7e-14 2.5e-10 6 30 .. 68 92 .. 65 92 .. 0.92

2 ! 18.5 0.0 8.1e-07 0.0044 2 23 .. 89 110 .. 89 111 .. 0.92

3 ! 15.7 0.0 6.1e-06 0.033 2 23 .. 113 134 .. 112 135 .. 0.91

4 ! 20.1 0.0 2.5e-07 0.0014 2 23 .. 137 158 .. 136 163 .. 0.88

5 ! 15.2 0.1 9.1e-06 0.05 2 23 .. 161 182 .. 160 187 .. 0.86

6 ! 16.3 0.0 4e-06 0.022 2 31 .. 185 213 .. 184 214 .. 0.83

7 ? -0.2 0.0 0.56 3e+03 2 14 .. 209 221 .. 208 221 .. 0.81

Alignments for each domain:

== domain 1 score: 41.7 bits; conditional E-value: 4.7e-14

Alignment-of-linusorb-repeats 6 EegDdmLvPfFFwiFGKegsqdYNe 30

+ gDd+++Pf++++FGK++++++N+

g53356.t1 68 GIGDDGIPPFWLTLFGKQQANVFNS 92

569*********************5 PP

== domain 2 score: 18.5 bits; conditional E-value: 8.1e-07

Alignment-of-linusorb-repeats 2 vrdqEegDdmLvPfFFwiFGKe 23

v+++E+gD+++ P + ++FG e

g53356.t1 89 VFNSEKGDAGMAPMWVTVFGSE 110

79******************55 PP

== domain 3 score: 15.7 bits; conditional E-value: 6.1e-06

Alignment-of-linusorb-repeats 2 vrdqEegDdmLvPfFFwiFGKe 23

v+++E+gD+++ P + ++FG e

g53356.t1 113 VFNSEKGDAGMAPVWGTVFGSE 134

89******************55 PP

== domain 4 score: 20.1 bits; conditional E-value: 2.5e-07

Alignment-of-linusorb-repeats 2 vrdqEegDdmLvPfFFwiFGKe 23

v+++E+gD+++ P + ++FG e

g53356.t1 137 VFNSEKGDAGMAPMWVTVFGSE 158

89******************66 PP

== domain 5 score: 15.2 bits; conditional E-value: 9.1e-06

Alignment-of-linusorb-repeats 2 vrdqEegDdmLvPfFFwiFGKe 23

v++ E+gD+++ P + ++FG e

g53356.t1 161 VFNLEKGDAGMAPMWVTVFGSE 182

7899****************66 PP

== domain 6 score: 16.3 bits; conditional E-value: 4e-06

Alignment-of-linusorb-repeats 2 vrdqEegDdmLvPfFFwiFGKegsqdYNea 31

v++ E+gD+++ P + ++FG e ++N++

g53356.t1 185 VFNLEKGDAGMAPVWVTVFGSE-RGVFNSK 213

7899****************66.5557765 PP

== domain 7 score: -0.2 bits; conditional E-value: 0.56

Alignment-of-linusorb-repeats 2 vrdqEegDdmLvP 14

v+++ +gD+ + P

g53356.t1 209 VFNSKKGDASMAP 221

899*****97766 PP

>> g33422.t1

# score bias c-Evalue i-Evalue hmmfrom hmm to alifrom ali to envfrom env to acc

--- ------ ----- --------- --------- ------- ------- ------- ------- ------- ------- ----

1 ! 54.3 0.0 5.2e-18 2.9e-14 1 31 [. 53 83 .. 53 84 .. 0.96

2 ! 8.5 0.8 0.0011 5.8 8 21 .. 85 98 .. 82 98 .. 0.92

3 ! 40.8 0.1 9e-14 4.9e-10 1 24 [. 95 118 .. 95 121 .. 0.92

Alignments for each domain:

== domain 1 score: 54.3 bits; conditional E-value: 5.2e-18

Alignment-of-linusorb-repeats 1 evrdqEegDdmLvPfFFwiFGKegsqdYNea 31

ev+d+E+gD++++Pf+++++GK++++++N++

g33422.t1 53 EVVDSERGDAGIPPFWLTLVGKQRTDVFNSK 83

79***************************97 PP

== domain 2 score: 8.5 bits; conditional E-value: 0.0011

Alignment-of-linusorb-repeats 8 gDdmLvPfFFwiFG 21

gD++L+P + +FG

g33422.t1 85 GDAGLPPMWVEVFG 98

9********99999 PP

== domain 3 score: 40.8 bits; conditional E-value: 9e-14

Alignment-of-linusorb-repeats 1 evrdqEegDdmLvPfFFwiFGKeg 24

ev+++E+gD++++Pf+++++GK+

g33422.t1 95 EVFGSERGDAGIPPFWLTLIGKHA 118

79********************95 PP

>> g26217.t1

# score bias c-Evalue i-Evalue hmmfrom hmm to alifrom ali to envfrom env to acc

--- ------ ----- --------- --------- ------- ------- ------- ------- ------- ------- ----

1 ! 7.5 0.0 0.0023 12 5 23 .. 55 72 .. 51 78 .. 0.83

2 ! 10.5 0.4 0.00026 1.4 3 27 .. 91 115 .. 90 116 .. 0.92

3 ! 17.4 0.8 1.8e-06 0.0098 5 27 .. 115 137 .. 113 140 .. 0.94

Alignments for each domain:

== domain 1 score: 7.5 bits; conditional E-value: 0.0023

Alignment-of-linusorb-repeats 5 qEegDdmLvPfFFwiFGKe 23

E gD+m+ P +FGK

g26217.t1 55 REHGDAMIFP-ITRLFGKL 72

699*******.77799995 PP

== domain 2 score: 10.5 bits; conditional E-value: 0.00026

Alignment-of-linusorb-repeats 3 rdqEegDdmLvPfFFwiFGKegsqd 27

++ Ee D++L P ++FGK g ++

g26217.t1 91 FGEEEDDAGLFPIPMTVFGKGGMNV 115

588*****************99887 PP

== domain 3 score: 17.4 bits; conditional E-value: 1.8e-06

Alignment-of-linusorb-repeats 5 qEegDdmLvPfFFwiFGKegsqd 27

EegD++L P ++FGK g+qd

g26217.t1 115 VEEGDAGLFPIPMTVFGKGGDQD 137

69********************9 PP

>> g28070.t1

# score bias c-Evalue i-Evalue hmmfrom hmm to alifrom ali to envfrom env to acc

--- ------ ----- --------- --------- ------- ------- ------- ------- ------- ------- ----

1 ! 5.9 0.2 0.0069 38 3 26 .. 15 33 .. 13 40 .. 0.80

2 ! 19.5 0.3 4.1e-07 0.0022 2 30 .. 46 74 .. 45 76 .. 0.92

3 ? -2.6 0.0 3.2 1.8e+04 19 23 .. 99 103 .. 99 104 .. 0.83

4 ? -2.1 0.0 2.2 1.2e+04 19 23 .. 123 127 .. 123 131 .. 0.82

Alignments for each domain:

== domain 1 score: 5.9 bits; conditional E-value: 0.0069

Alignment-of-linusorb-repeats 3 rdqEegDdmLvPfFFwiFGKegsq 26

r+q gD++ +P + +FG q

g28070.t1 15 RNQDNGDAGYPP-LSPLFG----Q 33

79*******999.777888....3 PP

== domain 2 score: 19.5 bits; conditional E-value: 4.1e-07

Alignment-of-linusorb-repeats 2 vrdqEegDdmLvPfFFwiFGKegsqd.YNe 30

+++qE+gD++ +P + +FGKe+ + YN+

g28070.t1 46 LFGQEKGDAGYPP-LSPLFGKEKGDAgYNG 74

69********999.999*****99999995 PP

== domain 3 score: -2.6 bits; conditional E-value: 3.2

Alignment-of-linusorb-repeats 19 iFGKe 23

+FGK

g28070.t1 99 LFGKD 103

7**95 PP

== domain 4 score: -2.1 bits; conditional E-value: 2.2

Alignment-of-linusorb-repeats 19 iFGKe 23

+FGK

g28070.t1 123 LFGKD 127

7**97 PP

>> g3343.t1

# score bias c-Evalue i-Evalue hmmfrom hmm to alifrom ali to envfrom env to acc

--- ------ ----- --------- --------- ------- ------- ------- ------- ------- ------- ----

1 ? 4.0 0.1 0.029 1.6e+02 18 29 .. 92 108 .. 81 110 .. 0.70

2 ? 3.2 0.4 0.049 2.7e+02 4 22 .. 98 114 .. 95 126 .. 0.75

3 ? 2.2 0.0 0.1 5.5e+02 6 22 .. 118 132 .. 115 135 .. 0.80

4 ! 7.0 0.1 0.0031 17 6 27 .. 136 155 .. 133 162 .. 0.71

5 ! 7.0 0.0 0.0033 18 4 22 .. 152 168 .. 150 171 .. 0.83

6 ! 8.0 0.1 0.0016 8.7 6 29 .. 172 198 .. 169 199 .. 0.79

7 ? 3.0 0.4 0.058 3.1e+02 6 26 .. 226 244 .. 206 246 .. 0.78

8 ? 2.1 0.4 0.11 5.8e+02 4 10 .. 242 248 .. 239 249 .. 0.87

Alignments for each domain:

== domain 1 score: 4.0 bits; conditional E-value: 0.029

Alignment-of-linusorb-repeats 18 wiFGKegsqd.....YN 29

+FGK g+ + +N

g3343.t1 92 YVFGKAGQEVgdaslFN 108

48999999885555555 PP

== domain 2 score: 3.2 bits; conditional E-value: 0.049

Alignment-of-linusorb-repeats 4 dqEegDdmLvPfFFwiFGK 22

+qE gD+ L + +FGK

g3343.t1 98 GQEVGDASLFN-YY-VFGK 114

89999999977.44.8998 PP

== domain 3 score: 2.2 bits; conditional E-value: 0.1

Alignment-of-linusorb-repeats 6 EegDdmLvPfFFwiFGK 22

EegD+ L + +FGK

g3343.t1 118 EEGDASLFN-YY-VFGK 132

99***9977.44.8998 PP

== domain 4 score: 7.0 bits; conditional E-value: 0.0031

Alignment-of-linusorb-repeats 6 EegDdmLvPfFFwiFGKegsqd 27

EegD+ L + +FGK g+

g3343.t1 136 EEGDASLFN-Y-YVFGKAGQEE 155

888888877.4.4899988876 PP

== domain 5 score: 7.0 bits; conditional E-value: 0.0033

Alignment-of-linusorb-repeats 4 dqEegDdmLvPfFFwiFGK 22

+qEegD+ L + +FGK

g3343.t1 152 GQEEGDASLFN-YY-VFGK 168

8*******987.44.8998 PP

== domain 6 score: 8.0 bits; conditional E-value: 0.0016

Alignment-of-linusorb-repeats 6 EegDdmLvPfFFwiFGKegsqd.....YN 29

EegD+ L + +FGK g+ + +N

g3343.t1 172 EEGDASLFN-Y-YVFGKAGQEVddaslFN 198

99*****88.4.49******985566665 PP

== domain 7 score: 3.0 bits; conditional E-value: 0.058

Alignment-of-linusorb-repeats 6 EegDdmLvPfFFwiFGKegsq 26

Ee D+ L +FGK g+

g3343.t1 226 EEADASLFN--YYVFGKAGQE 244

666666655..3489998885 PP

== domain 8 score: 2.1 bits; conditional E-value: 0.11

Alignment-of-linusorb-repeats 4 dqEegDd 10

+qEegD+

g3343.t1 242 GQEEGDA 248

9*****7 PP

>> g46938.t1

# score bias c-Evalue i-Evalue hmmfrom hmm to alifrom ali to envfrom env to acc

--- ------ ----- --------- --------- ------- ------- ------- ------- ------- ------- ----

1 ? -0.6 0.1 0.78 4.3e+03 9 14 .. 23 28 .. 21 33 .. 0.86

2 ! 12.9 0.0 4.6e-05 0.25 7 22 .. 42 57 .. 36 59 .. 0.90

3 ! 11.4 0.1 0.00013 0.72 8 23 .. 65 79 .. 63 84 .. 0.90

4 ? 5.1 0.3 0.013 70 14 23 .. 85 94 .. 80 96 .. 0.72

5 ? -3.0 4.6 4.2 2.3e+04 9 22 .. 102 114 .. 100 116 .. 0.53

6 ? 2.5 0.7 0.08 4.4e+02 16 23 .. 130 137 .. 124 139 .. 0.73

Alignments for each domain:

== domain 1 score: -0.6 bits; conditional E-value: 0.78

Alignment-of-linusorb-repeats 9 DdmLvP 14

D++L+P

g46938.t1 23 DAGLIP 28

99***9 PP

== domain 2 score: 12.9 bits; conditional E-value: 4.6e-05

Alignment-of-linusorb-repeats 7 egDdmLvPfFFwiFGK 22

egD++++ + ++iFGK

g46938.t1 42 EGDAGIIDPILLIFGK 57

9**************9 PP

== domain 3 score: 11.4 bits; conditional E-value: 0.00013

Alignment-of-linusorb-repeats 8 gDdmLvPfFFwiFGKe 23

gD++ vP F +FGKe

g46938.t1 65 GDAGFVP-NFSVFGKE 79

9******.999****9 PP

== domain 4 score: 5.1 bits; conditional E-value: 0.013

Alignment-of-linusorb-repeats 14 PfFFwiFGKe 23

+ F iFGKe

g46938.t1 85 IPSFPIFGKE 94

45799****9 PP

== domain 5 score: -3.0 bits; conditional E-value: 4.2

Alignment-of-linusorb-repeats 9 DdmLvPfFFwiFGK 22

D++ FF FGK

g46938.t1 102 DAGFDI-FFPFFGK 114

555333.4444587 PP

== domain 6 score: 2.5 bits; conditional E-value: 0.08

Alignment-of-linusorb-repeats 16 FFwiFGKe 23

FF FGK+

g46938.t1 130 FFPFFGKQ 137

45556*99 PP

>> g34966.t1

# score bias c-Evalue i-Evalue hmmfrom hmm to alifrom ali to envfrom env to acc

--- ------ ----- --------- --------- ------- ------- ------- ------- ------- ------- ----

1 ? -0.4 0.2 0.68 3.7e+03 9 27 .. 37 54 .. 35 56 .. 0.71

2 ? -2.6 0.0 3.3 1.8e+04 4 22 .. 64 81 .. 61 88 .. 0.53

3 ? 2.0 0.1 0.12 6.5e+02 2 26 .. 110 133 .. 109 136 .. 0.82

4 ? 3.3 0.1 0.047 2.6e+02 2 26 .. 142 161 .. 141 166 .. 0.80

5 ! 5.8 0.2 0.0079 43 2 26 .. 158 181 .. 157 184 .. 0.81

6 ! 7.9 0.1 0.0017 9.2 2 23 .. 174 194 .. 173 200 .. 0.82

7 ? 3.9 0.2 0.031 1.7e+02 2 21 .. 190 208 .. 189 217 .. 0.83

8 ! 8.4 0.0 0.0012 6.5 2 25 .. 222 244 .. 221 248 .. 0.82

9 ? 1.9 0.1 0.13 7.1e+02 2 21 .. 238 256 .. 237 264 .. 0.80

10 ? -0.7 0.0 0.8 4.4e+03 5 21 .. 274 291 .. 273 294 .. 0.75

Alignments for each domain:

== domain 1 score: -0.4 bits; conditional E-value: 0.68

Alignment-of-linusorb-repeats 9 DdmLvPfFFwiFGKegsqd 27

D++ +P + +FGK + ++

g34966.t1 37 DGGYPP-LSPLFGKKKWDV 54

555555.7779***97766 PP

== domain 2 score: -2.6 bits; conditional E-value: 3.3

Alignment-of-linusorb-repeats 4 dqEegDdmLvPfFFwiFGK 22

+q + D+ +P + +FG

g34966.t1 64 GQKKNDASYPP-LSSLFGR 81

44444554444.6566665 PP

== domain 3 score: 2.0 bits; conditional E-value: 0.12

Alignment-of-linusorb-repeats 2 vrdqEegDdmLvPfFFwiFGKegsq 26

+++q +gD + +P + +FG e+++

g34966.t1 110 LFGQKKGDTGYPP-LSPLFGQEKDN 133

68999*****999.88899977766 PP

== domain 4 score: 3.3 bits; conditional E-value: 0.047

Alignment-of-linusorb-repeats 2 vrdqEegDdmLvPfFFwiFGKegsq 26

+++q ++D++ +P + +FG q

g34966.t1 142 LFGQKKSDAGYPP-LSPLFG----Q 161

789*******999.777888....3 PP

== domain 5 score: 5.8 bits; conditional E-value: 0.0079

Alignment-of-linusorb-repeats 2 vrdqEegDdmLvPfFFwiFGKegsq 26

+++q gD++ +P + +FG e+s+

g34966.t1 158 LFGQDNGDAGYPP-LSPLFGQEKSD 181

6899999999999.88899988776 PP

== domain 6 score: 7.9 bits; conditional E-value: 0.0017

Alignment-of-linusorb-repeats 2 vrdqEegDdmLvPfFFwiFGKe 23

+++qE++D++ +P + +FG e

g34966.t1 174 LFGQEKSDAGYPP-LSPLFGQE 194

69********999.87788855 PP

== domain 7 score: 3.9 bits; conditional E-value: 0.031

Alignment-of-linusorb-repeats 2 vrdqEegDdmLvPfFFwiFG 21

+++qEe D++ +P + +FG

g34966.t1 190 LFGQEERDAGYPP-LSPLFG 208

69********999.767777 PP

== domain 8 score: 8.4 bits; conditional E-value: 0.0012

Alignment-of-linusorb-repeats 2 vrdqEegDdmLvPfFFwiFGKegs 25

+++qE++D++ +P + +FG e+

g34966.t1 222 LFGQEKSDAGYPP-LSPLFGQEKR 244

69********999.8889997655 PP

== domain 9 score: 1.9 bits; conditional E-value: 0.13

Alignment-of-linusorb-repeats 2 vrdqEegDdmLvPfFFwiFG 21

+++qE+ D++ +P + +FG

g34966.t1 238 LFGQEKRDAGYPP-LSPLFG 256

689*******999.777777 PP

== domain 10 score: -0.7 bits; conditional E-value: 0.8

Alignment-of-linusorb-repeats 5 qEegDdmLv.PfFFwiFG 21

q egD++ v P F +iFG

g34966.t1 274 QPEGDAGCVsPDFHMIFG 291

568999876366889999 PP

Round 2 HMM search output:

Query: round-1-all-seq-for-round-2-input-by-hmmalign [M=20]

Scores for complete sequences (score includes all domains):

--- full sequence --- --- best 1 domain --- -#dom-

E-value score bias E-value score bias exp N Sequence Description

------- ------ ----- ------- ------ ----- ---- -- -------- -----------

1.9e-71 235.7 121.5 4.7e-06 28.2 0.1 23.9 26 g34966.t1

3.6e-46 155.5 34.3 8.8e-09 36.8 0.9 7.0 7 g7437.t1

6.3e-46 154.7 31.7 1.1e-05 27.0 0.0 10.4 11 g3343.t1

8.8e-22 78.1 11.0 1.8e-05 26.3 0.3 7.3 8 g53356.t1

6.5e-21 75.3 22.7 3.9e-08 34.8 0.3 4.2 5 g24919.t1

4.2e-20 72.8 7.9 1.4e-06 29.9 0.7 3.9 3 g26217.t1

1.8e-18 67.6 38.9 2.4 10.1 0.0 20.8 25 g18055.t1

3.3e-18 66.7 16.2 9.7e-08 33.5 2.5 9.2 8 g28070.t1

6.2e-18 65.9 3.4 4.8e-06 28.2 0.0 3.7 4 g33422.t1

2.1e-14 54.7 16.0 1.8e+02 4.1 0.0 15.1 16 g15288.t1

8.1e-12 46.5 41.9 0.00049 21.8 0.2 7.2 7 g46938.t1

8.9e-11 43.2 4.7 1.6e-05 26.5 0.1 11.9 9 g24918.t1

1.6e-05 26.6 22.1 0.05 15.4 0.1 4.6 4 g2811.t1

------ inclusion threshold ------

0.071 14.9 42.1 1.1e+02 4.9 0.0 14.0 19 g23576.t1

4.1 9.4 26.0 15 7.6 0.0 8.7 9 g56299.t1

Domain annotation for each sequence (and alignments):

>> g34966.t1

# score bias c-Evalue i-Evalue hmmfrom hmm to alifrom ali to envfrom env to acc

--- ------ ----- --------- --------- ------- ------- ------- ------- ------- ------- ----

1 ! 24.1 1.0 2.3e-08 9.4e-05 2 20 .] 35 53 .. 34 53 .. 0.91

2 ! 13.3 0.9 5.9e-05 0.24 6 16 .. 55 65 .. 53 68 .. 0.87

3 ! 16.0 0.1 8.2e-06 0.033 4 15 .. 69 80 .. 66 83 .. 0.93

4 ! 20.8 0.3 2.4e-07 0.00098 4 16 .. 101 113 .. 98 115 .. 0.88

5 ! 26.3 0.2 4.7e-09 1.9e-05 2 20 .] 115 133 .. 114 133 .. 0.94

6 ! 8.7 0.2 0.0016 6.5 6 18 .. 135 147 .. 134 148 .. 0.87

7 ! 23.7 0.4 3e-08 0.00012 2 16 .. 147 161 .. 146 163 .. 0.92

8 ! 27.6 0.4 1.8e-09 7.1e-06 2 19 .. 163 180 .. 162 180 .. 0.92

9 ! 26.6 0.3 3.7e-09 1.5e-05 1 17 [. 178 194 .. 178 194 .. 0.95

10 ! 24.4 0.1 1.8e-08 7.1e-05 1 16 [. 194 209 .. 194 210 .. 0.94

11 ! 26.0 0.2 5.7e-09 2.3e-05 2 19 .. 211 228 .. 210 228 .. 0.92

12 ! 28.2 0.1 1.2e-09 4.7e-06 1 18 [. 226 243 .. 226 244 .. 0.95

13 ! 23.3 0.3 4.2e-08 0.00017 1 16 [. 242 257 .. 242 259 .. 0.93

14 ! 12.0 0.2 0.00014 0.58 4 15 .. 261 272 .. 258 274 .. 0.89

15 ! 17.5 0.1 2.8e-06 0.011 1 15 [. 275 291 .. 275 292 .. 0.96

16 ? -0.0 0.1 0.92 3.7e+03 1 7 [. 335 341 .. 335 341 .. 0.87

17 ? -0.0 0.1 0.92 3.7e+03 1 7 [. 352 358 .. 352 358 .. 0.87

18 ? -0.0 0.1 0.92 3.7e+03 1 7 [. 369 375 .. 369 375 .. 0.87

19 ? -0.0 0.1 0.92 3.7e+03 1 7 [. 386 392 .. 386 392 .. 0.87

20 ? -0.0 0.1 0.92 3.7e+03 1 7 [. 403 409 .. 403 409 .. 0.87

21 ? -0.0 0.1 0.92 3.7e+03 1 7 [. 420 426 .. 420 426 .. 0.87

22 ? -0.0 0.1 0.92 3.7e+03 1 7 [. 437 443 .. 437 443 .. 0.87

23 ? -1.3 0.1 2.3 9.4e+03 3 7 .. 456 460 .. 455 460 .. 0.92

24 ? 1.0 0.1 0.43 1.7e+03 1 8 [. 471 478 .. 471 481 .. 0.82

25 ? 1.0 0.1 0.43 1.7e+03 1 8 [. 488 495 .. 488 498 .. 0.82

26 ? -0.0 0.1 0.92 3.7e+03 1 7 [. 505 511 .. 505 511 .. 0.87

Alignments for each domain:

== domain 1 score: 24.1 bits; conditional E-value: 2.3e-08

round-1-all-seq-for-round-2-input-by-hmmalign 2 eGDAGlpPffplFGKeGqq 20

+ D+G+pP++plFGK +++

g34966.t1 35 KRDGGYPPLSPLFGKKKWD 53

67*************9985 PP

== domain 2 score: 13.3 bits; conditional E-value: 5.9e-05

round-1-all-seq-for-round-2-input-by-hmmalign 6 GlpPffplFGK 16

G+pP++plFG+

g34966.t1 55 GYPPLSPLFGQ 65

9*********7 PP

== domain 3 score: 16.0 bits; conditional E-value: 8.2e-06

round-1-all-seq-for-round-2-input-by-hmmalign 4 DAGlpPffplFG 15

DA++pP+++lFG

g34966.t1 69 DASYPPLSSLFG 80

************ PP

== domain 4 score: 20.8 bits; conditional E-value: 2.4e-07

round-1-all-seq-for-round-2-input-by-hmmalign 4 DAGlpPffplFGK 16

DAG+pP++plFG+

g34966.t1 101 DAGYPPLSPLFGQ 113

************7 PP

== domain 5 score: 26.3 bits; conditional E-value: 4.7e-09

round-1-all-seq-for-round-2-input-by-hmmalign 2 eGDAGlpPffplFGKeGqq 20

+GD G+pP++plFG+e+++

g34966.t1 115 KGDTGYPPLSPLFGQEKDN 133

8***************986 PP

== domain 6 score: 8.7 bits; conditional E-value: 0.0016

round-1-all-seq-for-round-2-input-by-hmmalign 6 GlpPffplFGKeG 18

G+ P+++lFG+ +

g34966.t1 135 GYLPLSSLFGQKK 147

9*********865 PP

== domain 7 score: 23.7 bits; conditional E-value: 3e-08

round-1-all-seq-for-round-2-input-by-hmmalign 2 eGDAGlpPffplFGK 16

++DAG+pP++plFG+

g34966.t1 147 KSDAGYPPLSPLFGQ 161

89************6 PP

== domain 8 score: 27.6 bits; conditional E-value: 1.8e-09

round-1-all-seq-for-round-2-input-by-hmmalign 2 eGDAGlpPffplFGKeGq 19

+GDAG+pP++plFG+e++

g34966.t1 163 NGDAGYPPLSPLFGQEKS 180

7**************985 PP

== domain 9 score: 26.6 bits; conditional E-value: 3.7e-09

round-1-all-seq-for-round-2-input-by-hmmalign 1 eeGDAGlpPffplFGKe 17

e++DAG+pP++plFG+e

g34966.t1 178 EKSDAGYPPLSPLFGQE 194

89*************86 PP

== domain 10 score: 24.4 bits; conditional E-value: 1.8e-08

round-1-all-seq-for-round-2-input-by-hmmalign 1 eeGDAGlpPffplFGK 16

ee DAG+pP++plFG+

g34966.t1 194 EERDAGYPPLSPLFGQ 209

89*************6 PP

== domain 11 score: 26.0 bits; conditional E-value: 5.7e-09

round-1-all-seq-for-round-2-input-by-hmmalign 2 eGDAGlpPffplFGKeGq 19

e DAG+pP++plFG+e++

g34966.t1 211 ERDAGYPPLSPLFGQEKS 228

89*************985 PP

== domain 12 score: 28.2 bits; conditional E-value: 1.2e-09

round-1-all-seq-for-round-2-input-by-hmmalign 1 eeGDAGlpPffplFGKeG 18

e++DAG+pP++plFG+e+

g34966.t1 226 EKSDAGYPPLSPLFGQEK 243

89*************996 PP

== domain 13 score: 23.3 bits; conditional E-value: 4.2e-08

round-1-all-seq-for-round-2-input-by-hmmalign 1 eeGDAGlpPffplFGK 16

e+ DAG+pP++plFG+

g34966.t1 242 EKRDAGYPPLSPLFGQ 257

799************6 PP

== domain 14 score: 12.0 bits; conditional E-value: 0.00014

round-1-all-seq-for-round-2-input-by-hmmalign 4 DAGlpPffplFG 15

DAG+pP++pl G

g34966.t1 261 DAGYPPLSPLYG 272

**********88 PP

== domain 15 score: 17.5 bits; conditional E-value: 2.8e-06

round-1-all-seq-for-round-2-input-by-hmmalign 1 eeGDAGlp.P.ffplFG 15

+eGDAG++ P f+++FG

g34966.t1 275 PEGDAGCVsPdFHMIFG 291

69*************** PP

== domain 16 score: -0.0 bits; conditional E-value: 0.92

round-1-all-seq-for-round-2-input-by-hmmalign 1 eeGDAGl 7

e GDAG+

g34966.t1 335 EVGDAGY 341

67****9 PP

== domain 17 score: -0.0 bits; conditional E-value: 0.92

round-1-all-seq-for-round-2-input-by-hmmalign 1 eeGDAGl 7

e GDAG+

g34966.t1 352 EVGDAGY 358

67****9 PP

== domain 18 score: -0.0 bits; conditional E-value: 0.92

round-1-all-seq-for-round-2-input-by-hmmalign 1 eeGDAGl 7

e GDAG+

g34966.t1 369 EVGDAGY 375

67****9 PP

== domain 19 score: -0.0 bits; conditional E-value: 0.92

round-1-all-seq-for-round-2-input-by-hmmalign 1 eeGDAGl 7

e GDAG+

g34966.t1 386 EVGDAGY 392

67****9 PP

== domain 20 score: -0.0 bits; conditional E-value: 0.92

round-1-all-seq-for-round-2-input-by-hmmalign 1 eeGDAGl 7

e GDAG+

g34966.t1 403 EVGDAGY 409

67****9 PP

== domain 21 score: -0.0 bits; conditional E-value: 0.92

round-1-all-seq-for-round-2-input-by-hmmalign 1 eeGDAGl 7

e GDAG+

g34966.t1 420 EVGDAGY 426

67****9 PP

== domain 22 score: -0.0 bits; conditional E-value: 0.92

round-1-all-seq-for-round-2-input-by-hmmalign 1 eeGDAGl 7

e GDAG+

g34966.t1 437 EVGDAGY 443

67****9 PP

== domain 23 score: -1.3 bits; conditional E-value: 2.3

round-1-all-seq-for-round-2-input-by-hmmalign 3 GDAGl 7

GDAG+

g34966.t1 456 GDAGY 460

9***9 PP

== domain 24 score: 1.0 bits; conditional E-value: 0.43

round-1-all-seq-for-round-2-input-by-hmmalign 1 eeGDAGlp 8

e GDAG+

g34966.t1 471 EVGDAGYK 478

67****95 PP

== domain 25 score: 1.0 bits; conditional E-value: 0.43

round-1-all-seq-for-round-2-input-by-hmmalign 1 eeGDAGlp 8

e GDAG+

g34966.t1 488 EVGDAGYK 495

67****95 PP

== domain 26 score: -0.0 bits; conditional E-value: 0.92

round-1-all-seq-for-round-2-input-by-hmmalign 1 eeGDAGl 7

e GDAG+

g34966.t1 505 EVGDAGY 511

67****9 PP

>> g7437.t1

# score bias c-Evalue i-Evalue hmmfrom hmm to alifrom ali to envfrom env to acc

--- ------ ----- --------- --------- ------- ------- ------- ------- ------- ------- ----

1 ? 2.9 0.0 0.11 4.5e+02 1 8 [. 62 69 .. 62 69 .. 0.89

2 ? -0.4 0.1 1.2 5e+03 13 16 .. 75 78 .. 75 79 .. 0.85

3 ! 33.8 1.3 2e-11 8e-08 4 20 .] 99 115 .. 98 115 .. 0.97

4 ! 36.8 0.9 2.2e-12 8.8e-09 2 20 .] 121 139 .. 120 139 .. 0.94

5 ! 33.7 0.7 2.1e-11 8.4e-08 2 20 .] 144 162 .. 143 162 .. 0.94

6 ! 34.6 0.7 1.1e-11 4.6e-08 2 20 .] 167 185 .. 166 185 .. 0.93

7 ! 34.1 0.5 1.7e-11 6.7e-08 2 20 .] 189 207 .. 188 207 .. 0.92

Alignments for each domain:

== domain 1 score: 2.9 bits; conditional E-value: 0.11

round-1-all-seq-for-round-2-input-by-hmmalign 1 eeGDAGlp 8

++GDA+l+

g7437.t1 62 PKGDASLF 69

69*****8 PP

== domain 2 score: -0.4 bits; conditional E-value: 1.2

round-1-all-seq-for-round-2-input-by-hmmalign 13 lFGK 16

+FGK

g7437.t1 75 VFGK 78

8**9 PP

== domain 3 score: 33.8 bits; conditional E-value: 2e-11

round-1-all-seq-for-round-2-input-by-hmmalign 4 DAGlpPffplFGKeGqq 20

D++l+Pff++FGKeGqq

g7437.t1 99 DDMLMPFFWIFGKEGQQ 115

9***************8 PP

== domain 4 score: 36.8 bits; conditional E-value: 2.2e-12

round-1-all-seq-for-round-2-input-by-hmmalign 2 eGDAGlpPffplFGKeGqq 20

+D++l+Pff++FGKeGqq

g7437.t1 121 SSDDMLMPFFWIFGKEGQQ 139

59****************8 PP

== domain 5 score: 33.7 bits; conditional E-value: 2.1e-11

round-1-all-seq-for-round-2-input-by-hmmalign 2 eGDAGlpPffplFGKeGqq 20

+D++l Pff++FGKeGqq

g7437.t1 144 SSDDMLLPFFWIFGKEGQQ 162

59****************8 PP

== domain 6 score: 34.6 bits; conditional E-value: 1.1e-11

round-1-all-seq-for-round-2-input-by-hmmalign 2 eGDAGlpPffplFGKeGqq 20

+D++l+Pff++FGK++qq

g7437.t1 167 SSDDMLMPFFWIFGKQQQQ 185

59****************8 PP

== domain 7 score: 34.1 bits; conditional E-value: 1.7e-11

round-1-all-seq-for-round-2-input-by-hmmalign 2 eGDAGlpPffplFGKeGqq 20

+D++l+Pff++FGK+G++

g7437.t1 189 SSDDMLMPFFWVFGKQGDN 207

59***************97 PP

>> g3343.t1

# score bias c-Evalue i-Evalue hmmfrom hmm to alifrom ali to envfrom env to acc

--- ------ ----- --------- --------- ------- ------- ------- ------- ------- ------- ----

1 ? -3.0 0.1 8.2 3.3e+04 2 6 .. 65 69 .. 65 69 .. 0.84

2 ! 20.7 0.0 2.6e-07 0.001 1 20 [] 82 100 .. 82 100 .. 0.87

3 ! 16.1 0.1 7.5e-06 0.03 1 16 [. 100 114 .. 100 116 .. 0.87

4 ! 19.8 0.0 5e-07 0.002 1 16 [. 118 132 .. 118 134 .. 0.90

5 ! 27.0 0.0 2.7e-09 1.1e-05 1 20 [] 136 154 .. 136 154 .. 0.92

6 ! 19.8 0.1 5.2e-07 0.0021 1 16 [. 154 168 .. 154 170 .. 0.90

7 ! 27.0 0.1 2.7e-09 1.1e-05 1 20 [] 172 190 .. 172 190 .. 0.92

8 ? 4.9 0.1 0.027 1.1e+02 3 14 .. 192 202 .. 190 204 .. 0.81

9 ! 15.5 0.0 1.2e-05 0.049 1 16 [. 208 222 .. 208 224 .. 0.89

10 ! 22.6 0.0 6.6e-08 0.00026 1 20 [] 226 244 .. 226 244 .. 0.91

11 ? 0.5 0.1 0.61 2.5e+03 1 6 [. 244 249 .. 244 249 .. 0.90

Alignments for each domain:

== domain 1 score: -3.0 bits; conditional E-value: 8.2

round-1-all-seq-for-round-2-input-by-hmmalign 2 eGDAG 6

+GDA+

g3343.t1 65 KGDAS 69

79*96 PP

== domain 2 score: 20.7 bits; conditional E-value: 2.6e-07

round-1-all-seq-for-round-2-input-by-hmmalign 1 eeGDAGlpPffplFGKeGqq 20

ee DA+l+++ ++FGK+Gq+

g3343.t1 82 EEEDASLFNY-YVFGKAGQE 100

566******7.4*******7 PP

== domain 3 score: 16.1 bits; conditional E-value: 7.5e-06

round-1-all-seq-for-round-2-input-by-hmmalign 1 eeGDAGlpPffplFGK 16

e GDA+l+++ ++FGK

g3343.t1 100 EVGDASLFNY-YVFGK 114

67*******7.4***9 PP

== domain 4 score: 19.8 bits; conditional E-value: 5e-07

round-1-all-seq-for-round-2-input-by-hmmalign 1 eeGDAGlpPffplFGK 16

eeGDA+l+++ ++FGK

g3343.t1 118 EEGDASLFNY-YVFGK 132

8********7.4***9 PP

== domain 5 score: 27.0 bits; conditional E-value: 2.7e-09

round-1-all-seq-for-round-2-input-by-hmmalign 1 eeGDAGlpPffplFGKeGqq 20

eeGDA+l+++ ++FGK+Gq+

g3343.t1 136 EEGDASLFNY-YVFGKAGQE 154

8********7.4*******7 PP

== domain 6 score: 19.8 bits; conditional E-value: 5.2e-07

round-1-all-seq-for-round-2-input-by-hmmalign 1 eeGDAGlpPffplFGK 16

eeGDA+l+++ ++FGK

g3343.t1 154 EEGDASLFNY-YVFGK 168

8********7.4***9 PP

== domain 7 score: 27.0 bits; conditional E-value: 2.7e-09

round-1-all-seq-for-round-2-input-by-hmmalign 1 eeGDAGlpPffplFGKeGqq 20

eeGDA+l+++ ++FGK+Gq+

g3343.t1 172 EEGDASLFNY-YVFGKAGQE 190

8********7.4*******7 PP

== domain 8 score: 4.9 bits; conditional E-value: 0.027

round-1-all-seq-for-round-2-input-by-hmmalign 3 GDAGlpPffplF 14

DA+l++++ +F

g3343.t1 192 DDASLFNYY-VF 202

7******74.77 PP

== domain 9 score: 15.5 bits; conditional E-value: 1.2e-05

round-1-all-seq-for-round-2-input-by-hmmalign 1 eeGDAGlpPffplFGK 16

ee DA+l+++ ++FGK

g3343.t1 208 EEADASLFNY-YVFGK 222

899******7.4***9 PP

== domain 10 score: 22.6 bits; conditional E-value: 6.6e-08

round-1-all-seq-for-round-2-input-by-hmmalign 1 eeGDAGlpPffplFGKeGqq 20

ee DA+l+++ ++FGK+Gq+

g3343.t1 226 EEADASLFNY-YVFGKAGQE 244

899******7.4*******7 PP

== domain 11 score: 0.5 bits; conditional E-value: 0.61

round-1-all-seq-for-round-2-input-by-hmmalign 1 eeGDAG 6

eeGDA+

g3343.t1 244 EEGDAS 249

8****6 PP

>> g53356.t1

# score bias c-Evalue i-Evalue hmmfrom hmm to alifrom ali to envfrom env to acc

--- ------ ----- --------- --------- ------- ------- ------- ------- ------- ------- ----

1 ? -1.1 0.2 2.1 8.3e+03 3 7 .. 65 69 .. 64 69 .. 0.89

2 ! 26.3 0.3 4.5e-09 1.8e-05 3 19 .. 70 87 .. 70 88 .. 0.93

3 ! 10.9 0.0 0.00034 1.4 1 15 [. 93 108 .. 93 111 .. 0.96

4 ! 8.6 0.0 0.0017 6.9 1 15 [. 117 132 .. 117 135 .. 0.95

5 ! 10.9 0.0 0.00034 1.4 1 15 [. 141 156 .. 141 159 .. 0.96

6 ! 10.9 0.0 0.00034 1.4 1 15 [. 165 180 .. 165 183 .. 0.96

7 ! 10.9 0.0 0.00034 1.4 1 15 [. 189 204 .. 189 207 .. 0.96

8 ? -2.0 0.1 3.8 1.5e+04 2 6 .. 214 218 .. 213 219 .. 0.83

Alignments for each domain:

== domain 1 score: -1.1 bits; conditional E-value: 2.1

round-1-all-seq-for-round-2-input-by-hmmalign 3 GDAGl 7

GDAG+

g53356.t1 65 GDAGI 69

9***7 PP

== domain 2 score: 26.3 bits; conditional E-value: 4.5e-09

round-1-all-seq-for-round-2-input-by-hmmalign 3 GDAGlpP.ffplFGKeGq 19

GD+G+pP +++lFGK++

g53356.t1 70 GDDGIPPfWLTLFGKQQA 87

9**************975 PP

== domain 3 score: 10.9 bits; conditional E-value: 0.00034

round-1-all-seq-for-round-2-input-by-hmmalign 1 eeGDAGlpP.ffplFG 15

e+GDAG P + ++FG

g53356.t1 93 EKGDAGMAPmWVTVFG 108

89************** PP

== domain 4 score: 8.6 bits; conditional E-value: 0.0017

round-1-all-seq-for-round-2-input-by-hmmalign 1 eeGDAGlpP.ffplFG 15

e+GDAG P + ++FG

g53356.t1 117 EKGDAGMAPvWGTVFG 132

89*************9 PP

== domain 5 score: 10.9 bits; conditional E-value: 0.00034

round-1-all-seq-for-round-2-input-by-hmmalign 1 eeGDAGlpP.ffplFG 15

e+GDAG P + ++FG

g53356.t1 141 EKGDAGMAPmWVTVFG 156

89************** PP

== domain 6 score: 10.9 bits; conditional E-value: 0.00034

round-1-all-seq-for-round-2-input-by-hmmalign 1 eeGDAGlpP.ffplFG 15

e+GDAG P + ++FG

g53356.t1 165 EKGDAGMAPmWVTVFG 180

89************** PP

== domain 7 score: 10.9 bits; conditional E-value: 0.00034

round-1-all-seq-for-round-2-input-by-hmmalign 1 eeGDAGlpP.ffplFG 15

e+GDAG P + ++FG

g53356.t1 189 EKGDAGMAPvWVTVFG 204

89************** PP

== domain 8 score: -2.0 bits; conditional E-value: 3.8

round-1-all-seq-for-round-2-input-by-hmmalign 2 eGDAG 6

+GDA+

g53356.t1 214 KGDAS 218

8**97 PP

>> g24919.t1

# score bias c-Evalue i-Evalue hmmfrom hmm to alifrom ali to envfrom env to acc

--- ------ ----- --------- --------- ------- ------- ------- ------- ------- ------- ----

1 ? -2.4 0.0 5 2e+04 10 18 .. 72 80 .. 72 81 .. 0.72

2 ! 27.0 0.5 2.9e-09 1.1e-05 1 20 [] 86 105 .. 86 105 .. 0.92

3 ! 34.8 0.3 9.7e-12 3.9e-08 1 20 [] 118 138 .. 118 138 .. 0.97

4 ! 29.8 0.4 3.6e-10 1.5e-06 1 20 [] 157 177 .. 157 177 .. 0.97

5 ? -3.2 1.6 9.2 3.7e+04 15 19 .. 190 194 .. 190 195 .. 0.88

Alignments for each domain:

== domain 1 score: -2.4 bits; conditional E-value: 5

round-1-all-seq-for-round-2-input-by-hmmalign 10 ffplFGKeG 18

++p+ GK G

g24919.t1 72 LSPISGKDG 80

577779987 PP

== domain 2 score: 27.0 bits; conditional E-value: 2.9e-09

round-1-all-seq-for-round-2-input-by-hmmalign 1 eeGDAGlpP.ffplFGKeGqq 20

ee+D++l++ +f +FGKeG+q

g24919.t1 86 EESDGMLVFpLF-IFGKEGSQ 105

8*******7688.******98 PP

== domain 3 score: 34.8 bits; conditional E-value: 9.7e-12

round-1-all-seq-for-round-2-input-by-hmmalign 1 eeGDAGlpP.ffplFGKeGqq 20

ee+D++l+P ff +FGKeG+q

g24919.t1 118 EESDGMLIPpFFVIFGKEGCQ 138

8*******99**********8 PP

== domain 4 score: 29.8 bits; conditional E-value: 3.6e-10

round-1-all-seq-for-round-2-input-by-hmmalign 1 eeGDAGlpP.ffplFGKeGqq 20

ee+D+ l+P ff +FGKeG+q

g24919.t1 157 EESDGILVPpFFLIFGKEGSQ 177

8*******99*********98 PP

== domain 5 score: -3.2 bits; conditional E-value: 9.2

round-1-all-seq-for-round-2-input-by-hmmalign 15 GKeGq 19

GKe+q

g24919.t1 190 GKEQQ 194

9**98 PP

>> g26217.t1

# score bias c-Evalue i-Evalue hmmfrom hmm to alifrom ali to envfrom env to acc

--- ------ ----- --------- --------- ------- ------- ------- ------- ------- ------- ----

1 ! 19.4 0.1 7e-07 0.0028 1 16 [. 56 71 .. 56 74 .. 0.94

2 ! 25.9 0.2 6.1e-09 2.4e-05 1 20 [] 94 114 .. 94 114 .. 0.96

3 ! 29.9 0.7 3.4e-10 1.4e-06 1 20 [] 116 136 .. 116 136 .. 0.97

Alignments for each domain:

== domain 1 score: 19.4 bits; conditional E-value: 7e-07

round-1-all-seq-for-round-2-input-by-hmmalign 1 eeGDAGlpPffplFGK 16

e+GDA+++P lFGK

g26217.t1 56 EHGDAMIFPITRLFGK 71

89*************9 PP

== domain 2 score: 25.9 bits; conditional E-value: 6.1e-09

round-1-all-seq-for-round-2-input-by-hmmalign 1 eeGDAGlpP.ffplFGKeGqq 20

ee DAGl+P +++FGK G++

g26217.t1 94 EEDDAGLFPiPMTVFGKGGMN 114

89****************986 PP

== domain 3 score: 29.9 bits; conditional E-value: 3.4e-10

round-1-all-seq-for-round-2-input-by-hmmalign 1 eeGDAGlpP.ffplFGKeGqq 20

eeGDAGl+P +++FGK G+q

g26217.t1 116 EEGDAGLFPiPMTVFGKGGDQ 136

8******************98 PP

>> g18055.t1

# score bias c-Evalue i-Evalue hmmfrom hmm to alifrom ali to envfrom env to acc

--- ------ ----- --------- --------- ------- ------- ------- ------- ------- ------- ----

1 ? 5.4 0.0 0.018 74 4 17 .. 50 63 .. 49 65 .. 0.78

2 ? 4.0 0.0 0.051 2.1e+02 11 17 .. 71 77 .. 71 79 .. 0.88

3 ? 4.4 0.0 0.038 1.5e+02 11 17 .. 85 91 .. 85 93 .. 0.88

4 ? 4.0 0.0 0.051 2.1e+02 11 17 .. 99 105 .. 99 107 .. 0.88

5 ? -1.4 0.0 2.5 1e+04 11 15 .. 113 117 .. 113 118 .. 0.87

6 ? -1.4 0.0 2.5 1e+04 11 15 .. 127 131 .. 127 132 .. 0.87

7 ? -1.4 0.0 2.5 1e+04 11 15 .. 141 145 .. 141 146 .. 0.87

8 ? -1.4 0.0 2.5 1e+04 11 15 .. 155 159 .. 155 160 .. 0.87

9 ? -1.4 0.0 2.5 1e+04 11 15 .. 169 173 .. 169 174 .. 0.87

10 ? -1.4 0.0 2.5 1e+04 11 15 .. 183 187 .. 183 188 .. 0.87

11 ? -1.4 0.0 2.5 1e+04 11 15 .. 197 201 .. 197 202 .. 0.87

12 ? -1.4 0.0 2.5 1e+04 11 15 .. 211 215 .. 211 216 .. 0.87

13 ? -1.4 0.0 2.5 1e+04 11 15 .. 225 229 .. 225 230 .. 0.87

14 ? 4.0 0.0 0.049 2e+02 11 17 .. 239 245 .. 239 245 .. 0.88

15 ? 3.0 0.0 0.1 4.1e+02 11 17 .. 253 259 .. 253 259 .. 0.85

16 ! 6.7 0.1 0.0068 27 9 17 .. 267 274 .. 262 274 .. 0.76

17 ? 4.4 0.0 0.038 1.5e+02 11 17 .. 282 288 .. 282 290 .. 0.88

18 ? 4.0 0.0 0.048 1.9e+02 11 17 .. 296 302 .. 296 304 .. 0.89

19 ? 4.4 0.0 0.038 1.5e+02 11 17 .. 310 316 .. 310 318 .. 0.88

20 ? 4.4 0.0 0.038 1.5e+02 11 17 .. 324 330 .. 324 332 .. 0.88

21 ? 4.4 0.0 0.038 1.5e+02 11 17 .. 338 344 .. 338 346 .. 0.88

22 ? 4.4 0.0 0.038 1.5e+02 11 17 .. 352 358 .. 352 360 .. 0.88

23 ? 3.0 0.0 0.1 4.1e+02 11 17 .. 366 372 .. 366 372 .. 0.85

24 ! 10.1 0.0 0.00059 2.4 4 17 .. 374 387 .. 373 389 .. 0.86

25 ? -1.7 0.1 3.2 1.3e+04 11 15 .. 395 399 .] 395 399 .] 0.88

Alignments for each domain:

== domain 1 score: 5.4 bits; conditional E-value: 0.018

round-1-all-seq-for-round-2-input-by-hmmalign 4 DAGlp.PffplFGKe 17

D++l+ P p+FGK+

g18055.t1 50 DGSLVrPV-PVFGKQ 63

88888773.6****7 PP

== domain 2 score: 4.0 bits; conditional E-value: 0.051

round-1-all-seq-for-round-2-input-by-hmmalign 11 fplFGKe 17

+p+FGK+

g18055.t1 71 YPVFGKQ 77

69****7 PP

== domain 3 score: 4.4 bits; conditional E-value: 0.038

round-1-all-seq-for-round-2-input-by-hmmalign 11 fplFGKe 17

+p+FGK+

g18055.t1 85 YPIFGKQ 91

69****7 PP

== domain 4 score: 4.0 bits; conditional E-value: 0.051

round-1-all-seq-for-round-2-input-by-hmmalign 11 fplFGKe 17

+p+FGK+

g18055.t1 99 YPVFGKQ 105

69****7 PP

== domain 5 score: -1.4 bits; conditional E-value: 2.5

round-1-all-seq-for-round-2-input-by-hmmalign 11 fplFG 15

+p+FG

g18055.t1 113 YPIFG 117

69*** PP

== domain 6 score: -1.4 bits; conditional E-value: 2.5

round-1-all-seq-for-round-2-input-by-hmmalign 11 fplFG 15

+p+FG

g18055.t1 127 YPIFG 131

69*** PP

== domain 7 score: -1.4 bits; conditional E-value: 2.5

round-1-all-seq-for-round-2-input-by-hmmalign 11 fplFG 15

+p+FG

g18055.t1 141 YPIFG 145

69*** PP

== domain 8 score: -1.4 bits; conditional E-value: 2.5

round-1-all-seq-for-round-2-input-by-hmmalign 11 fplFG 15

+p+FG

g18055.t1 155 YPIFG 159

69*** PP

== domain 9 score: -1.4 bits; conditional E-value: 2.5

round-1-all-seq-for-round-2-input-by-hmmalign 11 fplFG 15

+p+FG

g18055.t1 169 YPIFG 173

69*** PP

== domain 10 score: -1.4 bits; conditional E-value: 2.5

round-1-all-seq-for-round-2-input-by-hmmalign 11 fplFG 15

+p+FG

g18055.t1 183 YPIFG 187

69*** PP

== domain 11 score: -1.4 bits; conditional E-value: 2.5

round-1-all-seq-for-round-2-input-by-hmmalign 11 fplFG 15

+p+FG

g18055.t1 197 YPIFG 201

69*** PP

== domain 12 score: -1.4 bits; conditional E-value: 2.5

round-1-all-seq-for-round-2-input-by-hmmalign 11 fplFG 15

+p+FG

g18055.t1 211 YPIFG 215

69*** PP

== domain 13 score: -1.4 bits; conditional E-value: 2.5

round-1-all-seq-for-round-2-input-by-hmmalign 11 fplFG 15

+p+FG

g18055.t1 225 YPIFG 229

69*** PP

== domain 14 score: 4.0 bits; conditional E-value: 0.049

round-1-all-seq-for-round-2-input-by-hmmalign 11 fplFGKe 17

+p+FGK+

g18055.t1 239 YPIFGKQ 245

69****7 PP

== domain 15 score: 3.0 bits; conditional E-value: 0.1

round-1-all-seq-for-round-2-input-by-hmmalign 11 fplFGKe 17

+p+FGK+

g18055.t1 253 YPIFGKH 259

69****5 PP

== domain 16 score: 6.7 bits; conditional E-value: 0.0068

round-1-all-seq-for-round-2-input-by-hmmalign 9 PffplFGKe 17

P fp+FGK+

g18055.t1 267 P-FPVFGKQ 274

4.6*****7 PP

== domain 17 score: 4.4 bits; conditional E-value: 0.038

round-1-all-seq-for-round-2-input-by-hmmalign 11 fplFGKe 17

+p+FGK+

g18055.t1 282 YPIFGKQ 288

69****7 PP

== domain 18 score: 4.0 bits; conditional E-value: 0.048

round-1-all-seq-for-round-2-input-by-hmmalign 11 fplFGKe 17

+p+FGKe

g18055.t1 296 YPVFGKE 302

69****8 PP

== domain 19 score: 4.4 bits; conditional E-value: 0.038

round-1-all-seq-for-round-2-input-by-hmmalign 11 fplFGKe 17

+p+FGK+

g18055.t1 310 YPIFGKQ 316

69****7 PP

== domain 20 score: 4.4 bits; conditional E-value: 0.038

round-1-all-seq-for-round-2-input-by-hmmalign 11 fplFGKe 17

+p+FGK+

g18055.t1 324 YPIFGKQ 330

69****7 PP

== domain 21 score: 4.4 bits; conditional E-value: 0.038

round-1-all-seq-for-round-2-input-by-hmmalign 11 fplFGKe 17

+p+FGK+

g18055.t1 338 YPIFGKQ 344

69****7 PP

== domain 22 score: 4.4 bits; conditional E-value: 0.038

round-1-all-seq-for-round-2-input-by-hmmalign 11 fplFGKe 17

+p+FGK+

g18055.t1 352 YPIFGKQ 358

69****7 PP

== domain 23 score: 3.0 bits; conditional E-value: 0.1

round-1-all-seq-for-round-2-input-by-hmmalign 11 fplFGKe 17

+p+FGK+

g18055.t1 366 YPIFGKH 372

69****5 PP

== domain 24 score: 10.1 bits; conditional E-value: 0.00059

round-1-all-seq-for-round-2-input-by-hmmalign 4 DAGlp.PffplFGKe 17

D++l+ Pf p+FGK+

g18055.t1 374 DGSLVrPF-PVFGKQ 387

99999786.*****7 PP

== domain 25 score: -1.7 bits; conditional E-value: 3.2

round-1-all-seq-for-round-2-input-by-hmmalign 11 fplFG 15

+p+FG

g18055.t1 395 YPIFG 399

69**9 PP

>> g28070.t1

# score bias c-Evalue i-Evalue hmmfrom hmm to alifrom ali to envfrom env to acc

--- ------ ----- --------- --------- ------- ------- ------- ------- ------- ------- ----

1 ! 26.5 0.6 4e-09 1.6e-05 2 16 .. 19 33 .. 18 35 .. 0.92

2 ! 28.7 0.6 8e-10 3.2e-06 2 18 .. 35 51 .. 34 51 .. 0.93

3 ! 33.5 2.5 2.4e-11 9.7e-08 1 19 [. 50 68 .. 50 69 .. 0.94

4 ? 4.0 2.1 0.048 1.9e+02 1 16 [. 66 78 .. 66 79 .. 0.75

5 ? 0.2 2.3 0.77 3.1e+03 3 16 .. 92 102 .. 91 105 .. 0.70

6 ? -0.1 1.0 0.96 3.8e+03 13 16 .. 123 126 .. 116 127 .. 0.67

7 ? 1.1 0.0 0.4 1.6e+03 3 7 .. 140 144 .. 138 144 .. 0.90

8 ? -1.6 0.0 2.9 1.1e+04 1 7 [. 155 161 .. 155 161 .. 0.85

Alignments for each domain:

== domain 1 score: 26.5 bits; conditional E-value: 4e-09

round-1-all-seq-for-round-2-input-by-hmmalign 2 eGDAGlpPffplFGK 16

+GDAG+pP++plFG+

g28070.t1 19 NGDAGYPPLSPLFGQ 33

7*************6 PP

== domain 2 score: 28.7 bits; conditional E-value: 8e-10

round-1-all-seq-for-round-2-input-by-hmmalign 2 eGDAGlpPffplFGKeG 18

+GDAG+pP++plFG+e+

g28070.t1 35 NGDAGYPPLSPLFGQEK 51

7*************986 PP

== domain 3 score: 33.5 bits; conditional E-value: 2.4e-11

round-1-all-seq-for-round-2-input-by-hmmalign 1 eeGDAGlpPffplFGKeGq 19

e+GDAG+pP++plFGKe+

g28070.t1 50 EKGDAGYPPLSPLFGKEKG 68

89***************86 PP

== domain 4 score: 4.0 bits; conditional E-value: 0.048

round-1-all-seq-for-round-2-input-by-hmmalign 1 eeGDAGlpPffplFGK 16

e+GDAG+ + FGK

g28070.t1 66 EKGDAGYNGL---FGK 78

89****9554...665 PP

== domain 5 score: 0.2 bits; conditional E-value: 0.77

round-1-all-seq-for-round-2-input-by-hmmalign 3 GDAGlpPffplFGK 16

GDAG+ + FGK

g28070.t1 92 GDAGYNGL---FGK 102

78888444...676 PP

== domain 6 score: -0.1 bits; conditional E-value: 0.96

round-1-all-seq-for-round-2-input-by-hmmalign 13 lFGK 16

lFGK

g28070.t1 123 LFGK 126

5888 PP

== domain 7 score: 1.1 bits; conditional E-value: 0.4

round-1-all-seq-for-round-2-input-by-hmmalign 3 GDAGl 7

GDAG+

g28070.t1 140 GDAGY 144

9***9 PP

== domain 8 score: -1.6 bits; conditional E-value: 2.9

round-1-all-seq-for-round-2-input-by-hmmalign 1 eeGDAGl 7

e GD G+

g28070.t1 155 EVGDTGY 161

679***9 PP

>> g33422.t1

# score bias c-Evalue i-Evalue hmmfrom hmm to alifrom ali to envfrom env to acc

--- ------ ----- --------- --------- ------- ------- ------- ------- ------- ------- ----

1 ? -1.9 0.1 3.6 1.4e+04 6 10 .. 27 32 .. 27 32 .. 0.81

2 ! 28.2 0.0 1.2e-09 4.8e-06 1 20 [] 58 78 .. 58 78 .. 0.94

3 ! 12.4 0.2 0.00011 0.43 3 15 .. 85 98 .. 84 98 .. 0.93

4 ! 24.1 0.0 2.4e-08 9.5e-05 1 17 [. 100 117 .. 100 120 .. 0.94

Alignments for each domain:

== domain 1 score: -1.9 bits; conditional E-value: 3.6

round-1-all-seq-for-round-2-input-by-hmmalign 6 GlpP.f 10

GlpP +

g33422.t1 27 GLPPvL 32

89**66 PP

== domain 2 score: 28.2 bits; conditional E-value: 1.2e-09

round-1-all-seq-for-round-2-input-by-hmmalign 1 eeGDAGlpP.ffplFGKeGqq 20

e+GDAG+pP +++l GK++++

g33422.t1 58 ERGDAGIPPfWLTLVGKQRTD 78

89****************875 PP

== domain 3 score: 12.4 bits; conditional E-value: 0.00011

round-1-all-seq-for-round-2-input-by-hmmalign 3 GDAGlpP.ffplFG 15

GDAGlpP + +FG

g33422.t1 85 GDAGLPPmWVEVFG 98

9********99998 PP

== domain 4 score: 24.1 bits; conditional E-value: 2.4e-08

round-1-all-seq-for-round-2-input-by-hmmalign 1 eeGDAGlpP.ffplFGKe 17

e+GDAG+pP +++l GK+

g33422.t1 100 ERGDAGIPPfWLTLIGKH 117

89***************6 PP

>> g15288.t1

# score bias c-Evalue i-Evalue hmmfrom hmm to alifrom ali to envfrom env to acc

--- ------ ----- --------- --------- ------- ------- ------- ------- ------- ------- ----

1 ? 3.0 0.0 0.1 4.2e+02 12 17 .. 35 40 .. 33 41 .. 0.85

2 ? 3.0 0.0 0.1 4.2e+02 12 17 .. 48 53 .. 46 55 .. 0.86

3 ? 1.9 0.0 0.23 9.2e+02 2 17 .. 60 73 .. 60 73 .. 0.80

4 ? 3.0 0.0 0.1 4e+02 12 17 .. 80 85 .. 78 86 .. 0.85

5 ? 2.6 0.0 0.14 5.5e+02 12 17 .. 93 98 .. 92 99 .. 0.87

6 ? 3.0 0.0 0.1 4e+02 12 17 .. 106 111 .. 104 112 .. 0.85

7 ? 3.8 0.0 0.058 2.3e+02 12 17 .. 119 124 .. 118 127 .. 0.88

8 ? 3.0 0.0 0.1 4e+02 12 17 .. 133 138 .. 131 139 .. 0.85

9 ? 2.4 0.1 0.15 6.2e+02 12 17 .. 146 151 .. 145 151 .. 0.86

10 ? 4.0 0.0 0.05 2e+02 12 17 .. 159 164 .. 155 167 .. 0.87

11 ? 4.1 0.0 0.045 1.8e+02 12 17 .. 173 178 .. 171 181 .. 0.86

12 ? 4.1 0.0 0.045 1.8e+02 12 17 .. 187 192 .. 185 195 .. 0.86

13 ? 3.2 0.0 0.085 3.4e+02 12 17 .. 201 206 .. 199 209 .. 0.84

14 ? 1.2 0.1 0.38 1.5e+03 3 17 .. 213 225 .. 212 225 .. 0.78

15 ? 3.8 0.0 0.058 2.3e+02 12 17 .. 233 238 .. 232 241 .. 0.88

16 ? -1.5 0.2 2.7 1.1e+04 3 10 .. 245 254 .. 244 255 .] 0.78

Alignments for each domain:

== domain 1 score: 3.0 bits; conditional E-value: 0.1

round-1-all-seq-for-round-2-input-by-hmmalign 12 plFGKe 17

p+FGK+

g15288.t1 35 PVFGKA 40

8****6 PP

== domain 2 score: 3.0 bits; conditional E-value: 0.1

round-1-all-seq-for-round-2-input-by-hmmalign 12 plFGKe 17

p+FGK+

g15288.t1 48 PVFGKA 53

8****6 PP

== domain 3 score: 1.9 bits; conditional E-value: 0.23

round-1-all-seq-for-round-2-input-by-hmmalign 2 eGDAGlp.PffplFGKe 17

+GD G++ P FGK+

g15288.t1 60 RGDSGYVvPC---FGKA 73

6899999996...7985 PP

== domain 4 score: 3.0 bits; conditional E-value: 0.1

round-1-all-seq-for-round-2-input-by-hmmalign 12 plFGKe 17

p+FGK+

g15288.t1 80 PVFGKA 85

8****6 PP

== domain 5 score: 2.6 bits; conditional E-value: 0.14

round-1-all-seq-for-round-2-input-by-hmmalign 12 plFGKe 17

p+FGK+

g15288.t1 93 PVFGKA 98

8****6 PP

== domain 6 score: 3.0 bits; conditional E-value: 0.1

round-1-all-seq-for-round-2-input-by-hmmalign 12 plFGKe 17

p+FGK+

g15288.t1 106 PVFGKA 111

8****6 PP

== domain 7 score: 3.8 bits; conditional E-value: 0.058

round-1-all-seq-for-round-2-input-by-hmmalign 12 plFGKe 17

p+FGKe

g15288.t1 119 PVFGKE 124

8****8 PP

== domain 8 score: 3.0 bits; conditional E-value: 0.1

round-1-all-seq-for-round-2-input-by-hmmalign 12 plFGKe 17

p+FGK+

g15288.t1 133 PVFGKA 138

8****6 PP

== domain 9 score: 2.4 bits; conditional E-value: 0.15

round-1-all-seq-for-round-2-input-by-hmmalign 12 plFGKe 17

p+FGK+

g15288.t1 146 PVFGKA 151

8****6 PP

== domain 10 score: 4.0 bits; conditional E-value: 0.05

round-1-all-seq-for-round-2-input-by-hmmalign 12 plFGKe 17

p+FGKe

g15288.t1 159 PVFGKE 164

8****8 PP

== domain 11 score: 4.1 bits; conditional E-value: 0.045

round-1-all-seq-for-round-2-input-by-hmmalign 12 plFGKe 17

p+FGKe

g15288.t1 173 PVFGKE 178

8****8 PP

== domain 12 score: 4.1 bits; conditional E-value: 0.045

round-1-all-seq-for-round-2-input-by-hmmalign 12 plFGKe 17

p+FGKe

g15288.t1 187 PVFGKE 192

8****8 PP

== domain 13 score: 3.2 bits; conditional E-value: 0.085

round-1-all-seq-for-round-2-input-by-hmmalign 12 plFGKe 17

p+FGK+

g15288.t1 201 PVFGKA 206

8****7 PP

== domain 14 score: 1.2 bits; conditional E-value: 0.38

round-1-all-seq-for-round-2-input-by-hmmalign 3 GDAGlp.PffplFGKe 17

GD G++ P FGK+

g15288.t1 213 GDSGYVvPC---FGKA 225

888999886...7985 PP

== domain 15 score: 3.8 bits; conditional E-value: 0.058

round-1-all-seq-for-round-2-input-by-hmmalign 12 plFGKe 17

p+FGKe

g15288.t1 233 PVFGKE 238

8****8 PP

== domain 16 score: -1.5 bits; conditional E-value: 2.7

round-1-all-seq-for-round-2-input-by-hmmalign 3 GDAGlp.P.f 10

GD G++ P f

g15288.t1 245 GDSGYVvPcF 254

8999998855 PP

>> g46938.t1

# score bias c-Evalue i-Evalue hmmfrom hmm to alifrom ali to envfrom env to acc

--- ------ ----- --------- --------- ------- ------- ------- ------- ------- ------- ----

1 ? 5.4 0.1 0.018 72 4 15 .. 23 33 .. 22 33 .. 0.88

2 ! 17.2 0.3 3.3e-06 0.013 1 16 [. 41 57 .. 41 57 .. 0.90

3 ! 21.8 0.2 1.2e-07 0.00049 2 17 .. 64 79 .. 64 80 .. 0.91

4 ! 14.0 0.4 3.4e-05 0.14 4 17 .. 80 94 .. 79 95 .. 0.91

5 ? 3.9 6.9 0.055 2.2e+02 2 17 .. 100 115 .. 99 115 .. 0.82

6 ! 7.5 1.2 0.004 16 9 17 .. 128 137 .. 123 137 .. 0.80

7 ? -1.6 0.1 2.8 1.1e+04 2 6 .. 144 148 .. 143 148 .. 0.83

Alignments for each domain:

== domain 1 score: 5.4 bits; conditional E-value: 0.018

round-1-all-seq-for-round-2-input-by-hmmalign 4 DAGlpP.ffplFG 15

DAGl+P + FG

g46938.t1 23 DAGLIPvPM--FG 33

******887..88 PP

== domain 2 score: 17.2 bits; conditional E-value: 3.3e-06

round-1-all-seq-for-round-2-input-by-hmmalign 1 eeGDAGlp.PffplFGK 16

+eGDAG++ P + +FGK

g46938.t1 41 PEGDAGIIdPILLIFGK 57

69******77889***9 PP

== domain 3 score: 21.8 bits; conditional E-value: 1.2e-07

round-1-all-seq-for-round-2-input-by-hmmalign 2 eGDAGlpPffplFGKe 17

+GDAG +P f++FGKe

g46938.t1 64 NGDAGFVPNFSVFGKE 79

59******888****8 PP

== domain 4 score: 14.0 bits; conditional E-value: 3.4e-05

round-1-all-seq-for-round-2-input-by-hmmalign 4 DAGlp.PffplFGKe 17

DA l+ P fp+FGKe

g46938.t1 80 DAALFiPSFPIFGKE 94

899999*99*****8 PP

== domain 5 score: 3.9 bits; conditional E-value: 0.055

round-1-all-seq-for-round-2-input-by-hmmalign 2 eGDAGlpP.ffplFGKe 17

+GDAG + ffp FGK+

g46938.t1 100 KGDAG-FDiFFPFFGKA 115

89999.7779999***6 PP

== domain 6 score: 7.5 bits; conditional E-value: 0.004

round-1-all-seq-for-round-2-input-by-hmmalign 9 P.ffplFGKe 17

ffp FGK+

g46938.t1 128 DiFFPFFGKQ 137

558889***7 PP

== domain 7 score: -1.6 bits; conditional E-value: 2.8

round-1-all-seq-for-round-2-input-by-hmmalign 2 eGDAG 6

+GD+G

g46938.t1 144 NGDDG 148

79*98 PP

>> g24918.t1

# score bias c-Evalue i-Evalue hmmfrom hmm to alifrom ali to envfrom env to acc

--- ------ ----- --------- --------- ------- ------- ------- ------- ------- ------- ----

1 ! 26.5 0.1 4.1e-09 1.6e-05 2 16 .. 49 63 .. 49 64 .. 0.94

2 ! 25.3 0.9 9.4e-09 3.8e-05 4 19 .. 67 82 .. 66 83 .. 0.91

3 ? 2.1 2.1 0.19 7.8e+02 1 17 [. 80 97 .. 80 98 .. 0.69

4 ? 1.1 2.4 0.42 1.7e+03 1 17 [. 100 117 .. 100 118 .. 0.64

5 ? 1.1 2.4 0.42 1.7e+03 1 17 [. 120 137 .. 120 138 .. 0.64

6 ? 1.1 2.4 0.42 1.7e+03 1 17 [. 140 157 .. 140 158 .. 0.64

7 ? 1.1 2.4 0.42 1.7e+03 1 17 [. 160 177 .. 160 178 .. 0.64

8 ? 1.4 2.2 0.33 1.3e+03 1 17 [. 180 197 .. 180 199 .. 0.65

9 ? 2.9 2.6 0.11 4.5e+02 7 16 .. 211 218 .. 202 219 .. 0.72

Alignments for each domain:

== domain 1 score: 26.5 bits; conditional E-value: 4.1e-09

round-1-all-seq-for-round-2-input-by-hmmalign 2 eGDAGlpPffplFGK 16

+GDAG+pP++plFG+

g24918.t1 49 KGDAGYPPLSPLFGQ 63

7*************5 PP

== domain 2 score: 25.3 bits; conditional E-value: 9.4e-09

round-1-all-seq-for-round-2-input-by-hmmalign 4 DAGlpPffplFGKeGq 19

DAG+pP++plFG+e+

g24918.t1 67 DAGYPPLSPLFGQEKG 82

*************986 PP

== domain 3 score: 2.1 bits; conditional E-value: 0.19

round-1-all-seq-for-round-2-input-by-hmmalign 1 eeGDAGlp.P.ffplFGKe 17

e+GDAG + + +FGK

g24918.t1 80 EKGDAGVYgFiPH-VFGKG 97

7899985556666.89984 PP

== domain 4 score: 1.1 bits; conditional E-value: 0.42

round-1-all-seq-for-round-2-input-by-hmmalign 1 eeGDAGlp.P.ffplFGKe 17

e+GDAG + + +FGK

g24918.t1 100 ENGDAGVYgFiPH-VFGKG 117

6788874446556.89984 PP

== domain 5 score: 1.1 bits; conditional E-value: 0.42

round-1-all-seq-for-round-2-input-by-hmmalign 1 eeGDAGlp.P.ffplFGKe 17

e+GDAG + + +FGK

g24918.t1 120 ENGDAGVYgFiPH-VFGKG 137

6788874446556.89984 PP

== domain 6 score: 1.1 bits; conditional E-value: 0.42

round-1-all-seq-for-round-2-input-by-hmmalign 1 eeGDAGlp.P.ffplFGKe 17

e+GDAG + + +FGK

g24918.t1 140 ENGDAGVYgFiPH-VFGKG 157

6788874446556.89984 PP

== domain 7 score: 1.1 bits; conditional E-value: 0.42

round-1-all-seq-for-round-2-input-by-hmmalign 1 eeGDAGlp.P.ffplFGKe 17

e+GDAG + + +FGK

g24918.t1 160 ENGDAGVYgFiPH-VFGKG 177

6788874446556.89984 PP

== domain 8 score: 1.4 bits; conditional E-value: 0.33

round-1-all-seq-for-round-2-input-by-hmmalign 1 eeGDAGlp.P.ffplFGKe 17

e+GDAG + + +FGK

g24918.t1 180 ENGDAGVYgFiPH-VFGKG 197

6778874446556.89995 PP

== domain 9 score: 2.9 bits; conditional E-value: 0.11

round-1-all-seq-for-round-2-input-by-hmmalign 7 lpPffplFGK 16

++P ++FGK

g24918.t1 211 YFP--MIFGK 218

555..79**9 PP

>> g2811.t1

# score bias c-Evalue i-Evalue hmmfrom hmm to alifrom ali to envfrom env to acc

--- ------ ----- --------- --------- ------- ------- ------- ------- ------- ------- ----

1 ! 9.5 0.0 0.00094 3.8 1 17 [. 45 60 .. 45 61 .. 0.90

2 ! 15.4 0.1 1.2e-05 0.05 2 16 .. 66 81 .. 65 81 .. 0.90

3 ? 4.9 6.8 0.026 1e+02 2 17 .. 96 111 .. 95 111 .. 0.83

4 ? 4.2 1.9 0.042 1.7e+02 4 15 .. 120 131 .. 117 131 .. 0.88

Alignments for each domain:

== domain 1 score: 9.5 bits; conditional E-value: 0.00094

round-1-all-seq-for-round-2-input-by-hmmalign 1 eeGDAGlpP.ffplFGKe 17

++GDA l+P + FGK+

g2811.t1 45 SKGDATLIPvPM--FGKA 60

69*******998..***6 PP

== domain 2 score: 15.4 bits; conditional E-value: 1.2e-05

round-1-all-seq-for-round-2-input-by-hmmalign 2 eGDAGlp.PffplFGK 16

+GDAG++ P +p+FGK

g2811.t1 66 KGDAGIIdPTLPIFGK 81

7******8866****9 PP

== domain 3 score: 4.9 bits; conditional E-value: 0.026

round-1-all-seq-for-round-2-input-by-hmmalign 2 eGDAGlpP.ffplFGKe 17

+GDAG + ffp FGK+

g2811.t1 96 KGDAG-FDiFFPFFGKQ 111

89999.7779999***7 PP

== domain 4 score: 4.2 bits; conditional E-value: 0.042

round-1-all-seq-for-round-2-input-by-hmmalign 4 DAGlpPffplFG 15

DAG +ffp FG

g2811.t1 120 DAGFDNFFPFFG 131

899889*****9 PP

>> g23576.t1

# score bias c-Evalue i-Evalue hmmfrom hmm to alifrom ali to envfrom env to acc

--- ------ ----- --------- --------- ------- ------- ------- ------- ------- ------- ----

1 ? -0.5 0.0 1.3 5.1e+03 14 19 .. 2 7 .. 1 8 [. 0.81

2 ? 4.9 0.0 0.026 1.1e+02 11 19 .. 16 24 .. 16 25 .. 0.82

3 ? 4.9 0.0 0.026 1.1e+02 11 19 .. 33 41 .. 33 42 .. 0.82

4 ? -2.5 0.3 5.7 2.3e+04 12 15 .. 44 47 .. 44 50 .. 0.73

5 ? -0.5 0.0 1.3 5.1e+03 14 19 .. 53 58 .. 52 59 .. 0.81

6 ? 2.7 0.0 0.13 5.2e+02 11 16 .. 67 72 .. 67 76 .. 0.85

7 ? -2.5 0.3 5.7 2.3e+04 12 15 .. 78 81 .. 78 84 .. 0.73

8 ? -0.5 0.0 1.3 5.1e+03 14 19 .. 87 92 .. 86 93 .. 0.81

9 ? 4.9 0.0 0.026 1.1e+02 11 19 .. 101 109 .. 101 110 .. 0.82

10 ? -2.9 0.0 7.4 3e+04 12 15 .. 112 115 .. 112 117 .. 0.82

11 ? 4.9 0.0 0.026 1.1e+02 11 19 .. 135 143 .. 135 144 .. 0.82

12 ? -2.9 0.0 7.4 3e+04 12 15 .. 146 149 .. 146 151 .. 0.82

13 ? 4.9 0.0 0.026 1.1e+02 11 19 .. 169 177 .. 169 178 .. 0.82

14 ? -2.0 0.1 3.8 1.5e+04 12 19 .. 180 185 .. 180 186 .. 0.72

15 ? -0.8 0.1 1.6 6.4e+03 14 19 .. 189 194 .. 189 195 .. 0.80

16 ? -2.5 0.3 5.7 2.3e+04 12 15 .. 205 208 .. 205 211 .. 0.73

17 ? -0.5 0.0 1.3 5.1e+03 14 19 .. 214 219 .. 213 220 .. 0.81

18 ? 2.9 0.0 0.11 4.3e+02 12 18 .. 226 232 .. 226 234 .. 0.84

19 ? 2.8 0.1 0.12 4.9e+02 12 18 .. 241 247 .. 241 248 .. 0.74

Alignments for each domain:

== domain 1 score: -0.5 bits; conditional E-value: 1.3

round-1-all-seq-for-round-2-input-by-hmmalign 14 FGKeGq 19

FGK+ q

g23576.t1 2 FGKAHQ 7

***966 PP

== domain 2 score: 4.9 bits; conditional E-value: 0.026

round-1-all-seq-for-round-2-input-by-hmmalign 11 fplFGKeGq 19

++lFGK+ q

g23576.t1 16 SSLFGKAHQ 24

79****966 PP

== domain 3 score: 4.9 bits; conditional E-value: 0.026

round-1-all-seq-for-round-2-input-by-hmmalign 11 fplFGKeGq 19

++lFGK+ q

g23576.t1 33 SSLFGKAHQ 41

79****966 PP

== domain 4 score: -2.5 bits; conditional E-value: 5.7

round-1-all-seq-for-round-2-input-by-hmmalign 12 plFG 15

+lFG

g23576.t1 44 SLFG 47

59*9 PP

== domain 5 score: -0.5 bits; conditional E-value: 1.3

round-1-all-seq-for-round-2-input-by-hmmalign 14 FGKeGq 19

FGK+ q

g23576.t1 53 FGKAHQ 58

***966 PP

== domain 6 score: 2.7 bits; conditional E-value: 0.13

round-1-all-seq-for-round-2-input-by-hmmalign 11 fplFGK 16

++lFGK

g23576.t1 67 SSLFGK 72

79***9 PP

== domain 7 score: -2.5 bits; conditional E-value: 5.7

round-1-all-seq-for-round-2-input-by-hmmalign 12 plFG 15

+lFG

g23576.t1 78 SLFG 81

59*9 PP

== domain 8 score: -0.5 bits; conditional E-value: 1.3

round-1-all-seq-for-round-2-input-by-hmmalign 14 FGKeGq 19

FGK+ q

g23576.t1 87 FGKAHQ 92

***966 PP

== domain 9 score: 4.9 bits; conditional E-value: 0.026

round-1-all-seq-for-round-2-input-by-hmmalign 11 fplFGKeGq 19

++lFGK+ q

g23576.t1 101 SSLFGKAHQ 109

79****966 PP

== domain 10 score: -2.9 bits; conditional E-value: 7.4

round-1-all-seq-for-round-2-input-by-hmmalign 12 plFG 15

+lFG

g23576.t1 112 SLFG 115

59*9 PP

== domain 11 score: 4.9 bits; conditional E-value: 0.026

round-1-all-seq-for-round-2-input-by-hmmalign 11 fplFGKeGq 19

++lFGK+ q

g23576.t1 135 SSLFGKAHQ 143

79****966 PP

== domain 12 score: -2.9 bits; conditional E-value: 7.4

round-1-all-seq-for-round-2-input-by-hmmalign 12 plFG 15

+lFG

g23576.t1 146 SLFG 149

59*9 PP

== domain 13 score: 4.9 bits; conditional E-value: 0.026

round-1-all-seq-for-round-2-input-by-hmmalign 11 fplFGKeGq 19

++lFGK+ q

g23576.t1 169 SSLFGKAHQ 177

79****966 PP

== domain 14 score: -2.0 bits; conditional E-value: 3.8

round-1-all-seq-for-round-2-input-by-hmmalign 12 plFGKeGq 19

+lFG Gq

g23576.t1 180 SLFG--GQ 185

59*9..55 PP

== domain 15 score: -0.8 bits; conditional E-value: 1.6

round-1-all-seq-for-round-2-input-by-hmmalign 14 FGKeGq 19

FGK+ q

g23576.t1 189 FGKAHQ 194

***966 PP

== domain 16 score: -2.5 bits; conditional E-value: 5.7

round-1-all-seq-for-round-2-input-by-hmmalign 12 plFG 15

+lFG

g23576.t1 205 SLFG 208

59*9 PP

== domain 17 score: -0.5 bits; conditional E-value: 1.3

round-1-all-seq-for-round-2-input-by-hmmalign 14 FGKeGq 19

FGK+ q

g23576.t1 214 FGKAHQ 219

***966 PP

== domain 18 score: 2.9 bits; conditional E-value: 0.11

round-1-all-seq-for-round-2-input-by-hmmalign 12 plFGKeG 18

+lFGK++

g23576.t1 226 TLFGKAR 232

69***85 PP

== domain 19 score: 2.8 bits; conditional E-value: 0.12

round-1-all-seq-for-round-2-input-by-hmmalign 12 plFGKeG 18

+lFGK +

g23576.t1 241 SLFGKGQ 247

69***64 PP

>> g56299.t1

# score bias c-Evalue i-Evalue hmmfrom hmm to alifrom ali to envfrom env to acc

--- ------ ----- --------- --------- ------- ------- ------- ------- ------- ------- ----

1 ? 1.5 0.1 0.3 1.2e+03 13 17 .. 69 73 .. 69 74 .. 0.89

2 ? 1.5 0.1 0.3 1.2e+03 13 17 .. 88 92 .. 88 93 .. 0.89

3 ? 1.6 0.1 0.28 1.1e+03 13 17 .. 101 105 .. 101 106 .. 0.91

4 ? 1.5 0.1 0.3 1.2e+03 13 17 .. 114 118 .. 114 119 .. 0.89

5 ? 1.5 0.1 0.3 1.2e+03 13 17 .. 133 137 .. 133 138 .. 0.89

6 ? 1.6 0.1 0.28 1.1e+03 13 17 .. 146 150 .. 146 151 .. 0.91

7 ? 1.5 0.1 0.3 1.2e+03 13 17 .. 159 163 .. 159 164 .. 0.89

8 ? 1.5 0.1 0.3 1.2e+03 13 17 .. 178 182 .. 178 183 .. 0.89

9 ? 7.6 0.0 0.0036 15 4 17 .. 197 208 .. 195 208 .. 0.86

Alignments for each domain:

== domain 1 score: 1.5 bits; conditional E-value: 0.3

round-1-all-seq-for-round-2-input-by-hmmalign 13 lFGKe 17

+FGK+

g56299.t1 69 IFGKQ 73

8***7 PP

== domain 2 score: 1.5 bits; conditional E-value: 0.3

round-1-all-seq-for-round-2-input-by-hmmalign 13 lFGKe 17

+FGK+

g56299.t1 88 IFGKQ 92

8***7 PP

== domain 3 score: 1.6 bits; conditional E-value: 0.28

round-1-all-seq-for-round-2-input-by-hmmalign 13 lFGKe 17

+FGKe

g56299.t1 101 IFGKE 105

8***8 PP

== domain 4 score: 1.5 bits; conditional E-value: 0.3

round-1-all-seq-for-round-2-input-by-hmmalign 13 lFGKe 17

+FGK+

g56299.t1 114 IFGKQ 118

8***7 PP

== domain 5 score: 1.5 bits; conditional E-value: 0.3

round-1-all-seq-for-round-2-input-by-hmmalign 13 lFGKe 17

+FGK+

g56299.t1 133 IFGKQ 137

8***7 PP

== domain 6 score: 1.6 bits; conditional E-value: 0.28

round-1-all-seq-for-round-2-input-by-hmmalign 13 lFGKe 17

+FGKe

g56299.t1 146 IFGKE 150

8***8 PP

== domain 7 score: 1.5 bits; conditional E-value: 0.3

round-1-all-seq-for-round-2-input-by-hmmalign 13 lFGKe 17

+FGK+

g56299.t1 159 IFGKQ 163

8***7 PP

== domain 8 score: 1.5 bits; conditional E-value: 0.3

round-1-all-seq-for-round-2-input-by-hmmalign 13 lFGKe 17

+FGK+

g56299.t1 178 IFGKQ 182

8***7 PP

== domain 9 score: 7.6 bits; conditional E-value: 0.0036

round-1-all-seq-for-round-2-input-by-hmmalign 4 DAGlpP.ffplFGKe 17

D+G+++ + FGK+

g56299.t1 197 DGGCIFrL---FGKQ 208

99999977...***7 PP

Round 3 HMM search output:

Query: round-2-all-seq-for-round-3-input-by-hmmalign [M=23]

Scores for complete sequences (score includes all domains):

--- full sequence --- --- best 1 domain --- -#dom-

E-value score bias E-value score bias exp N Sequence Description

------- ------ ----- ------- ------ ----- ---- -- -------- -----------

3.8e-65 216.9 127.6 5.6e-08 34.2 1.1 23.4 26 g34966.t1

2.6e-49 166.3 41.2 1.4e-06 29.8 0.1 10.7 11 g3343.t1

6.9e-45 152.2 28.5 1.8e-08 35.8 0.3 7.1 7 g7437.t1

8.3e-35 120.0 49.3 5.2e-05 24.8 0.1 9.6 9 g24918.t1

1e-29 103.7 19.8 3.9e-11 44.3 0.6 4.2 4 g24919.t1

4.8e-22 79.2 19.8 1.5e-09 39.2 3.7 9.1 8 g28070.t1

9.6e-22 78.2 9.9 2.7e-07 32.1 1.0 4.0 3 g26217.t1

2.8e-21 76.7 18.4 5.2e-05 24.8 0.1 7.9 8 g53356.t1

6.2e-20 72.4 1.6 5.7e-07 31.0 0.0 3.5 4 g33422.t1

7.9e-16 59.3 43.4 0.00025 22.6 0.4 6.7 7 g46938.t1

6.5e-13 50.0 35.2 73 5.1 0.0 15.3 16 g15288.t1

5.3e-11 43.9 97.7 0.32 12.7 0.0 22.7 25 g18055.t1

1.6e-10 42.4 25.4 0.0015 20.1 0.3 4.2 4 g2811.t1

------ inclusion threshold ------

2.3 9.9 30.6 8.8 8.1 0.0 8.9 9 g56299.t1

Domain annotation for each sequence (and alignments):

>> g34966.t1

# score bias c-Evalue i-Evalue hmmfrom hmm to alifrom ali to envfrom env to acc

--- ------ ----- --------- --------- ------- ------- ------- ------- ------- ------- ----

1 ! 23.1 0.7 4e-08 0.00017 5 23 .] 35 54 .. 34 54 .. 0.88

2 ! 10.5 0.7 0.00034 1.5 9 19 .. 55 65 .. 55 68 .. 0.91

3 ! 13.9 0.1 3e-05 0.13 2 18 .. 64 80 .. 64 81 .. 0.91

4 ! 19.1 0.2 7.4e-07 0.0032 4 19 .. 98 113 .. 96 114 .. 0.86

5 ! 28.8 1.3 6.6e-10 2.8e-06 1 22 [. 111 132 .. 111 133 .. 0.95

6 ? 4.0 0.0 0.038 1.6e+02 9 19 .. 135 145 .. 134 146 .. 0.90

7 ! 28.7 0.6 6.9e-10 3e-06 1 19 [. 143 161 .. 143 162 .. 0.96

8 ! 29.2 0.8 5.1e-10 2.2e-06 2 21 .. 160 179 .. 160 179 .. 0.95

9 ! 33.2 1.0 2.7e-11 1.2e-07 1 20 [. 175 194 .. 175 194 .. 0.97

10 ! 30.4 0.7 2.1e-10 9.2e-07 1 19 [. 191 209 .. 191 210 .. 0.96

11 ! 30.0 0.7 2.8e-10 1.2e-06 1 22 [. 207 228 .. 207 228 .. 0.95

12 ! 34.2 1.1 1.3e-11 5.6e-08 1 21 [. 223 243 .. 223 243 .. 0.96

13 ! 29.7 0.6 3.5e-10 1.5e-06 1 19 [. 239 257 .. 239 258 .. 0.96

14 ! 10.1 0.7 0.00047 2 2 16 .. 256 270 .. 256 272 .. 0.85

15 ! 21.0 0.1 1.9e-07 0.0008 3 18 .. 274 291 .. 274 291 .. 0.98

16 ? -0.0 0.1 0.71 3.1e+03 4 10 .. 335 341 .. 335 341 .. 0.89

17 ? -0.0 0.1 0.71 3.1e+03 4 10 .. 352 358 .. 352 358 .. 0.89

18 ? -0.0 0.1 0.71 3.1e+03 4 10 .. 369 375 .. 369 375 .. 0.89

19 ? -0.0 0.1 0.71 3.1e+03 4 10 .. 386 392 .. 386 392 .. 0.89

20 ? -0.0 0.1 0.71 3.1e+03 4 10 .. 403 409 .. 403 409 .. 0.89

21 ? -0.0 0.1 0.71 3.1e+03 4 10 .. 420 426 .. 420 426 .. 0.89

22 ? -0.0 0.1 0.71 3.1e+03 4 10 .. 437 443 .. 437 443 .. 0.89

23 ? -1.7 0.4 2.4 1e+04 6 10 .. 456 460 .. 454 460 .. 0.90

24 ? 0.5 0.2 0.47 2e+03 4 10 .. 471 477 .. 470 478 .. 0.88

25 ? 0.5 0.2 0.47 2e+03 4 10 .. 488 494 .. 487 495 .. 0.88

26 ? -0.0 0.1 0.71 3.1e+03 4 10 .. 505 511 .. 505 511 .. 0.89

Alignments for each domain:

== domain 1 score: 23.1 bits; conditional E-value: 4e-08

round-2-all-seq-for-round-3-input-by-hmmalign 5 eGDAGlpPffpiFGKeGq.d 23

+ D+G+pP++p+FGK ++ +

g34966.t1 35 KRDGGYPPLSPLFGKKKWdV 54

67*************98855 PP

== domain 2 score: 10.5 bits; conditional E-value: 0.00034

round-2-all-seq-for-round-3-input-by-hmmalign 9 GlpPffpiFGK 19

G+pP++p+FG+

g34966.t1 55 GYPPLSPLFGQ 65

8*********7 PP

== domain 3 score: 13.9 bits; conditional E-value: 3e-05

round-2-all-seq-for-round-3-input-by-hmmalign 2 gqEeGDAGlpPffpiFG 18

gq + DA++pP++++FG

g34966.t1 64 GQKKNDASYPPLSSLFG 80

67888************ PP

== domain 4 score: 19.1 bits; conditional E-value: 7.4e-07

round-2-all-seq-for-round-3-input-by-hmmalign 4 EeGDAGlpPffpiFGK 19

E+ DAG+pP++p+FG+

g34966.t1 98 EKKDAGYPPLSPLFGQ 113

666************6 PP

== domain 5 score: 28.8 bits; conditional E-value: 6.6e-10

round-2-all-seq-for-round-3-input-by-hmmalign 1 fgqEeGDAGlpPffpiFGKeGq 22

fgq +GD G+pP++p+FG+e++

g34966.t1 111 FGQKKGDTGYPPLSPLFGQEKD 132

8******************985 PP

== domain 6 score: 4.0 bits; conditional E-value: 0.038

round-2-all-seq-for-round-3-input-by-hmmalign 9 GlpPffpiFGK 19

G+ P++++FG+

g34966.t1 135 GYLPLSSLFGQ 145

899*******6 PP

== domain 7 score: 28.7 bits; conditional E-value: 6.9e-10

round-2-all-seq-for-round-3-input-by-hmmalign 1 fgqEeGDAGlpPffpiFGK 19

fgq ++DAG+pP++p+FG+

g34966.t1 143 FGQKKSDAGYPPLSPLFGQ 161

8*****************7 PP

== domain 8 score: 29.2 bits; conditional E-value: 5.1e-10

round-2-all-seq-for-round-3-input-by-hmmalign 2 gqEeGDAGlpPffpiFGKeG 21

gq +GDAG+pP++p+FG+e+

g34966.t1 160 GQDNGDAGYPPLSPLFGQEK 179

89***************995 PP

== domain 9 score: 33.2 bits; conditional E-value: 2.7e-11

round-2-all-seq-for-round-3-input-by-hmmalign 1 fgqEeGDAGlpPffpiFGKe 20

fgqE++DAG+pP++p+FG+e

g34966.t1 175 FGQEKSDAGYPPLSPLFGQE 194

9*****************97 PP

== domain 10 score: 30.4 bits; conditional E-value: 2.1e-10

round-2-all-seq-for-round-3-input-by-hmmalign 1 fgqEeGDAGlpPffpiFGK 19

fgqEe DAG+pP++p+FG+

g34966.t1 191 FGQEERDAGYPPLSPLFGQ 209

9*****************7 PP

== domain 11 score: 30.0 bits; conditional E-value: 2.8e-10

round-2-all-seq-for-round-3-input-by-hmmalign 1 fgqEeGDAGlpPffpiFGKeGq 22

fgq e DAG+pP++p+FG+e++

g34966.t1 207 FGQDERDAGYPPLSPLFGQEKS 228

8******************985 PP

== domain 12 score: 34.2 bits; conditional E-value: 1.3e-11

round-2-all-seq-for-round-3-input-by-hmmalign 1 fgqEeGDAGlpPffpiFGKeG 21

fgqE++DAG+pP++p+FG+e+

g34966.t1 223 FGQEKSDAGYPPLSPLFGQEK 243

9*****************995 PP

== domain 13 score: 29.7 bits; conditional E-value: 3.5e-10

round-2-all-seq-for-round-3-input-by-hmmalign 1 fgqEeGDAGlpPffpiFGK 19

fgqE+ DAG+pP++p+FG+

g34966.t1 239 FGQEKRDAGYPPLSPLFGQ 257

9*****************7 PP

== domain 14 score: 10.1 bits; conditional E-value: 0.00047

round-2-all-seq-for-round-3-input-by-hmmalign 2 gqEeGDAGlpPffpi 16

gq e DAG+pP++p+

g34966.t1 256 GQDEHDAGYPPLSPL 270

67788*********8 PP

== domain 15 score: 21.0 bits; conditional E-value: 1.9e-07

round-2-all-seq-for-round-3-input-by-hmmalign 3 qEeGDAGlp.P.ffpiFG 18

q+eGDAG++ P f++iFG

g34966.t1 274 QPEGDAGCVsPdFHMIFG 291

89***************9 PP

== domain 16 score: -0.0 bits; conditional E-value: 0.71

round-2-all-seq-for-round-3-input-by-hmmalign 4 EeGDAGl 10

E GDAG+

g34966.t1 335 EVGDAGY 341

77****9 PP

== domain 17 score: -0.0 bits; conditional E-value: 0.71

round-2-all-seq-for-round-3-input-by-hmmalign 4 EeGDAGl 10

E GDAG+

g34966.t1 352 EVGDAGY 358

77****9 PP

== domain 18 score: -0.0 bits; conditional E-value: 0.71

round-2-all-seq-for-round-3-input-by-hmmalign 4 EeGDAGl 10

E GDAG+

g34966.t1 369 EVGDAGY 375

77****9 PP

== domain 19 score: -0.0 bits; conditional E-value: 0.71

round-2-all-seq-for-round-3-input-by-hmmalign 4 EeGDAGl 10

E GDAG+

g34966.t1 386 EVGDAGY 392

77****9 PP

== domain 20 score: -0.0 bits; conditional E-value: 0.71

round-2-all-seq-for-round-3-input-by-hmmalign 4 EeGDAGl 10

E GDAG+

g34966.t1 403 EVGDAGY 409

77****9 PP

== domain 21 score: -0.0 bits; conditional E-value: 0.71

round-2-all-seq-for-round-3-input-by-hmmalign 4 EeGDAGl 10

E GDAG+

g34966.t1 420 EVGDAGY 426

77****9 PP

== domain 22 score: -0.0 bits; conditional E-value: 0.71

round-2-all-seq-for-round-3-input-by-hmmalign 4 EeGDAGl 10

E GDAG+

g34966.t1 437 EVGDAGY 443

77****9 PP

== domain 23 score: -1.7 bits; conditional E-value: 2.4

round-2-all-seq-for-round-3-input-by-hmmalign 6 GDAGl 10

GDAG+

g34966.t1 456 GDAGY 460

9***9 PP

== domain 24 score: 0.5 bits; conditional E-value: 0.47

round-2-all-seq-for-round-3-input-by-hmmalign 4 EeGDAGl 10

E GDAG+

g34966.t1 471 EVGDAGY 477

77****9 PP

== domain 25 score: 0.5 bits; conditional E-value: 0.47

round-2-all-seq-for-round-3-input-by-hmmalign 4 EeGDAGl 10

E GDAG+

g34966.t1 488 EVGDAGY 494

77****9 PP

== domain 26 score: -0.0 bits; conditional E-value: 0.71

round-2-all-seq-for-round-3-input-by-hmmalign 4 EeGDAGl 10

E GDAG+

g34966.t1 505 EVGDAGY 511

77****9 PP

>> g3343.t1

# score bias c-Evalue i-Evalue hmmfrom hmm to alifrom ali to envfrom env to acc

--- ------ ----- --------- --------- ------- ------- ------- ------- ------- ------- ----

1 ? -2.9 0.1 5.5 2.4e+04 5 9 .. 65 69 .. 65 69 .. 0.85

2 ! 21.8 0.0 1e-07 0.00044 4 23 .] 82 101 .. 81 101 .. 0.88

3 ! 22.5 0.1 6.1e-08 0.00026 2 19 .. 98 114 .. 98 115 .. 0.93

4 ! 22.2 0.2 7.9e-08 0.00034 4 19 .. 118 132 .. 117 133 .. 0.92

5 ! 28.0 0.1 1.2e-09 5e-06 4 22 .. 136 153 .. 135 153 .. 0.94

6 ! 27.0 0.2 2.5e-09 1.1e-05 2 19 .. 152 168 .. 152 169 .. 0.93

7 ! 29.8 0.1 3.2e-10 1.4e-06 4 23 .] 172 191 .. 171 191 .. 0.93

8 ! 9.6 0.1 0.00066 2.9 2 17 .. 188 202 .. 188 202 .. 0.92

9 ! 17.5 0.1 2.2e-06 0.0096 4 19 .. 208 222 .. 207 223 .. 0.92

10 ! 23.3 0.0 3.5e-08 0.00015 4 22 .. 226 243 .. 225 243 .. 0.94

11 ? 5.8 0.7 0.011 46 2 9 .. 242 249 .. 242 249 .. 0.92

Alignments for each domain:

== domain 1 score: -2.9 bits; conditional E-value: 5.5

round-2-all-seq-for-round-3-input-by-hmmalign 5 eGDAG 9

+GDA+

g3343.t1 65 KGDAS 69

7**96 PP

== domain 2 score: 21.8 bits; conditional E-value: 1e-07

round-2-all-seq-for-round-3-input-by-hmmalign 4 EeGDAGlpPffpiFGKeGq.d 23

Ee DA+l++++ +FGK+Gq +

g3343.t1 82 EEEDASLFNYY-VFGKAGQeV 101

677******87.*******77 PP

== domain 3 score: 22.5 bits; conditional E-value: 6.1e-08

round-2-all-seq-for-round-3-input-by-hmmalign 2 gqEeGDAGlpPffpiFGK 19

gqE GDA+l++++ +FGK

g3343.t1 98 GQEVGDASLFNYY-VFGK 114

8**********87.**** PP

== domain 4 score: 22.2 bits; conditional E-value: 7.9e-08

round-2-all-seq-for-round-3-input-by-hmmalign 4 EeGDAGlpPffpiFGK 19

EeGDA+l++++ +FGK

g3343.t1 118 EEGDASLFNYY-VFGK 132

9********87.**** PP

== domain 5 score: 28.0 bits; conditional E-value: 1.2e-09

round-2-all-seq-for-round-3-input-by-hmmalign 4 EeGDAGlpPffpiFGKeGq 22

EeGDA+l++++ +FGK+Gq

g3343.t1 136 EEGDASLFNYY-VFGKAGQ 153

9********87.******8 PP

== domain 6 score: 27.0 bits; conditional E-value: 2.5e-09

round-2-all-seq-for-round-3-input-by-hmmalign 2 gqEeGDAGlpPffpiFGK 19

gqEeGDA+l++++ +FGK

g3343.t1 152 GQEEGDASLFNYY-VFGK 168

8**********87.**** PP

== domain 7 score: 29.8 bits; conditional E-value: 3.2e-10

round-2-all-seq-for-round-3-input-by-hmmalign 4 EeGDAGlpPffpiFGKeGq.d 23

EeGDA+l++++ +FGK+Gq +

g3343.t1 172 EEGDASLFNYY-VFGKAGQeV 191

9********87.*******77 PP

== domain 8 score: 9.6 bits; conditional E-value: 0.00066

round-2-all-seq-for-round-3-input-by-hmmalign 2 gqEeGDAGlpPffpiF 17

gqE DA+l++++ +F

g3343.t1 188 GQEVDDASLFNYY-VF 202

89*********87.88 PP

== domain 9 score: 17.5 bits; conditional E-value: 2.2e-06

round-2-all-seq-for-round-3-input-by-hmmalign 4 EeGDAGlpPffpiFGK 19

Ee DA+l++++ +FGK

g3343.t1 208 EEADASLFNYY-VFGK 222

9********87.**** PP

== domain 10 score: 23.3 bits; conditional E-value: 3.5e-08

round-2-all-seq-for-round-3-input-by-hmmalign 4 EeGDAGlpPffpiFGKeGq 22

Ee DA+l++++ +FGK+Gq

g3343.t1 226 EEADASLFNYY-VFGKAGQ 243

9********87.******8 PP

== domain 11 score: 5.8 bits; conditional E-value: 0.011

round-2-all-seq-for-round-3-input-by-hmmalign 2 gqEeGDAG 9

gqEeGDA+

g3343.t1 242 GQEEGDAS 249

8******6 PP

>> g7437.t1

# score bias c-Evalue i-Evalue hmmfrom hmm to alifrom ali to envfrom env to acc

--- ------ ----- --------- --------- ------- ------- ------- ------- ------- ------- ----

1 ? 3.5 0.0 0.054 2.3e+02 4 11 .. 62 69 .. 61 69 .. 0.88

2 ? -0.2 0.1 0.78 3.4e+03 16 19 .. 75 78 .. 75 79 .. 0.86

3 ! 32.0 1.0 6.7e-11 2.9e-07 7 23 .] 99 116 .. 98 116 .. 0.94

4 ! 35.8 0.3 4.2e-12 1.8e-08 4 23 .] 120 140 .. 117 140 .. 0.89

5 ! 31.7 0.3 8.1e-11 3.5e-07 6 23 .] 145 163 .. 143 163 .. 0.92

6 ! 33.0 0.3 3.3e-11 1.4e-07 6 23 .] 168 186 .. 166 186 .. 0.91

7 ! 32.4 0.1 4.9e-11 2.1e-07 6 22 .. 190 206 .. 188 208 .. 0.90

Alignments for each domain:

== domain 1 score: 3.5 bits; conditional E-value: 0.054

round-2-all-seq-for-round-3-input-by-hmmalign 4 EeGDAGlp 11

++GDA+l+

g7437.t1 62 PKGDASLF 69

79****98 PP

== domain 2 score: -0.2 bits; conditional E-value: 0.78

round-2-all-seq-for-round-3-input-by-hmmalign 16 iFGK 19

+FGK

g7437.t1 75 VFGK 78

8*** PP

== domain 3 score: 32.0 bits; conditional E-value: 6.7e-11

round-2-all-seq-for-round-3-input-by-hmmalign 7 DAGlpPffpiFGKeGq.d 23

D++l+Pff+iFGKeGq +

g7437.t1 99 DDMLMPFFWIFGKEGQqQ 116

9***************66 PP

== domain 4 score: 35.8 bits; conditional E-value: 4.2e-12

round-2-all-seq-for-round-3-input-by-hmmalign 4 EeGDAGlpPffpiFGKeGq.d 23

E +D++l+Pff+iFGKeGq +

g7437.t1 120 ESSDDMLMPFFWIFGKEGQqQ 140

669****************66 PP

== domain 5 score: 31.7 bits; conditional E-value: 8.1e-11

round-2-all-seq-for-round-3-input-by-hmmalign 6 GDAGlpPffpiFGKeGq.d 23

+D++l Pff+iFGKeGq +

g7437.t1 145 SDDMLLPFFWIFGKEGQqQ 163

8****************66 PP

== domain 6 score: 33.0 bits; conditional E-value: 3.3e-11

round-2-all-seq-for-round-3-input-by-hmmalign 6 GDAGlpPffpiFGKeGq.d 23

+D++l+Pff+iFGK++q +

g7437.t1 168 SDDMLMPFFWIFGKQQQqQ 186

8***************966 PP

== domain 7 score: 32.4 bits; conditional E-value: 4.9e-11

round-2-all-seq-for-round-3-input-by-hmmalign 6 GDAGlpPffpiFGKeGq 22

+D++l+Pff++FGK+G+

g7437.t1 190 SDDMLMPFFWVFGKQGD 206

8***************8 PP

>> g24918.t1

# score bias c-Evalue i-Evalue hmmfrom hmm to alifrom ali to envfrom env to acc

--- ------ ----- --------- --------- ------- ------- ------- ------- ------- ------- ----

1 ! 24.8 0.1 1.2e-08 5.2e-05 5 19 .. 49 63 .. 49 64 .. 0.95

2 ! 21.9 0.8 9.9e-08 0.00042 7 21 .. 67 81 .. 65 81 .. 0.94

3 ! 22.7 2.2 5.2e-08 0.00022 1 20 [. 77 97 .. 77 98 .. 0.95

4 ! 16.5 0.2 4.7e-06 0.02 4 20 .. 100 117 .. 99 118 .. 0.94

5 ! 16.5 0.2 4.7e-06 0.02 4 20 .. 120 137 .. 119 138 .. 0.94

6 ! 16.5 0.2 4.7e-06 0.02 4 20 .. 140 157 .. 139 158 .. 0.94

7 ! 16.5 0.2 4.7e-06 0.02 4 20 .. 160 177 .. 159 178 .. 0.94

8 ! 16.7 0.1 4.2e-06 0.018 4 20 .. 180 197 .. 179 199 .. 0.94

9 ! 8.2 0.3 0.0018 7.8 3 19 .. 210 218 .. 208 219 .. 0.83

Alignments for each domain:

== domain 1 score: 24.8 bits; conditional E-value: 1.2e-08

round-2-all-seq-for-round-3-input-by-hmmalign 5 eGDAGlpPffpiFGK 19

+GDAG+pP++p+FG+

g24918.t1 49 KGDAGYPPLSPLFGQ 63

8*************6 PP

== domain 2 score: 21.9 bits; conditional E-value: 9.9e-08

round-2-all-seq-for-round-3-input-by-hmmalign 7 DAGlpPffpiFGKeG 21

DAG+pP++p+FG+e+

g24918.t1 67 DAGYPPLSPLFGQEK 81

************995 PP

== domain 3 score: 22.7 bits; conditional E-value: 5.2e-08

round-2-all-seq-for-round-3-input-by-hmmalign 1 fgqEeGDAGlp.P.ffpiFGKe 20

fgqE+GDAG + + ++ +FGK

g24918.t1 77 FGQEKGDAGVYgFiPH-VFGKG 97

9***************.****5 PP

== domain 4 score: 16.5 bits; conditional E-value: 4.7e-06

round-2-all-seq-for-round-3-input-by-hmmalign 4 EeGDAGlp.P.ffpiFGKe 20

E+GDAG + + ++ +FGK

g24918.t1 100 ENGDAGVYgFiPH-VFGKG 117

9************.****5 PP

== domain 5 score: 16.5 bits; conditional E-value: 4.7e-06

round-2-all-seq-for-round-3-input-by-hmmalign 4 EeGDAGlp.P.ffpiFGKe 20

E+GDAG + + ++ +FGK

g24918.t1 120 ENGDAGVYgFiPH-VFGKG 137

9************.****5 PP

== domain 6 score: 16.5 bits; conditional E-value: 4.7e-06

round-2-all-seq-for-round-3-input-by-hmmalign 4 EeGDAGlp.P.ffpiFGKe 20

E+GDAG + + ++ +FGK

g24918.t1 140 ENGDAGVYgFiPH-VFGKG 157

9************.****5 PP

== domain 7 score: 16.5 bits; conditional E-value: 4.7e-06

round-2-all-seq-for-round-3-input-by-hmmalign 4 EeGDAGlp.P.ffpiFGKe 20

E+GDAG + + ++ +FGK

g24918.t1 160 ENGDAGVYgFiPH-VFGKG 177

9************.****5 PP

== domain 8 score: 16.7 bits; conditional E-value: 4.2e-06

round-2-all-seq-for-round-3-input-by-hmmalign 4 EeGDAGlp.P.ffpiFGKe 20

E+GDAG + + ++ +FGK

g24918.t1 180 ENGDAGVYgFiPH-VFGKG 197

9************.****5 PP

== domain 9 score: 8.2 bits; conditional E-value: 0.0018

round-2-all-seq-for-round-3-input-by-hmmalign 3 qEeGDAGlpPffpiFGK 19

+ ++P +iFGK

g24918.t1 210 P------YFP--MIFGK 218

6......999..9**** PP

>> g24919.t1

# score bias c-Evalue i-Evalue hmmfrom hmm to alifrom ali to envfrom env to acc

--- ------ ----- --------- --------- ------- ------- ------- ------- ------- ------- ----

1 ! 35.4 0.2 5.6e-12 2.4e-08 1 23 [] 83 106 .. 83 106 .. 0.90

2 ! 44.3 0.6 9e-15 3.9e-11 1 23 [] 115 139 .. 115 139 .. 0.95

3 ! 36.7 0.5 2.2e-12 9.6e-09 1 23 [] 154 178 .. 154 178 .. 0.94

4 ? -1.5 1.4 2 8.5e+03 18 23 .] 190 196 .. 190 196 .. 0.90

Alignments for each domain:

== domain 1 score: 35.4 bits; conditional E-value: 5.6e-12

round-2-all-seq-for-round-3-input-by-hmmalign 1 fgqEeGDAGlpP.ffpiFGKeGq.d 23

++qEe+D++l++ +f iFGKeG+ d

g24919.t1 83 RNQEESDGMLVFpLF-IFGKEGSqD 106

68*********5599.******977 PP

== domain 2 score: 44.3 bits; conditional E-value: 9e-15

round-2-all-seq-for-round-3-input-by-hmmalign 1 fgqEeGDAGlpP.ffpiFGKeGq.d 23

++qEe+D++l+P ff+iFGKeG+ d

g24919.t1 115 RDQEESDGMLIPpFFVIFGKEGCqD 139

69*********99**********77 PP

== domain 3 score: 36.7 bits; conditional E-value: 2.2e-12

round-2-all-seq-for-round-3-input-by-hmmalign 1 fgqEeGDAGlpP.ffpiFGKeGq.d 23

++qEe+D+ l+P ff iFGKeG+ d

g24919.t1 154 RDQEESDGILVPpFFLIFGKEGSqD 178

69*********99*********977 PP

== domain 4 score: -1.5 bits; conditional E-value: 2

round-2-all-seq-for-round-3-input-by-hmmalign 18 GKeGq.d 23

GKe+q d

g24919.t1 190 GKEQQgD 196

9***988 PP

>> g28070.t1

# score bias c-Evalue i-Evalue hmmfrom hmm to alifrom ali to envfrom env to acc

--- ------ ----- --------- --------- ------- ------- ------- ------- ------- ------- ----

1 ! 30.2 0.3 2.4e-10 1e-06 1 19 [. 15 33 .. 15 34 .. 0.93

2 ! 32.4 2.6 4.8e-11 2.1e-07 1 21 [. 31 51 .. 31 51 .. 0.96

3 ! 39.2 3.7 3.6e-13 1.5e-09 1 21 [. 47 67 .. 47 68 .. 0.96

4 ? 4.5 2.2 0.027 1.2e+02 4 10 .. 66 72 .. 66 79 .. 0.73

5 ? 0.2 1.6 0.6 2.6e+03 6 19 .. 92 102 .. 92 103 .. 0.56

6 ? -0.6 1.0 1.1 4.6e+03 16 19 .. 123 126 .. 116 127 .. 0.72

7 ? 0.7 0.1 0.41 1.8e+03 6 10 .. 140 144 .. 138 144 .. 0.90

8 ? -0.2 0.1 0.82 3.5e+03 4 11 .. 155 162 .. 155 165 .. 0.86

Alignments for each domain:

== domain 1 score: 30.2 bits; conditional E-value: 2.4e-10

round-2-all-seq-for-round-3-input-by-hmmalign 1 fgqEeGDAGlpPffpiFGK 19

++q +GDAG+pP++p+FG+

g28070.t1 15 RNQDNGDAGYPPLSPLFGQ 33

68****************7 PP

== domain 2 score: 32.4 bits; conditional E-value: 4.8e-11

round-2-all-seq-for-round-3-input-by-hmmalign 1 fgqEeGDAGlpPffpiFGKeG 21

fgq +GDAG+pP++p+FG+e+

g28070.t1 31 FGQDNGDAGYPPLSPLFGQEK 51

9*****************995 PP

== domain 3 score: 39.2 bits; conditional E-value: 3.6e-13

round-2-all-seq-for-round-3-input-by-hmmalign 1 fgqEeGDAGlpPffpiFGKeG 21

fgqE+GDAG+pP++p+FGKe+

g28070.t1 47 FGQEKGDAGYPPLSPLFGKEK 67

9******************97 PP

== domain 4 score: 4.5 bits; conditional E-value: 0.027

round-2-all-seq-for-round-3-input-by-hmmalign 4 EeGDAGl 10

E+GDAG+

g28070.t1 66 EKGDAGY 72

9*****7 PP

== domain 5 score: 0.2 bits; conditional E-value: 0.6

round-2-all-seq-for-round-3-input-by-hmmalign 6 GDAGlpPffpiFGK 19

GDAG+ +FGK

g28070.t1 92 GDAGYNG---LFGK 102

7777522...4666 PP

== domain 6 score: -0.6 bits; conditional E-value: 1.1

round-2-all-seq-for-round-3-input-by-hmmalign 16 iFGK 19

+FGK

g28070.t1 123 LFGK 126

6888 PP

== domain 7 score: 0.7 bits; conditional E-value: 0.41

round-2-all-seq-for-round-3-input-by-hmmalign 6 GDAGl 10

GDAG+

g28070.t1 140 GDAGY 144

9***9 PP

== domain 8 score: -0.2 bits; conditional E-value: 0.82

round-2-all-seq-for-round-3-input-by-hmmalign 4 EeGDAGlp 11

E GD G++

g28070.t1 155 EVGDTGYY 162

77****97 PP

>> g26217.t1

# score bias c-Evalue i-Evalue hmmfrom hmm to alifrom ali to envfrom env to acc

--- ------ ----- --------- --------- ------- ------- ------- ------- ------- ------- ----

1 ! 19.6 0.0 5e-07 0.0022 4 19 .. 56 71 .. 55 76 .. 0.93

2 ! 30.6 0.6 1.8e-10 7.7e-07 1 23 [] 91 115 .. 91 115 .. 0.94

3 ! 32.1 1.0 6.2e-11 2.7e-07 4 23 .] 116 137 .. 114 137 .. 0.93

Alignments for each domain:

== domain 1 score: 19.6 bits; conditional E-value: 5e-07

round-2-all-seq-for-round-3-input-by-hmmalign 4 EeGDAGlpPffpiFGK 19

E+GDA+++P +FGK

g26217.t1 56 EHGDAMIFPITRLFGK 71

9********99****9 PP

== domain 2 score: 30.6 bits; conditional E-value: 1.8e-10

round-2-all-seq-for-round-3-input-by-hmmalign 1 fgqEeGDAGlpP.ffpiFGKeGq.d 23

fg Ee DAGl+P ++++FGK G+ +

g26217.t1 91 FGEEEDDAGLFPiPMTVFGKGGMnV 115

899******************9866 PP

== domain 3 score: 32.1 bits; conditional E-value: 6.2e-11

round-2-all-seq-for-round-3-input-by-hmmalign 4 EeGDAGlpP.ffpiFGKeGq.d 23

EeGDAGl+P ++++FGK G+ d

g26217.t1 116 EEGDAGLFPiPMTVFGKGGDqD 137

9******************977 PP

>> g53356.t1

# score bias c-Evalue i-Evalue hmmfrom hmm to alifrom ali to envfrom env to acc

--- ------ ----- --------- --------- ------- ------- ------- ------- ------- ------- ----

1 ? -1.5 0.2 2 8.4e+03 6 10 .. 65 69 .. 64 69 .. 0.89

2 ! 24.8 0.1 1.2e-08 5.2e-05 6 22 .. 70 87 .. 70 89 .. 0.91

3 ! 13.4 0.0 4.3e-05 0.19 1 18 [. 90 108 .. 90 110 .. 0.94

4 ! 11.5 0.0 0.00017 0.72 1 18 [. 114 132 .. 114 135 .. 0.91

5 ! 13.4 0.0 4.3e-05 0.19 1 18 [. 138 156 .. 138 158 .. 0.94

6 ! 8.9 0.0 0.0011 4.8 4 18 .. 165 180 .. 162 182 .. 0.89

7 ! 8.9 0.0 0.0011 4.8 4 18 .. 189 204 .. 186 206 .. 0.89

8 ? 2.5 0.0 0.11 4.8e+02 1 9 [. 210 218 .. 210 219 .. 0.91

Alignments for each domain:

== domain 1 score: -1.5 bits; conditional E-value: 2

round-2-all-seq-for-round-3-input-by-hmmalign 6 GDAGl 10

GDAG+

g53356.t1 65 GDAGI 69

9***7 PP

== domain 2 score: 24.8 bits; conditional E-value: 1.2e-08

round-2-all-seq-for-round-3-input-by-hmmalign 6 GDAGlpP.ffpiFGKeGq 22

GD+G+pP ++++FGK++

g53356.t1 70 GDDGIPPfWLTLFGKQQA 87

9**************975 PP

== domain 3 score: 13.4 bits; conditional E-value: 4.3e-05

round-2-all-seq-for-round-3-input-by-hmmalign 1 fgqEeGDAGlpP.ffpiFG 18

f++E+GDAG P + ++FG

g53356.t1 90 FNSEKGDAGMAPmWVTVFG 108

89*******87*******9 PP

== domain 4 score: 11.5 bits; conditional E-value: 0.00017

round-2-all-seq-for-round-3-input-by-hmmalign 1 fgqEeGDAGlpP.ffpiFG 18

f++E+GDAG P + ++FG

g53356.t1 114 FNSEKGDAGMAPvWGTVFG 132

89*******7799999998 PP

== domain 5 score: 13.4 bits; conditional E-value: 4.3e-05

round-2-all-seq-for-round-3-input-by-hmmalign 1 fgqEeGDAGlpP.ffpiFG 18

f++E+GDAG P + ++FG

g53356.t1 138 FNSEKGDAGMAPmWVTVFG 156

89*******87*******9 PP

== domain 6 score: 8.9 bits; conditional E-value: 0.0011

round-2-all-seq-for-round-3-input-by-hmmalign 4 EeGDAGlpP.ffpiFG 18

E+GDAG P + ++FG

g53356.t1 165 EKGDAGMAPmWVTVFG 180

9*****87*******9 PP

== domain 7 score: 8.9 bits; conditional E-value: 0.0011

round-2-all-seq-for-round-3-input-by-hmmalign 4 EeGDAGlpP.ffpiFG 18

E+GDAG P + ++FG

g53356.t1 189 EKGDAGMAPvWVTVFG 204

9*****87*******9 PP

== domain 8 score: 2.5 bits; conditional E-value: 0.11

round-2-all-seq-for-round-3-input-by-hmmalign 1 fgqEeGDAG 9

f++ +GDA+

g53356.t1 210 FNSKKGDAS 218

899*****7 PP

>> g33422.t1

# score bias c-Evalue i-Evalue hmmfrom hmm to alifrom ali to envfrom env to acc

--- ------ ----- --------- --------- ------- ------- ------- ------- ------- ------- ----

1 ? -2.7 0.1 4.9 2.1e+04 9 12 .. 27 30 .. 27 32 .. 0.76

2 ! 31.0 0.0 1.3e-10 5.7e-07 2 22 .. 56 77 .. 55 79 .. 0.91

3 ! 10.0 0.0 0.00051 2.2 6 13 .. 85 93 .. 80 94 .. 0.77

4 ! 29.0 0.0 5.9e-10 2.5e-06 1 20 [. 97 117 .. 97 117 .. 0.96

Alignments for each domain:

== domain 1 score: -2.7 bits; conditional E-value: 4.9

round-2-all-seq-for-round-3-input-by-hmmalign 9 GlpP 12

GlpP

g33422.t1 27 GLPP 30

89*9 PP

== domain 2 score: 31.0 bits; conditional E-value: 1.3e-10

round-2-all-seq-for-round-3-input-by-hmmalign 2 gqEeGDAGlpP.ffpiFGKeGq 22

++E+GDAG+pP ++++ GK+++

g33422.t1 56 DSERGDAGIPPfWLTLVGKQRT 77

89******************87 PP

== domain 3 score: 10.0 bits; conditional E-value: 0.00051

round-2-all-seq-for-round-3-input-by-hmmalign 6 GDAGlpP.f 13

GDAGlpP +

g33422.t1 85 GDAGLPPmW 93

9******66 PP

== domain 4 score: 29.0 bits; conditional E-value: 5.9e-10

round-2-all-seq-for-round-3-input-by-hmmalign 1 fgqEeGDAGlpP.ffpiFGKe 20

fg+E+GDAG+pP ++++ GK+

g33422.t1 97 FGSERGDAGIPPfWLTLIGKH 117

8*******************6 PP

>> g46938.t1

# score bias c-Evalue i-Evalue hmmfrom hmm to alifrom ali to envfrom env to acc

--- ------ ----- --------- --------- ------- ------- ------- ------- ------- ------- ----

1 ! 7.4 0.0 0.0034 14 7 18 .. 23 33 .. 20 33 .. 0.92

2 ! 19.3 0.1 6.1e-07 0.0026 4 19 .. 41 57 .. 39 57 .. 0.88

3 ! 22.6 0.4 5.8e-08 0.00025 5 20 .. 64 79 .. 64 80 .. 0.87

4 ! 13.8 0.8 3.2e-05 0.14 7 20 .. 80 94 .. 80 94 .. 0.87

5 ! 10.9 6.2 0.00026 1.1 5 20 .. 100 115 .. 99 116 .. 0.90

6 ! 7.8 4.5 0.0025 11 7 20 .. 124 137 .. 120 137 .. 0.86

7 ? -1.5 0.1 2 8.7e+03 5 9 .. 144 148 .. 143 148 .. 0.85

Alignments for each domain:

== domain 1 score: 7.4 bits; conditional E-value: 0.0034

round-2-all-seq-for-round-3-input-by-hmmalign 7 DAGlpP.ffpiFG 18

DAGl+P ++ FG

g46938.t1 23 DAGLIPvPM--FG 33

******999..98 PP

== domain 2 score: 19.3 bits; conditional E-value: 6.1e-07

round-2-all-seq-for-round-3-input-by-hmmalign 4 EeGDAGlp.PffpiFGK 19

+eGDAG++ P + iFGK

g46938.t1 41 PEGDAGIIdPILLIFGK 57

59******99667***9 PP

== domain 3 score: 22.6 bits; conditional E-value: 5.8e-08

round-2-all-seq-for-round-3-input-by-hmmalign 5 eGDAGlpP.ffpiFGKe 20

+GDAG +P f ++FGKe

g46938.t1 64 NGDAGFVPnF-SVFGKE 79

7*******55.6****8 PP

== domain 4 score: 13.8 bits; conditional E-value: 3.2e-05

round-2-all-seq-for-round-3-input-by-hmmalign 7 DAGlp.PffpiFGKe 20

DA l+ P fpiFGKe

g46938.t1 80 DAALFiPSFPIFGKE 94

777888*88*****8 PP

== domain 5 score: 10.9 bits; conditional E-value: 0.00026

round-2-all-seq-for-round-3-input-by-hmmalign 5 eGDAGlpP.ffpiFGKe 20

+GDAG + ffp FGK+

g46938.t1 100 KGDAG-FDiFFPFFGKA 115

9****.99999*****7 PP

== domain 6 score: 7.8 bits; conditional E-value: 0.0025

round-2-all-seq-for-round-3-input-by-hmmalign 7 DAGlpP.ffpiFGKe 20

DAG + ffp FGK+

g46938.t1 124 DAG-FDiFFPFFGKQ 137

888.88899*****8 PP

== domain 7 score: -1.5 bits; conditional E-value: 2

round-2-all-seq-for-round-3-input-by-hmmalign 5 eGDAG 9

+GD+G

g46938.t1 144 NGDDG 148

8**98 PP

>> g15288.t1

# score bias c-Evalue i-Evalue hmmfrom hmm to alifrom ali to envfrom env to acc

--- ------ ----- --------- --------- ------- ------- ------- ------- ------- ------- ----

1 ? 4.7 0.0 0.023 1e+02 15 20 .. 35 40 .. 32 41 .. 0.84

2 ? 4.3 0.0 0.03 1.3e+02 13 20 .. 47 53 .. 44 53 .. 0.84

3 ? 1.6 0.0 0.21 9.1e+02 5 20 .. 60 73 .. 56 74 .. 0.77

4 ? 4.3 0.0 0.031 1.3e+02 15 20 .. 80 85 .. 77 85 .. 0.84

5 ? 4.0 0.0 0.037 1.6e+02 15 20 .. 93 98 .. 92 99 .. 0.87

6 ? 4.4 0.0 0.029 1.2e+02 15 20 .. 106 111 .. 102 111 .. 0.84

7 ? 4.3 0.2 0.032 1.4e+02 15 20 .. 119 124 .. 118 124 .. 0.90

8 ? 4.4 0.0 0.029 1.2e+02 15 20 .. 133 138 .. 129 138 .. 0.84

9 ? 4.0 0.0 0.037 1.6e+02 15 20 .. 146 151 .. 145 152 .. 0.87

10 ? 4.3 0.2 0.032 1.4e+02 15 20 .. 159 164 .. 158 164 .. 0.90

11 ? 5.1 0.0 0.017 73 15 20 .. 173 178 .. 169 178 .. 0.85

12 ? 5.1 0.0 0.017 73 15 20 .. 187 192 .. 183 192 .. 0.85

13 ? 4.4 0.0 0.029 1.3e+02 13 20 .. 200 206 .. 196 206 .. 0.85

14 ? 0.6 0.2 0.45 1.9e+03 6 20 .. 213 225 .. 212 226 .. 0.70

15 ? 4.3 0.2 0.032 1.4e+02 15 20 .. 233 238 .. 232 238 .. 0.90

16 ? -2.0 0.1 3 1.3e+04 6 13 .. 245 254 .. 244 254 .. 0.81

Alignments for each domain:

== domain 1 score: 4.7 bits; conditional E-value: 0.023

round-2-all-seq-for-round-3-input-by-hmmalign 15 piFGKe 20

p+FGK+

g15288.t1 35 PVFGKA 40

9****7 PP

== domain 2 score: 4.3 bits; conditional E-value: 0.03

round-2-all-seq-for-round-3-input-by-hmmalign 13 ffpiFGKe 20

+ p+FGK+

g15288.t1 47 W-PVFGKA 53

4.9****6 PP

== domain 3 score: 1.6 bits; conditional E-value: 0.21

round-2-all-seq-for-round-3-input-by-hmmalign 5 eGDAGlp.PffpiFGKe 20

+GD G++ P FGK+

g15288.t1 60 RGDSGYVvP---CFGKA 73

688899888...37886 PP

== domain 4 score: 4.3 bits; conditional E-value: 0.031

round-2-all-seq-for-round-3-input-by-hmmalign 15 piFGKe 20

p+FGK+

g15288.t1 80 PVFGKA 85

9****6 PP

== domain 5 score: 4.0 bits; conditional E-value: 0.037

round-2-all-seq-for-round-3-input-by-hmmalign 15 piFGKe 20

p+FGK+

g15288.t1 93 PVFGKA 98

9****7 PP

== domain 6 score: 4.4 bits; conditional E-value: 0.029

round-2-all-seq-for-round-3-input-by-hmmalign 15 piFGKe 20

p+FGK+

g15288.t1 106 PVFGKA 111

9****6 PP

== domain 7 score: 4.3 bits; conditional E-value: 0.032

round-2-all-seq-for-round-3-input-by-hmmalign 15 piFGKe 20

p+FGKe

g15288.t1 119 PVFGKE 124

9****8 PP

== domain 8 score: 4.4 bits; conditional E-value: 0.029

round-2-all-seq-for-round-3-input-by-hmmalign 15 piFGKe 20

p+FGK+

g15288.t1 133 PVFGKA 138

9****6 PP

== domain 9 score: 4.0 bits; conditional E-value: 0.037

round-2-all-seq-for-round-3-input-by-hmmalign 15 piFGKe 20

p+FGK+

g15288.t1 146 PVFGKA 151

9****7 PP

== domain 10 score: 4.3 bits; conditional E-value: 0.032

round-2-all-seq-for-round-3-input-by-hmmalign 15 piFGKe 20

p+FGKe

g15288.t1 159 PVFGKE 164

9****8 PP

== domain 11 score: 5.1 bits; conditional E-value: 0.017

round-2-all-seq-for-round-3-input-by-hmmalign 15 piFGKe 20

p+FGKe

g15288.t1 173 PVFGKE 178

9****8 PP

== domain 12 score: 5.1 bits; conditional E-value: 0.017

round-2-all-seq-for-round-3-input-by-hmmalign 15 piFGKe 20

p+FGKe

g15288.t1 187 PVFGKE 192

9****8 PP

== domain 13 score: 4.4 bits; conditional E-value: 0.029

round-2-all-seq-for-round-3-input-by-hmmalign 13 ffpiFGKe 20

+ p+FGK+

g15288.t1 200 W-PVFGKA 206

5.9****6 PP

== domain 14 score: 0.6 bits; conditional E-value: 0.45

round-2-all-seq-for-round-3-input-by-hmmalign 6 GDAGlp.PffpiFGKe 20

GD G++ P FGK+

g15288.t1 213 GDSGYVvP---CFGKA 225

67777777...38886 PP

== domain 15 score: 4.3 bits; conditional E-value: 0.032

round-2-all-seq-for-round-3-input-by-hmmalign 15 piFGKe 20

p+FGKe

g15288.t1 233 PVFGKE 238

9****8 PP

== domain 16 score: -2.0 bits; conditional E-value: 3

round-2-all-seq-for-round-3-input-by-hmmalign 6 GDAGlp.P.f 13

GD G++ P f

g15288.t1 245 GDSGYVvPcF 254

8999998966 PP

>> g18055.t1

# score bias c-Evalue i-Evalue hmmfrom hmm to alifrom ali to envfrom env to acc

--- ------ ----- --------- --------- ------- ------- ------- ------- ------- ------- ----

1 ! 7.8 0.0 0.0025 11 7 20 .. 50 63 .. 49 64 .. 0.86

2 ? 5.5 0.1 0.013 57 14 20 .. 71 77 .. 71 78 .. 0.92

3 ! 6.0 0.1 0.0094 40 14 20 .. 85 91 .. 85 92 .. 0.92

4 ? 5.5 0.1 0.013 57 14 20 .. 99 105 .. 99 106 .. 0.92

5 ? -0.6 0.2 1.1 4.6e+03 14 18 .. 113 117 .. 113 117 .. 0.92

6 ? -0.6 0.2 1.1 4.6e+03 14 18 .. 127 131 .. 127 131 .. 0.92

7 ? -0.6 0.2 1.1 4.6e+03 14 18 .. 141 145 .. 141 145 .. 0.92

8 ? -0.6 0.2 1.1 4.6e+03 14 18 .. 155 159 .. 155 159 .. 0.92

9 ? -0.6 0.2 1.1 4.6e+03 14 18 .. 169 173 .. 169 173 .. 0.92

10 ? -0.6 0.2 1.1 4.6e+03 14 18 .. 183 187 .. 183 187 .. 0.92

11 ? -0.6 0.2 1.1 4.6e+03 14 18 .. 197 201 .. 197 201 .. 0.92

12 ? -0.6 0.2 1.1 4.6e+03 14 18 .. 211 215 .. 211 215 .. 0.92

13 ? -0.6 0.2 1.1 4.6e+03 14 18 .. 225 229 .. 225 229 .. 0.92

14 ! 6.1 0.1 0.0084 36 14 20 .. 239 245 .. 239 246 .. 0.91

15 ? 4.8 0.1 0.022 95 14 20 .. 253 259 .. 253 259 .. 0.89

16 ! 9.8 0.1 0.00058 2.5 9 20 .. 263 274 .. 262 275 .. 0.77

17 ! 6.0 0.1 0.0094 40 14 20 .. 282 288 .. 282 289 .. 0.92

18 ? 5.3 0.1 0.015 66 14 20 .. 296 302 .. 296 302 .. 0.92

19 ! 6.0 0.1 0.0094 40 14 20 .. 310 316 .. 310 317 .. 0.92

20 ! 6.0 0.1 0.0094 40 14 20 .. 324 330 .. 324 331 .. 0.92

21 ! 6.0 0.1 0.0094 40 14 20 .. 338 344 .. 338 345 .. 0.92

22 ! 6.0 0.1 0.0094 40 14 20 .. 352 358 .. 352 359 .. 0.92

23 ? 4.8 0.1 0.022 95 14 20 .. 366 372 .. 366 372 .. 0.89

24 ! 12.7 0.0 7.5e-05 0.32 7 20 .. 374 387 .. 374 388 .. 0.91

25 ? -0.6 0.2 1.1 4.6e+03 14 18 .. 395 399 .] 395 399 .] 0.92

Alignments for each domain:

== domain 1 score: 7.8 bits; conditional E-value: 0.0025

round-2-all-seq-for-round-3-input-by-hmmalign 7 DAGlp.P.ffpiFGKe 20

D++l+ P + +FGK+

g18055.t1 50 DGSLVrPvP--VFGKQ 63

999999955..****8 PP

== domain 2 score: 5.5 bits; conditional E-value: 0.013

round-2-all-seq-for-round-3-input-by-hmmalign 14 fpiFGKe 20

+p+FGK+

g18055.t1 71 YPVFGKQ 77

79****8 PP

== domain 3 score: 6.0 bits; conditional E-value: 0.0094

round-2-all-seq-for-round-3-input-by-hmmalign 14 fpiFGKe 20

+piFGK+

g18055.t1 85 YPIFGKQ 91

79****8 PP

== domain 4 score: 5.5 bits; conditional E-value: 0.013

round-2-all-seq-for-round-3-input-by-hmmalign 14 fpiFGKe 20

+p+FGK+

g18055.t1 99 YPVFGKQ 105

79****8 PP

== domain 5 score: -0.6 bits; conditional E-value: 1.1

round-2-all-seq-for-round-3-input-by-hmmalign 14 fpiFG 18

+piFG

g18055.t1 113 YPIFG 117

79**9 PP

== domain 6 score: -0.6 bits; conditional E-value: 1.1

round-2-all-seq-for-round-3-input-by-hmmalign 14 fpiFG 18

+piFG

g18055.t1 127 YPIFG 131

79**9 PP

== domain 7 score: -0.6 bits; conditional E-value: 1.1

round-2-all-seq-for-round-3-input-by-hmmalign 14 fpiFG 18

+piFG

g18055.t1 141 YPIFG 145

79**9 PP

== domain 8 score: -0.6 bits; conditional E-value: 1.1

round-2-all-seq-for-round-3-input-by-hmmalign 14 fpiFG 18

+piFG

g18055.t1 155 YPIFG 159

79**9 PP

== domain 9 score: -0.6 bits; conditional E-value: 1.1

round-2-all-seq-for-round-3-input-by-hmmalign 14 fpiFG 18

+piFG

g18055.t1 169 YPIFG 173

79**9 PP

== domain 10 score: -0.6 bits; conditional E-value: 1.1

round-2-all-seq-for-round-3-input-by-hmmalign 14 fpiFG 18

+piFG

g18055.t1 183 YPIFG 187

79**9 PP

== domain 11 score: -0.6 bits; conditional E-value: 1.1

round-2-all-seq-for-round-3-input-by-hmmalign 14 fpiFG 18

+piFG

g18055.t1 197 YPIFG 201

79**9 PP

== domain 12 score: -0.6 bits; conditional E-value: 1.1

round-2-all-seq-for-round-3-input-by-hmmalign 14 fpiFG 18

+piFG

g18055.t1 211 YPIFG 215

79**9 PP

== domain 13 score: -0.6 bits; conditional E-value: 1.1

round-2-all-seq-for-round-3-input-by-hmmalign 14 fpiFG 18

+piFG

g18055.t1 225 YPIFG 229

79**9 PP

== domain 14 score: 6.1 bits; conditional E-value: 0.0084

round-2-all-seq-for-round-3-input-by-hmmalign 14 fpiFGKe 20

+piFGK+

g18055.t1 239 YPIFGKQ 245

79****8 PP

== domain 15 score: 4.8 bits; conditional E-value: 0.022

round-2-all-seq-for-round-3-input-by-hmmalign 14 fpiFGKe 20

+piFGK+

g18055.t1 253 YPIFGKH 259

79****6 PP

== domain 16 score: 9.8 bits; conditional E-value: 0.00058

round-2-all-seq-for-round-3-input-by-hmmalign 9 Glp.PffpiFGKe 20

+l+ P fp+FGK+

g18055.t1 263 SLVrP-FPVFGKQ 274

56767.5*****8 PP

== domain 17 score: 6.0 bits; conditional E-value: 0.0094

round-2-all-seq-for-round-3-input-by-hmmalign 14 fpiFGKe 20

+piFGK+

g18055.t1 282 YPIFGKQ 288

79****8 PP

== domain 18 score: 5.3 bits; conditional E-value: 0.015

round-2-all-seq-for-round-3-input-by-hmmalign 14 fpiFGKe 20

+p+FGKe

g18055.t1 296 YPVFGKE 302

79****8 PP

== domain 19 score: 6.0 bits; conditional E-value: 0.0094

round-2-all-seq-for-round-3-input-by-hmmalign 14 fpiFGKe 20

+piFGK+

g18055.t1 310 YPIFGKQ 316

79****8 PP

== domain 20 score: 6.0 bits; conditional E-value: 0.0094

round-2-all-seq-for-round-3-input-by-hmmalign 14 fpiFGKe 20

+piFGK+

g18055.t1 324 YPIFGKQ 330

79****8 PP

== domain 21 score: 6.0 bits; conditional E-value: 0.0094

round-2-all-seq-for-round-3-input-by-hmmalign 14 fpiFGKe 20

+piFGK+

g18055.t1 338 YPIFGKQ 344

79****8 PP

== domain 22 score: 6.0 bits; conditional E-value: 0.0094

round-2-all-seq-for-round-3-input-by-hmmalign 14 fpiFGKe 20

+piFGK+

g18055.t1 352 YPIFGKQ 358

79****8 PP

== domain 23 score: 4.8 bits; conditional E-value: 0.022

round-2-all-seq-for-round-3-input-by-hmmalign 14 fpiFGKe 20

+piFGK+

g18055.t1 366 YPIFGKH 372

79****6 PP

== domain 24 score: 12.7 bits; conditional E-value: 7.5e-05

round-2-all-seq-for-round-3-input-by-hmmalign 7 DAGlp.PffpiFGKe 20

D++l+ Pf p+FGK+

g18055.t1 374 DGSLVrPF-PVFGKQ 387

899**996.*****8 PP

== domain 25 score: -0.6 bits; conditional E-value: 1.1

round-2-all-seq-for-round-3-input-by-hmmalign 14 fpiFG 18

+piFG

g18055.t1 395 YPIFG 399

79**9 PP

>> g2811.t1

# score bias c-Evalue i-Evalue hmmfrom hmm to alifrom ali to envfrom env to acc

--- ------ ----- --------- --------- ------- ------- ------- ------- ------- ------- ----

1 ! 13.9 0.1 3e-05 0.13 5 20 .. 46 60 .. 43 62 .. 0.92

2 ! 20.1 0.3 3.5e-07 0.0015 5 19 .. 66 81 .. 66 82 .. 0.93

3 ! 12.1 5.6 0.00011 0.49 5 20 .. 96 111 .. 95 111 .. 0.91

4 ! 7.5 3.1 0.0031 13 7 18 .. 120 131 .. 116 131 .. 0.93

Alignments for each domain:

== domain 1 score: 13.9 bits; conditional E-value: 3e-05

round-2-all-seq-for-round-3-input-by-hmmalign 5 eGDAGlpP.ffpiFGKe 20

+GDA l+P ++ FGK+

g2811.t1 46 KGDATLIPvPM--FGKA 60

8*********9..***7 PP

== domain 2 score: 20.1 bits; conditional E-value: 3.5e-07

round-2-all-seq-for-round-3-input-by-hmmalign 5 eGDAGlp.PffpiFGK 19

+GDAG++ P +piFGK

g2811.t1 66 KGDAGIIdPTLPIFGK 81

8******8888****9 PP

== domain 3 score: 12.1 bits; conditional E-value: 0.00011

round-2-all-seq-for-round-3-input-by-hmmalign 5 eGDAGlpP.ffpiFGKe 20

+GDAG + ffp FGK+

g2811.t1 96 KGDAG-FDiFFPFFGKQ 111

9****.99999*****8 PP

== domain 4 score: 7.5 bits; conditional E-value: 0.0031

round-2-all-seq-for-round-3-input-by-hmmalign 7 DAGlpPffpiFG 18

DAG +ffp FG

g2811.t1 120 DAGFDNFFPFFG 131

9**99******9 PP

>> g56299.t1

# score bias c-Evalue i-Evalue hmmfrom hmm to alifrom ali to envfrom env to acc

--- ------ ----- --------- --------- ------- ------- ------- ------- ------- ------- ----

1 ? 2.2 0.1 0.14 6e+02 16 20 .. 69 73 .. 69 74 .. 0.90

2 ? 2.2 0.1 0.14 6e+02 16 20 .. 88 92 .. 88 93 .. 0.90

3 ? 2.2 0.1 0.14 6.2e+02 16 20 .. 101 105 .. 101 106 .. 0.90

4 ? 2.2 0.1 0.14 6e+02 16 20 .. 114 118 .. 114 119 .. 0.90

5 ? 2.2 0.1 0.14 6e+02 16 20 .. 133 137 .. 133 138 .. 0.90

6 ? 2.2 0.1 0.14 6.2e+02 16 20 .. 146 150 .. 146 151 .. 0.90

7 ? 2.2 0.1 0.14 6e+02 16 20 .. 159 163 .. 159 164 .. 0.90

8 ? 2.2 0.1 0.14 6e+02 16 20 .. 178 182 .. 178 183 .. 0.90

9 ? 8.1 0.0 0.002 8.8 7 20 .. 197 208 .. 195 208 .. 0.90

Alignments for each domain:

== domain 1 score: 2.2 bits; conditional E-value: 0.14

round-2-all-seq-for-round-3-input-by-hmmalign 16 iFGKe 20

iFGK+

g56299.t1 69 IFGKQ 73

9***8 PP

== domain 2 score: 2.2 bits; conditional E-value: 0.14

round-2-all-seq-for-round-3-input-by-hmmalign 16 iFGKe 20

iFGK+

g56299.t1 88 IFGKQ 92

9***8 PP

== domain 3 score: 2.2 bits; conditional E-value: 0.14

round-2-all-seq-for-round-3-input-by-hmmalign 16 iFGKe 20

iFGKe

g56299.t1 101 IFGKE 105

9***8 PP

== domain 4 score: 2.2 bits; conditional E-value: 0.14

round-2-all-seq-for-round-3-input-by-hmmalign 16 iFGKe 20

iFGK+

g56299.t1 114 IFGKQ 118

9***8 PP

== domain 5 score: 2.2 bits; conditional E-value: 0.14

round-2-all-seq-for-round-3-input-by-hmmalign 16 iFGKe 20

iFGK+

g56299.t1 133 IFGKQ 137

9***8 PP

== domain 6 score: 2.2 bits; conditional E-value: 0.14

round-2-all-seq-for-round-3-input-by-hmmalign 16 iFGKe 20

iFGKe

g56299.t1 146 IFGKE 150

9***8 PP

== domain 7 score: 2.2 bits; conditional E-value: 0.14

round-2-all-seq-for-round-3-input-by-hmmalign 16 iFGKe 20

iFGK+

g56299.t1 159 IFGKQ 163

9***8 PP

== domain 8 score: 2.2 bits; conditional E-value: 0.14

round-2-all-seq-for-round-3-input-by-hmmalign 16 iFGKe 20

iFGK+

g56299.t1 178 IFGKQ 182

9***8 PP

== domain 9 score: 8.1 bits; conditional E-value: 0.002

round-2-all-seq-for-round-3-input-by-hmmalign 7 DAGlpPffpiFGKe 20

D+G+++ +FGK+

g56299.t1 197 DGGCIF--RLFGKQ 208

99****..88***8 PP
