# Supplementary material for: The flax genome reveals orbitide diversity
Source: BMC Genomics. 2022 Jul 23;23:534. doi: 10.1186/s12864-022-08735-x (PMC9308333; doi:10.1186/s12864-022-08735-x)
Supplement: Supplementary file 6 — Additional file 6: Data S5. Leader peptide HMM search output [file 12864_2022_8735_MOESM6_ESM.docx]

Alignment of leader peptides of 5 linusorb precursor proteins by MUSCLE:

G4-136N_leader --MASSAFTLALPSLGSSPSPFNGRAHVGLPP-----------VLKARKTPIVSSSK---

G3-449N_leader MAIASSTFTLALPSLGSSPSPFKGRAHIGLAP-----------VLKARKTSATTLSRETL

G14-170P_leader -MAAASSLALATASLVATGAG--GRNNAFLPSKN----KTP-NLFLNPNKTTSSTVKAVV

G11-516P_leader -MAVVSSLALTT-SLVATAA---GRNNNAFPPSSSRNNKAPADLFITPKTTTTVKAAAV-

G11-514N_leader --MAASSVPLTT-SLVATAAA--GRNNNSKTPAN---------LFLTPKTSTVKAAV---

. *:..*: ** :: : ** : .. :: :..

G4-136N_leader -------------------------------LHSTLKKHEVVDSER

G3-449N_leader ISHSSK-------------------------LHHSLLKKSGDA---

G14-170P_leader SSSSCKRPYPKGDASLFLGIDDVFGKDAVAGHDNDQDAASGQEMAA

G11-516P_leader ---SCKRPYPKG---------------AVAAATSTLSPISGKDGG-

G11-514N_leader ---SCK----------------------LSGSHHHHHQEEGSGGG-

.

Round 1 HMM search output:

Query: 5-linusorb-precursor-proteins-leader-peptides [M=78]

Scores for complete sequences (score includes all domains):

--- full sequence --- --- best 1 domain --- -#dom-

E-value score bias E-value score bias exp N Sequence Description

------- ------ ----- ------- ------ ----- ---- -- -------- -----------

3.8e-38 131.2 2.7 6.1e-38 130.5 2.7 1.3 1 g7437.t1

5.2e-33 114.7 7.7 9.9e-33 113.8 7.7 1.5 1 g24919.t1

1.8e-14 55.3 3.0 2.8e-14 54.7 3.0 1.3 1 g53356.t1

2.2e-12 48.7 3.8 4.2e-12 47.8 3.8 1.5 1 g33422.t1

1.9e-07 32.8 0.5 3.6e-07 31.9 0.5 1.5 1 g51734.t1

------ inclusion threshold ------

0.046 15.6 1.7 0.086 14.7 1.7 1.5 1 g18055.t1

0.1 14.4 1.4 0.13 14.2 1.4 1.2 1 g39692.t1

0.47 12.3 3.1 0.68 11.8 3.1 1.3 1 g56299.t1

The top 4 hits are original inputs, and the 5th hit is as follows:

>> g51734.t1

# score bias c-Evalue i-Evalue hmmfrom hmm to alifrom ali to envfrom env to acc

--- ------ ----- --------- --------- ------- ------- ------- ------- ------- ------- ----

1 ! 31.9 0.5 4.8e-11 3.6e-07 2 60 .. 1 52 [. 1 84 [. 0.83

Alignments for each domain:

== domain 1 score: 31.9 bits; conditional E-value: 4.8e-11

5-leader-peptides 2 aaaSslaLataSLvataapGRnnaalppknkaPdlflapkttttskakavScKrPyPkG 60

+ aSsla + aSLv taa G + +p++n +P+ fla +++t+ +Sc P G

g51734.t1 1 MMASSLAVSAASLVVTAA-G--TNVFPSRN-TPNFFLANNKSTSPLKPIISCH---PGG 52

679***************.4..56899988.**************99999**9...655 PP

Round 1 retrieves 1 new protein as significant hit (“!” and the independent E-value<0.01).

Extract the signal peptide region of the new protein g51734, i.e. the 1 – 52 aa region aligned with the profile HMM shown in the above hit, and combine it with the signal peptides of the original 5 linusorb precursor proteins to build a profile HMM for round 2.

Round 2 HMM search output:

Query: 6-leader-peptides-from-round-1 [M=70]

Scores for complete sequences (score includes all domains):

--- full sequence --- --- best 1 domain --- -#dom-

E-value score bias E-value score bias exp N Sequence Description

------- ------ ----- ------- ------ ----- ---- -- -------- -----------

1.9e-32 113.2 2.9 3.2e-32 112.5 2.9 1.4 1 g7437.t1

1.3e-25 91.4 6.9 2e-25 90.8 6.9 1.3 1 g24919.t1

6.8e-19 69.8 0.6 1e-18 69.2 0.6 1.3 1 g51734.t1

8.1e-17 63.2 3.8 1.4e-16 62.4 3.8 1.3 1 g53356.t1

6.6e-16 60.3 1.5 1.1e-15 59.6 1.5 1.3 1 g33422.t1

------ inclusion threshold ------

0.15 14.3 1.6 0.2 13.9 1.6 1.3 1 g39692.t1

1.1 11.4 2.9 1.7 10.8 2.9 1.3 1 g18055.t1

3.4 9.9 4.9 1.5 11.0 2.3 1.8 2 g11605.t1

Round 2 does not retrieve any new and significant sequences above the inclusion threshold.

Nevertheless, among the 4 non-significant hits below the inclusion threshold from both rounds, there are 2 linusorb-like proteins: g18055 and g56299. The other 2 proteins, g11605 and g39692, are analysed by RADAR. The former does not contain any profile-matching repeats, whereas the latter has two profile-matching repeats (conserved flanking signatures underlined):

>g39692.t1

MASSAFSTGLLSLGSSPSSPRPDTTPAMTSFPSKSSSMVFSSNRKQ

QQAQAVNSKRGDGAVILVNIFGK

QQAQDESDATMWGGVDSFGKTA
